# Genome-wide characterization and expression analysis of the *Dof* gene family related to abiotic stress in watermelon

**DOI:** 10.7717/peerj.8358

**Published:** 2020-02-17

**Authors:** Yong Zhou, Yuan Cheng, Chunpeng Wan, Jingwen Li, Youxin Yang, Jinyin Chen

**Affiliations:** 1 Key Laboratory of Crop Physiology, Ecology and Genetic Breeding, Ministry of Education, Jiangxi Agricultural University, Nanchang, Jiangxi, China; 2 Jiangxi Engineering Laboratory for the Development and Utilization of Agricultural Microbial Resources, College of Bioscience and Bioengineering, Jiangxi Agricultural University, Nanchang, Jiangxi Province, China; 3 Zhejiang Academy of Agricultural Sciences, State Key Laboratory Breeding Base for Zhejiang Sustainable Pest and Disease Control, Institute of Vegetables, Hanghzou, Zhejiang, China; 4 Jiangxi Key Laboratory for Postharvest Technology and Nondestructive Testing of Fruits & Vegetables, Collaborative Innovation Center of Post-Harvest Key Technology and Quality Safety of Fruits and Vegetables, College of Agronomy, Jiangxi Agricultural University, Nanchang, Jiangxi, China; 5 Pingxiang University, Pingxiang, Jiangxi, China

**Keywords:** Watermelon, Dof, Phylogenetic analysis, Expression profile, Abiotic stress

## Abstract

The plant DNA-binding with one finger (Dof) gene family is a class of plant-specific transcription factors that play vital roles in many biological processes and stress responses. In the present study, a total of 36 *ClDof* genes were identified in the watermelon genome, which were unevenly distributed on 10 chromosomes. Phylogenetic analysis showed that the ClDof proteins could be divided into nine groups, and the members in a particular group had similar motif arrangement and exon–intron structure. Synteny analysis indicated the presence of a large number of syntenic relationship events between watermelon and cucumber. In promoter analysis, five kinds of stress-related and nine kinds of hormone-related *cis*-elements were identified in the promoter regions of *ClDof* genes. We then analyzed the expression patterns of nine selected *ClDof* genes in eight specific tissues by qRT-PCR, and the results showed that they have tissue-specific expression patterns. We also evaluated the expression levels of 12 selected *ClDof* genes under salt stress and ABA treatments using qRT-PCR. As a result, they showed differential expression under these treatments, suggesting their important roles in stress response. Taken together, our results provide a basis for future research on the biological functions of *Dof* genes in watermelon.

## Introduction

DNA binding with one finger (Dof) proteins are a group of plant-specific transcription factors widely present in plants, while there has been no report about them in other eukaryotes such as humans and yeast (Azam et al., 2018; Gupta et al., 2015). Genome-wide surveys showed that the *Dof* family genes are widely distributed in the genomes of various plant species. For example, as model plants, *Arabidopsis* and rice include 36 and 30 *Dof* genes in their genomes, respectively (Lijavetzky, Carbonero & Vicente-Carbajosa, 2003). In addition, it has been reported that there are 25 *Dof* genes in peach (*Prunus persica*) (Chen et al., 2017), 29 in eggplant (*Solanum melongena*) (Wei et al., 2018), 33 in pepper (*Capsicum annuum*) (Kang et al., 2016; Wu et al., 2016), 34 in tomato (*Solanum lycopersicum*) (Cai et al., 2013), 36 in cucumber (*Cucumis sativus*) (Wen et al., 2016), 45 in cassava (*Manihot esculenta*) (Zou, Zhu & Zhang, 2019), 45 in pear (*Pyrus bretschneideri*) (Liu et al., 2020), and 60 in apple (*Malus domestica*) (Zhang et al., 2018). These reports revealed that the Dof proteins are characterized by the highly conserved Dof domain in their N-terminal regions, which is composed of about 52 amino acids with a Cys2/Cys2 zinc finger structure (Umemura et al., 2004; Yanagisawa, 2002). The Dof domain specifically recognizes and combines with a T/AAAAG core sequence in the promoters of target genes (Noguero et al., 2013; Umemura et al., 2004). In addition, the Dof proteins also contain a variable transcriptional activation domain at their C-terminus. The N- and C-terminal regions of the Dof proteins contribute to their bi-functional roles in DNA binding and protein–protein interactions to regulate the expression of the target genes (Gupta et al., 2015; Noguero et al., 2013).

As the first identified *Dof* gene, *ZmDof1* was found to play a role in light-regulated gene expression and affect light response and nitrogen assimilation (Yanagisawa & Izui, 1993; Yanagisawa & Sheen, 1998). Subsequently, a large number of *Dof* genes were reported to be involved in various plant-specific biological processes, such as seed germination (Boccaccini et al., 2014; Gualberti et al., 2002; Santopolo et al., 2015), fruit ripening (Feng et al., 2016), flowering time control (Li et al., 2009; Liu et al., 2020; Wu et al., 2017), and responses to plant hormones (Boccaccini et al., 2016; Lorrai et al., 2018; Qin et al., 2019; Rymen et al., 2017), as well as various stress responses (Su et al., 2017; Zang et al., 2017). Moreover, some *Dof* genes can play multifaceted roles in regulating plant development and stress responses. For example, overexpression of *Arabidopsis CDF3* could contribute to higher tolerance to drought, cold and osmotic stress and lead to late flowering, suggesting that it is involved in both flowering time control and abiotic stress tolerance (Corrales et al., 2017). In tomato, overexpression of a *Dof* gene *TDDF1* induced early flowering by increasing the expression of flowering-time control genes, and the transgenic plants also displayed higher resistance to drought, salt, and late blight caused by *Phytophthora infestans* (Ewas et al., 2017). In rice, salt stress repressed the expression of *OsDOF15* in roots, and overexpression of *OsDOF15* reduced the sensitivity of roots to salt stress via limiting ethylene biosynthesis, suggesting that OsDOF15-mediated ethylene biosynthesis may be involved in the inhibition of primary root elongation by salt stress (Qin et al., 2019). These findings demonstrate that the Dof proteins are involved in diverse biological processes and play important roles in the growth and development of plants.

Although the *Dof* gene family has been comprehensively analyzed and functionally characterized in a number of plant species, little is known about this gene family in watermelon, an economically important fruit crop cultivated worldwide. In this study, we characterized the *Dof* family genes in watermelon by analyses of their phylogenetic relationships, conserved motifs, gene structures and chromosomal localizations. In addition, the expression profiles of the selected *Dof* genes in different tissues and under salt or ABA treatment conditions were also examined. Our findings may lay a foundation for future functional analysis of *Dof* genes in watermelon.

## Materials and Methods

### Genome-wide identification and protein properties of Dof family in watermelon

To identify the watermelon *Dof* family genes, HHM profile of the Dof domain (PF02701) was used as a query to perform an HMMER search against the watermelon proteome, which was downloaded in watermelon (97103) v1 genome from the cucurbit genomics database (CuGenDB; http://cucurbitgenomics.org). A comprehensive search was also performed by using the amino acid sequences of *Arabidopsis* and rice Dof proteins from a previous study (Lijavetzky, Carbonero & Vicente-Carbajosa, 2003), which were obtained from the TIGR database (https://rice.plantbiology.msu.edu/) and the TAIR database (https://www.arabidopsis.org/), respectively. The putative sequences were submitted to Pfam (http://pfam.sanger.ac.uk/) and SMART (http://smart.embl-heidelberg.de/) for checking the presence of the Dof domain.

### Sequence analyses and phylogenetic tree construction

The biochemical features including molecular weight (MW) and isoelectric point (pI) of all Dof proteins were determined by ProtParam server (http://web.expasy.org/protparam/). The subcellular localizations of the watermelon Dof proteins were predicted with CELLO v2.5 tool (http://cello.life.nctu.edu.tw/). The MEME tool (http://meme-suite.org/tools/meme) was used to predict and analyze the conserved motifs of watermelon Dof proteins with the maximum number of motifs being set as 10, and other parameters were set as default. The predicted motifs were further confirmed by searching against InterProScan (http://www.ebi.ac.uk/interpro/search/sequence-search/), and structure schematic diagrams were illustrated using the TBtools software (Chen et al., 2018). The coding region sequences (CDS) and genomic DNA (gDNA) sequences of *ClDof* genes were downloaded from watermelon (97103) v1 genome database (http://cucurbitgenomics.org/organism/1), and then the exon–intron structures of *ClDof* genes were displayed by the GSDS tool (Gene Structure Display Server, http://gsds.cbi.pku.edu.cn/) based on the alignment of CDSs with the corresponding gDNA sequences. For gene ontology (GO) analysis, the annotations of *ClDof* genes were obtained from watermelon (97103) v1 genome database and visualized with the WEGO program (http://wego.genomics.org.cn/). For promoter analysis, we determined the putative promoter sequence for each *ClDof* gene, which was defined as the upstream 1,500 bp region of the transcription start site (ATG), and analyzed the stress-related and hormone-related *cis*-elements using the PlantCARE tool (http://bioinformatics.psb.ugent.be/webtools/plantcare/html/). For phylogenetic tree construction, the Dof proteins of watermelon, cucumber, rice and *Arabidopsis* were aligned by Clustal Omega with default parameters. The Dof protein IDs of above species were listed in Table S1. Then, the MEGA program (v7.0) was used to construct a Neighbor-Joining tree with parameters of 1,000 bootstrap replicates and pairwise deletion.

### Chromosomal location, gene duplication, and synteny analysis

The chromosomal location information of watermelon *Dof* genes was obtained from the watermelon genome database, and MapChart was used to display the physical positions of all *ClDof* genes along each chromosome. Gene duplication and synteny of *Dof* genes from watermelon and cucumber were examined using multiple collinear scanning toolkits (MCScanX) software with default parameters as previously reported (You et al., 2018).

### Plant materials and treatments

Seeds of the watermelon cultivar “Xinong 8” (*Citrullus lanatus* L.) were first sterilized and germinated in an incubator (28 °C). Then, the germinated seeds were sown in pots and cultivated under a 12 h day/12 h night cycle (25 °C/19 °C, day/night temperature cycle) until the seedlings developed to four leaves. Uniformly developed four-leaf-stage watermelon plants were then exposed to NaCl (200 mm) and ABA (100 µm) treatments for 0 h, 1 h, 3 h, 9 h and 24 h. All leaves from watermelon plants were collected and rapidly frozen in liquid nitrogen and stored at –80 °C until RNA extraction.

### RNA extraction and quantitative real-time PCR (qRT-PCR)

Total RNA was isolated using the total RNA Miniprep Kit (Axygen Biosciences, Union City, CA, USA) according to the manufacturer’s protocol. Then, RNase-free DNase I was added in RNA solution to remove any contaminated gDNA. First-strand cDNA synthesis was carried out following the manufacturer’s procedure (ReverTra Ace qPCR-RT Kit, Toyobo, Japan). Primers were designed using Primer Premier 5.0 software (Table S2). The qRT-PCR was performed on an CFX96 instrument (Bio-Rad, Alfred Nobel Drive Hercules, CA, USA) using SYBR Green qPCR kits (TaKaRa, Japan). The watermelon constitutive actin gene (Cla007792) was used as the endogenous control (Zhou et al., 2018b). The PCR amplification conditions included an initial heat-denaturing step at 95 °C for 3 min, followed by 40 cycles of 30 s at 95 °C, 30 s at 58 °C, and 1 min at 72 °C. Relative expression levels were calculated using the 2^−ΔΔCt^ method (Livak & Schmittgen, 2001), and each treatment included three independent biological replicates and three technical replicates. Data were statistically analyzed by one-way ANOVA using SPSS 19.0 software, and Tukey’s multiple range tests were used to detect significant treatment differences (*P* < 0.05).

## Results

### Genome-wide identification of *Dof* family genes in watermelon

A total of 36 *Dof* genes were identified and named as *ClDof1*–*36* according to their order on the chromosomes. Detailed information including the CDS length, protein length, predicted MW and pI of each gene is listed in Table 1. The amino acid sequences and gene sequences of ClDof members are listed in Tables S3–S5. These genes had CDS lengths ranging from 492 bp (*ClDof1*) to 1575 bp (*ClDof33*), and encoded proteins ranging from 163 to 524 amino acid residues, with the predicted MW varying from 17.64 to 56.71 kDa. The pIs of the ClDof proteins ranged from 5.00 (ClDof30) to 9.95 (ClDof13). The CELLO v2.5 tool was used to analyze the subcellular localization of ClDof proteins. The results showed that nearly all ClDof proteins were localized in the nucleus, with the exception of ClDof6, which possibly had a nuclear and extracellular localization (Table 1). The GO annotation results indicated that ClDof proteins were assigned into three major categories and 18 subcategories (Table S6; Fig. S1).

**Table 1:** Members of *Dof* family genes identified in watermelon.

Gene name	Gene ID	Map position (bp)	CDS length (bp)	Protein length (aa)	MW (kDa)	*p*I	Subcellular location
*ClDof1*	Cla000091	Chr0:12921851–12922342	492	163	17.64	8.21	Nuclear
*ClDof2*	Cla000604	Chr0:24087372–24088166	795	264	29.22	8.41	Nuclear
*ClDof3*	Cla004880	Chr1:83833–84684	852	283	30.46	8.4	Nuclear
*ClDof4*	Cla011343	Chr1:1447591–1449038	831	276	29.57	7.72	Nuclear
*ClDof5*	Cla000975	Chr1:10830770–10831984	1,011	336	37.74	7.31	Nuclear
*ClDof6*	Cla001812	Chr1:26447800–26448528	729	242	24.73	8.34	Nuclear/extracellular
*ClDof7*	Cla001818	Chr1:26513973–26514995	1,023	340	35.45	9.21	Nuclear
*ClDof8*	Cla014094	Chr1:28161694–28162635	942	313	33.83	8.26	Nuclear
*ClDof9*	Cla001373	Chr1:31447086–31447871	786	261	29.33	8.84	Nuclear
*ClDof10*	Cla009627	Chr1:31658215–31659085	717	238	25.84	8.84	Nuclear
*ClDof11*	Cla009628	Chr1:31665641–31666539	729	242	26.88	9.49	Nuclear
*ClDof12*	Cla009692	Chr1:32112455–32112961	507	168	19.04	8.81	Nuclear
*ClDof13*	Cla013297	Chr2:30590643–30592400	1,020	339	37.52	9.95	Nuclear
*ClDof14*	Cla000540	Chr2:31118585–31119331	747	248	27.29	8.73	Nuclear
*ClDof15*	Cla008250	Chr3:1516113–1517286	1,026	341	37.25	9.31	Nuclear
*ClDof16*	Cla005059	Chr3:2677903–2678760	858	285	31.75	8.39	Nuclear
*ClDof17*	Cla019672	Chr3:8389380–8389913	534	177	20.21	7.13	Nuclear
*ClDof18*	Cla019705	Chr3:8782843–8783751	909	302	33.57	7.46	Nuclear
*ClDof19*	Cla019706	Chr3:8791610–8792131	522	173	18.69	9.22	Nuclear
*ClDof20*	Cla018219	Chr4:19894774–19896290	813	270	29.93	9.9	Nuclear
*ClDof21*	Cla018604	Chr4:23659963–23661769	1,308	435	47.56	7.04	Nuclear
*ClDof22*	Cla021140	Chr5:723346–723861	516	171	18.06	8.99	Nuclear
*ClDof23*	Cla004274	Chr5:9417748–9418525	678	225	24.96	8.32	Nuclear
*ClDof24*	Cla010192	Chr5:31339279–31340779	1,296	431	47.33	8.11	Nuclear
*ClDof25*	Cla006705	Chr6:3496040–3496858	819	272	29.94	8.26	Nuclear
*ClDof26*	Cla019034	Chr6:24515454–24516428	975	324	34.96	8.08	Nuclear
*ClDof27*	Cla019107	Chr6:25139609–25141772	1,395	464	50.63	6.19	Nuclear
*ClDof28*	Cla004013	Chr7:3742674–3743851	969	322	34.24	9.24	Nuclear
*ClDof29*	Cla012621	Chr7:24693545–24694168	624	207	22.36	8.36	Nuclear
*ClDof30*	Cla013851	Chr8:15842719–15843486	768	255	28.77	5	Nuclear
*ClDof31*	Cla022532	Chr8:24427298–24428044	747	248	25.77	8.12	Nuclear
*ClDof32*	Cla004676	Chr9:32014839–32016085	1,077	358	39.08	8.43	Nuclear
*ClDof33*	Cla016993	Chr10:21239053–21241153	1,575	524	56.71	5.07	Nuclear
*ClDof34*	Cla002907	Chr10:21961596–21963908	1,527	508	54.74	6.06	Nuclear
*ClDof35*	Cla017622	Chr10:24621093–24621851	759	252	27.81	6.76	Nuclear
*ClDof36*	Cla017890	Chr10:27032680–27034515	1,053	350	37.19	9.85	Nuclear

### Phylogenetic characterization of the watermelon *Dof* gene family

**Figure 1:**
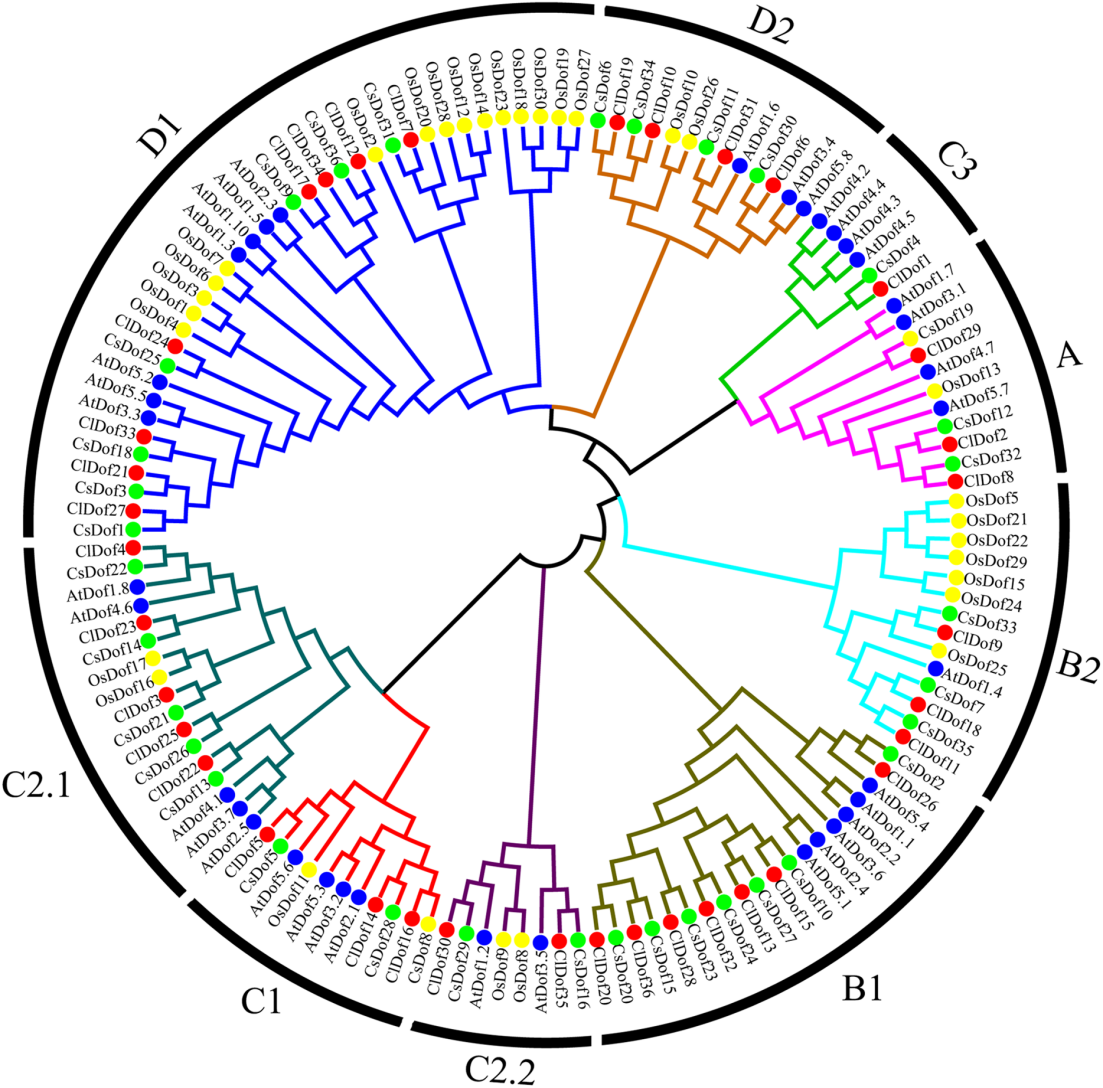
Phylogenetic relationships of Dof family proteins in watermelon, cucumber, rice and *Arabidopsis*. Different plant species are represented with different colors: watermelon (red, Cl), cucumber (green, Cs), rice (yellow, Os), and Arabidopsis (blue, At).

To study the evolutionary relationship of *Dof* family genes between watermelon and other plants, a phylogenetic tree based on multiple sequence alignment was constructed by using the amino acid sequences of ClDofs together with those from cucumber (CsDofs) (Wen et al., 2016), rice (OsDofs) and *Arabidopsis* (AtDofs) (Lijavetzky, Carbonero & Vicente-Carbajosa, 2003). The phylogenetic tree showed that these Dof proteins could be classified into nine groups, namely A, B1, B2, C1, C2.1, C2.2, C3, D1 and D2, with well-supported bootstrap values (Fig. 1). Nearly all groups included ClDofs, CsDofs, OsDofs and AtDofs, with the exception of group C3, which comprised only dicotyledonous Dofs (ClDofs, CsDofs, and AtDofs). Besides, the numbers of ClDofs in groups A, B1, B2, C1, C2.1, C2.2, C3, D1 and D2 were 3, 7, 3, 3, 5, 2, 1, 8 and 4, respectively (Fig. 1).

### Conserved motif analysis of ClDofs

**Figure 2:**
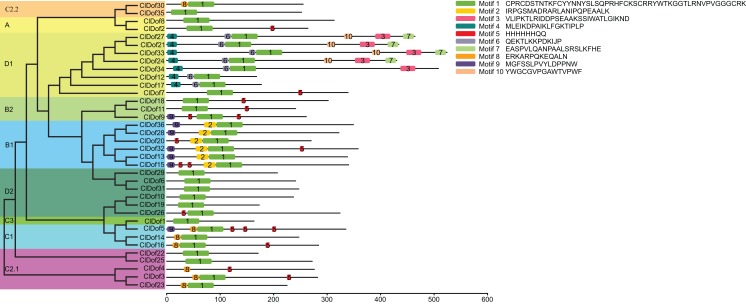
Conserved domains of ClDofs based on the evolutionary relationship. Distribution of conserved motifs in the ClDof proteins.

By using the MEME program, a total of 10 conserved motifs were identified (Fig. 2). Amongst them, motif 1 was annotated as a Dof domain, which was widely present in all ClDof proteins, with the exception of ClDof4. Some other motifs were specifically present in individual groups. For example, motif 3, 4, 6, 7 and 10 were exclusively present in the ClDofs in group D1, while motif 2 was present in all ClDofs of group B1. Besides motif 2, nearly all group B1 ClDofs included motif 9 (except for ClDof20). In addition, motif 8 was present in all group C1 ClDofs, as well as some ClDofs in groups C2.1 and C2.2 (Fig. 2). It is worth noting that besides motif 1 and 8, three motif 5 and one motif 9 were also present in ClDof5 of group C1.

**Figure 3:**
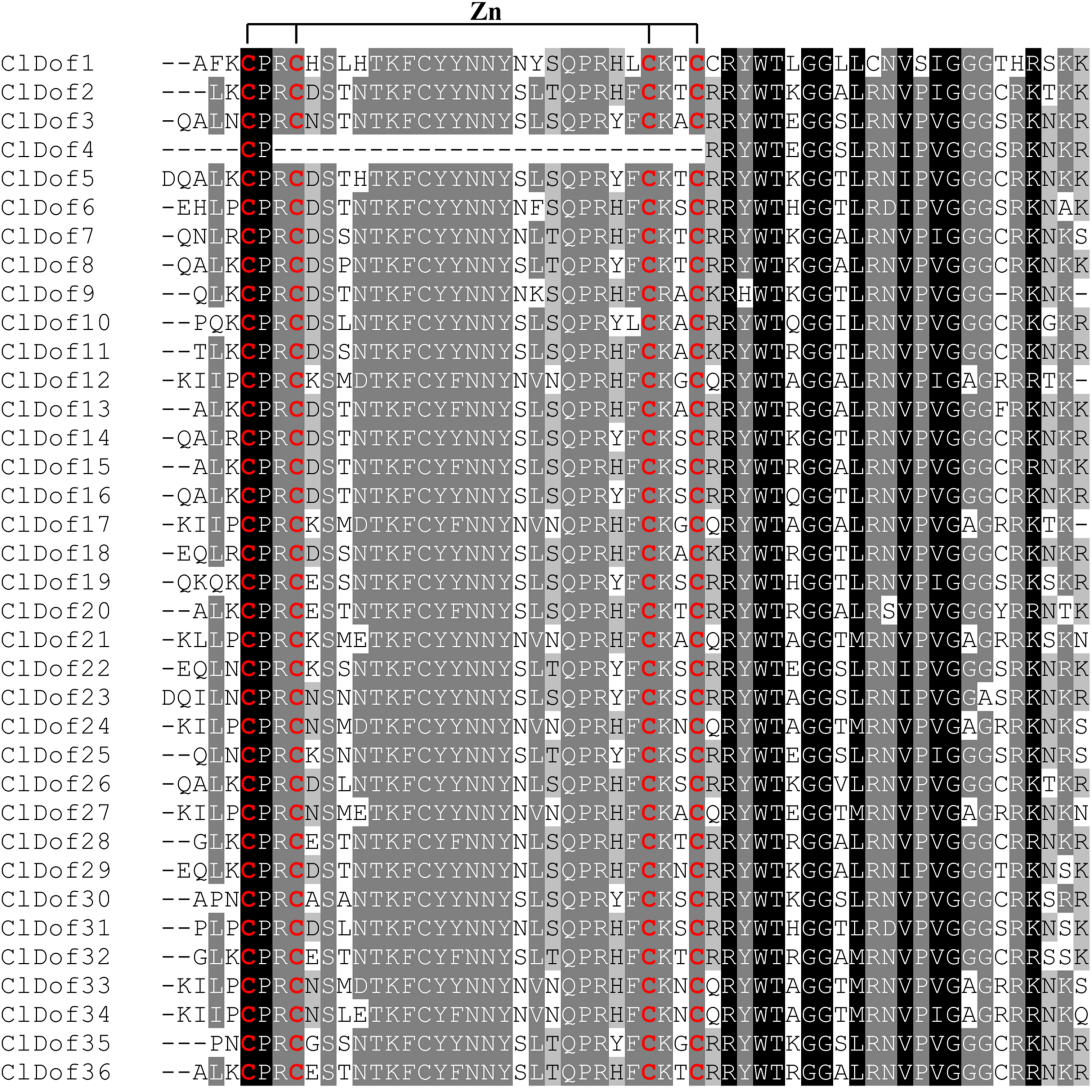
Dof domain sequence alignment of watermelon Dof proteins. The Dof DNA-binding domains among watermelon Dof proteins were aligned and the four Cys residues associated with zinc finger structure of the ClDofs are colored in red.

To better understand the structural features of Dof domain, multiple sequence alignment of the Dof domain sequences of ClDofs was carried out. As a result, the Dof domain of ClDofs was highly conserved, and nearly all ClDof proteins harbored the four Cys residues associated with zinc finger structure, with the exception of ClDof4 (Fig. 3), which may result in the divergence of its function from that of other ClDofs.

### Exon–intron arrangement analysis of *Dof* family genes in watermelon

**Figure 4:**
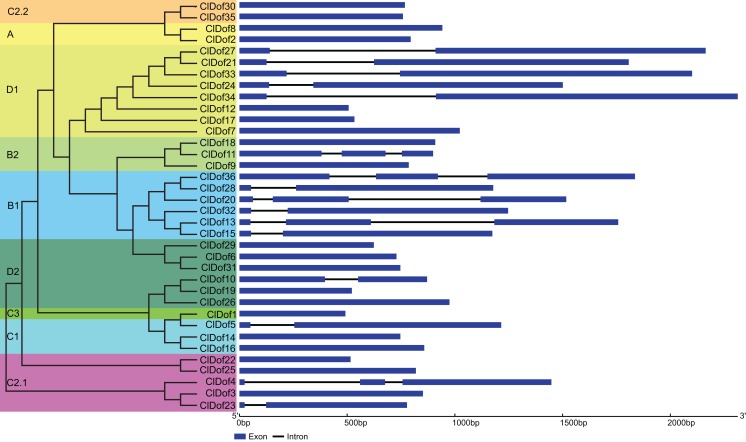
Exon–intron structure of *ClDof* genes based on the evolutionary relationship.

The CDS and gDNA sequences of the 36 *ClDof* genes were used to examine the distribution of exons and introns. As a result, most of the *ClDof* genes (20 out of 36) contained no introns, 11 *ClDof* genes (*ClDof5*, *ClDof10*, *ClDof15*, *ClDof23*, *ClDof27*, *ClDof28*, *ClDof21*, *ClDof24*, *ClDof32*, *ClDof33* and *ClDof34*) had one intron each, whereas five *ClDof* genes (*ClDof4*, *ClDof11*, *ClDof13*, *ClDof20* and *ClDof36*) possessed two introns (Fig. 4).

### Chromosome distribution, gene duplication and synteny analysis of *ClDof* genes

**Figure 5:**
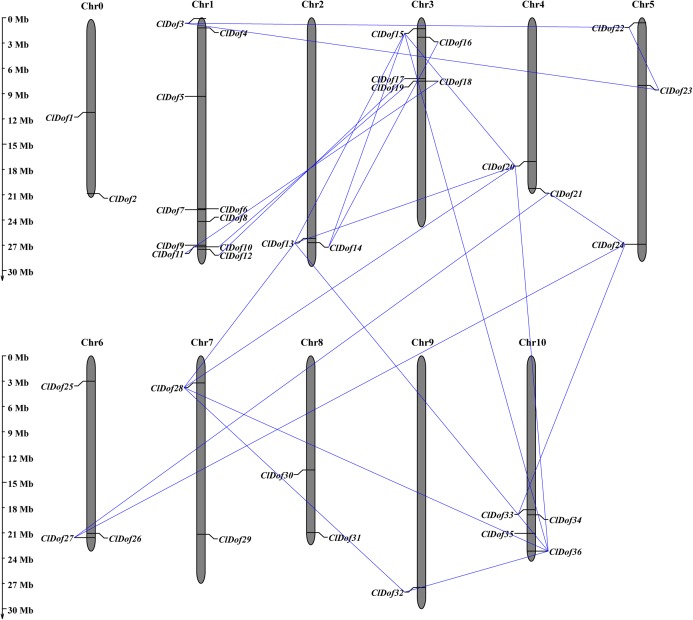
Chromosomal distribution of *ClDof* genes in watermelon genome. The segmental duplication genes are connected by lines.

Using the MapInspect program, a total of 34 *ClDof* genes were mapped on 10 of the 12 chromosomes in watermelon genome, while *ClDof1* and *ClDof2* were located on chromosome 0 (Fig. 5). In detail, there were 10, 2, 5, 2, 3, 3, 2, 2, 1 and 4 *ClDof* genes on chromosome 1, 2, 3, 4, 5, 6, 7, 8, 9 and 10, respectively. Moreover, the gene duplication events were analyzed using the MCScanX program, and a total of 20 *ClDof* genes exhibited segmental duplication, which made up 21 pairs of segmental duplication genes (Fig. 5).

**Figure 6:**
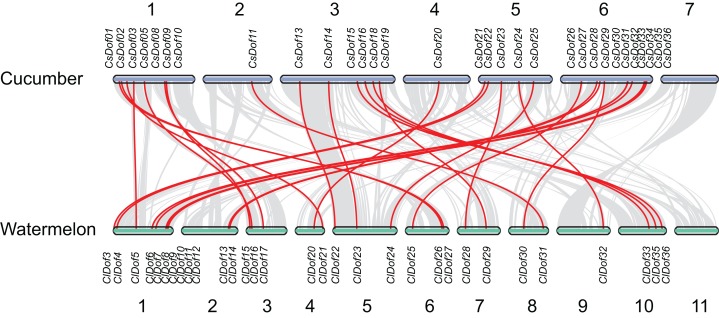
Synteny analysis of *Dof* genes from watermelon and cucumber genomes. Gray lines in the background indicate the collinear blocks within watermelon and cucumber genomes. The orthologous gene pairs are linked with red lines.

To reveal the orthologous relationships of *Dof* genes on chromosomes between watermelon and cucumber genomes, a comparative analysis was performed between *Dof* genes in watermelon and cucumber by MCScanX. A total of 31 orthologous gene pairs were identified between watermelon and cucumber (Fig. 6), indicating close relationships between *ClDof* and *CsDof* genes.

### Promoter *cis*-elements of the *ClDof* genes

**Figure 7:**
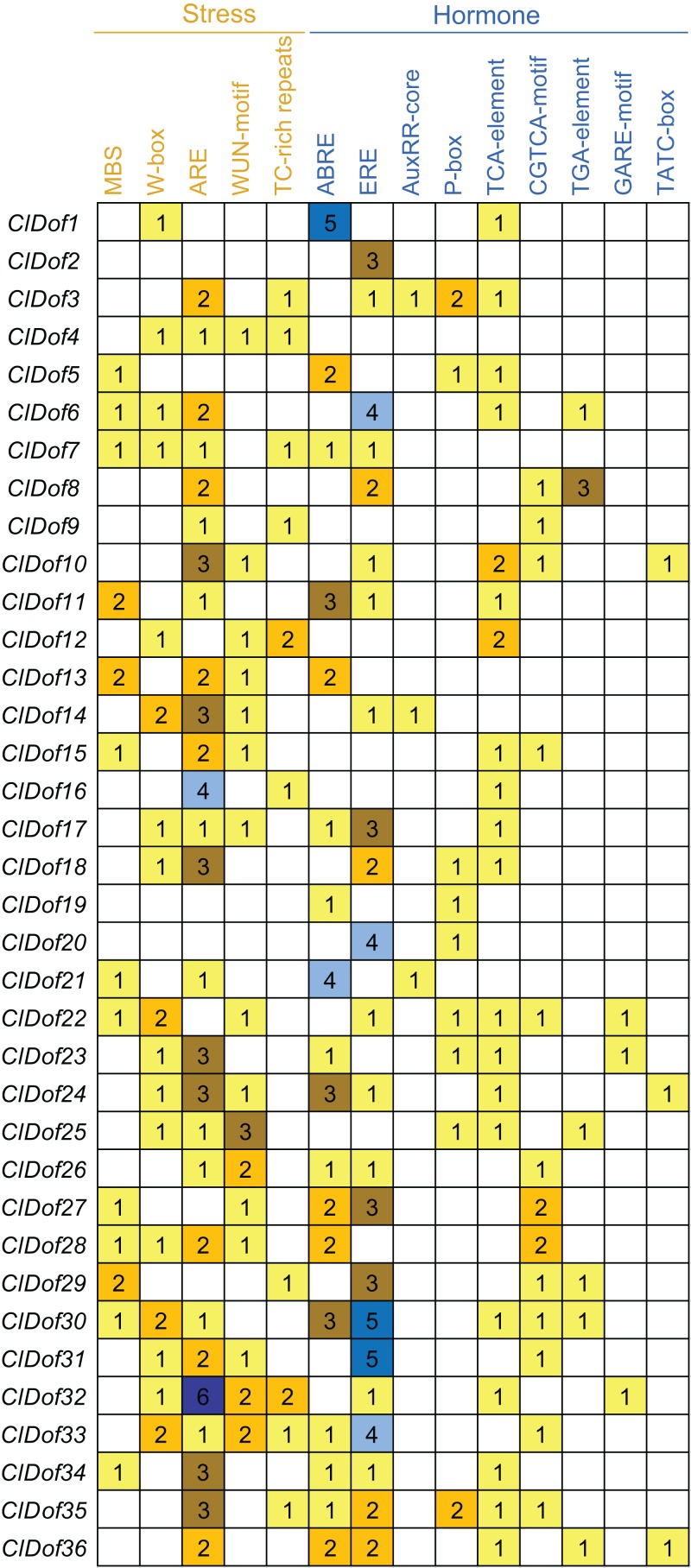
Distribution of stress- and hormone-related *cis*-elements in the promoter regions of *ClDof* genes.

To assess the transcriptional regulation and potential functions of the *ClDof* genes, the *cis*-elements in the promoter regions of the *ClDof* genes were investigated and described in Fig. 7. Five kinds of stress-related and nine kinds of hormone-related *cis*-elements were identified in this study (Fig. 7). The ARE element was the most abundant among the stress-related *cis*-elements, and the promoter regions of 28 *ClDof* genes harbored the ARE element (Fig. 7), which is essential for the anaerobic induction. Other four stress-related *cis*-elements, including W-box, WUN-motif, MBS and TC-rich repeats, were found in 17, 16, 13 and 10 promoter regions of *ClDof* genes, respectively. Furthermore, various hormone-related *cis*-elements were also identified among the promoters of *ClDof* genes (except for *ClDof4*), including abscisic acid (ABA)-responsive element (ABRE), ethylene-responsive element (ERE), salicylic acid (SA)-responsive element (TCA-element), methyl jasmonate (MeJA)-responsive element (CGTCA-motif), auxin-responsive elements (AuxRR-core and TGA-element), and gibberellin-responsive elements (P-box, GARE-motif and TATC-box) (Fig. 7). These findings indicated that *ClDof* genes might play certain roles in various stress responses and hormone signaling.

### Tissue-specific expression profiles of the *ClDof* genes

**Figure 8:**
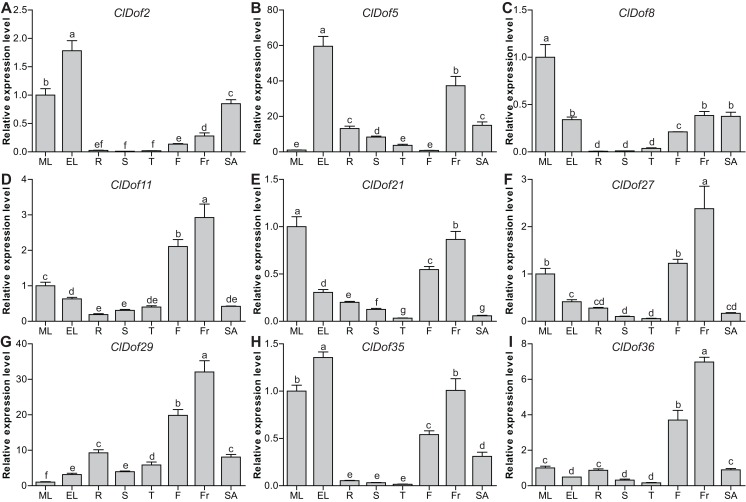
Expression profiles of nine selected *ClDof* genes (A–I) in various tissues determined by qRT-PCR. ML, mature leaves; EL, expanding leaves; R, roots; S, stems; T, tendrils; F, flowers; Fr, fruits; SA, stem apexes. Different letters above the bars stand for significant differences (Tukey’s multiple range tests, *P* < 0.05) between different treatment times.

To evaluate the functions of *ClDof* genes in the growth and development of watermelon, the expression of nine selected *ClDof* genes in different tissues (mature and expanding leaves, roots, stems, stem apexes, flowers and fruits) was examined with qRT-PCR. Most *ClDof* genes were highly expressed in flowers and/or fruits, such as *ClDof11*, *ClDof21*, *ClDof27*, *ClDof29*, *ClDof35* and *ClDof36* (Fig. 8; Table S7), suggesting that they may function in flower and fruit development of watermelon. In addition, *ClDof2*, *ClDof5*, *ClDof8*, *ClDof21* and *ClDof35* displayed the highest expression in leaves, and relatively lower expression in other tissues, especially roots, stems, and tendrils (Fig. 8). Besides expanding leaves, *ClDof5* also showed relatively higher expression in fruits as compared with other tissues, while its expression was extremely low in flowers. Finally, nearly all *ClDof* genes exhibited moderate transcript abundance in stem apexes (Fig. 8), implying their possible roles in stem apex development of watermelon.

### Expression profiles of *ClDof* genes in response to salt stress and ABA treatment

**Figure 9:**
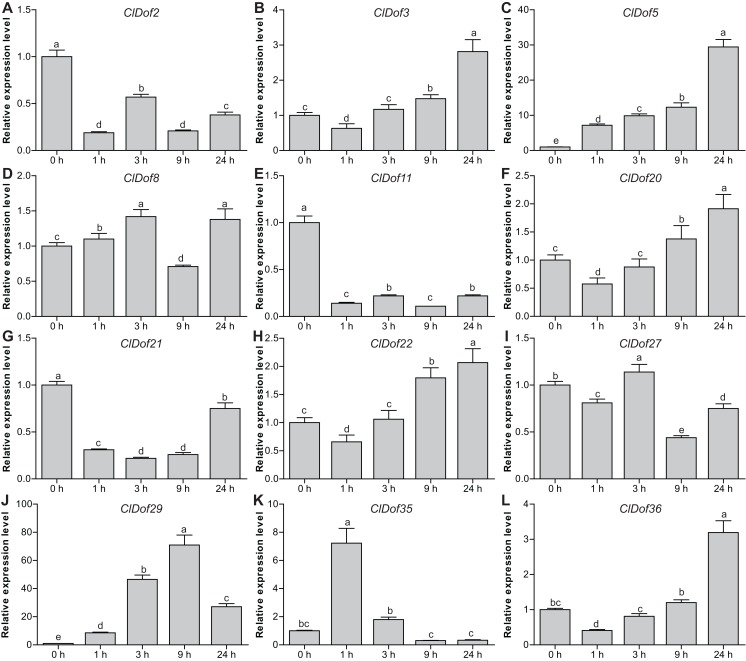
Expression profiles of twelve selected *ClDof* genes (A–L) in response to salt stress determined by qRT-PCR. Different alphabets above the bars indicate significant differences (Tukey’s multiple range tests, *P* < 0.05) between different treatment times.

**Figure 10:**
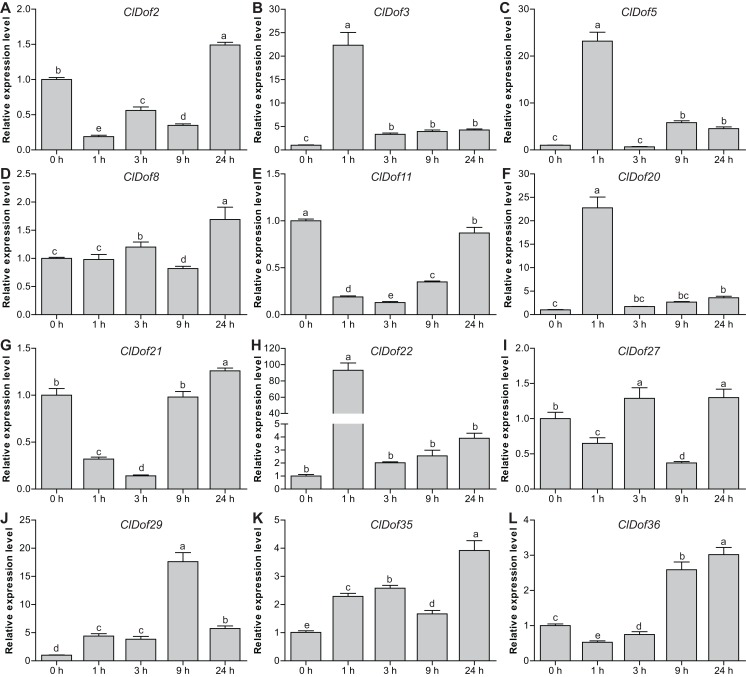
Expression profiles of twelve selected *ClDof* genes (A–L) under ABA treatment determined by qRT-PCR. Different letters above the bars indicate significant differences (Tukey’s multiple range tests, *P* < 0.05) between different treatment times.

To reveal the possible roles of *ClDof* genes in response to abiotic stress, we determined the expression levels of 12 selected *ClDof* genes under salt stress and ABA treatments using qRT-PCR. Under salt stress, nine *ClDof* genes (*ClDof3*, *ClDof5*, *ClDof8*, *ClDof20*, *ClDof22*, *ClDof27*, *ClDof29*, *ClDof35* and *ClDof36*) were up-regulated at certain time points (Fig. 9; Table S7). Amongst them, *ClDof5* was induced gradually and reached the highest transcript abundance at 24 h, while the expression of *ClDof36* showed a decrease at early time point (1 h) and then gradually increased until 24 h (Fig. 9). In addition, three *ClDof* genes (*ClDof2*, *ClDof11* and *ClDof21*) were down-regulated across all time points under salt stress, indicating their negative roles in response to salt stress. It should be noted that the expression levels of *ClDof3*, *ClDof8*, *ClDof20*, *ClDof22*, *ClDof27* and *ClDof35* were significantly decreased at some time points (Fig. 9). We also determined whether these *ClDof* genes are regulated by ABA. As shown in Fig. 10 (Table S7), the expression of all detected *ClDof* genes was significantly altered by ABA treatment, and the expression of five *ClDof* genes (*ClDof2*, *ClDof11*, *ClDof21*, *ClDof27* and *ClDof3*6) showed a decreasing tendency at early time points (1 h and 3 h) and finally increased at the late time points (24 h). It is worth noting that the expression of four *ClDof* genes (*ClDof3*, *ClDof5*, *ClDof20* and *ClDof22*) was dramatically induced at 1 h, followed by sharp decreases thereafter. These results indicated that the *ClDof* genes may play crucial roles in stress responses.

## Discussion

In the present study, we systematically predicted and identified 36 *Dof* genes in the watermelon genome (Table 1). The number of *ClDof* genes is similar to that in many other plant species, such as pepper (33 genes) (Kang et al., 2016; Wu et al., 2016), tomato (34 genes) (Cai et al., 2013), potato (35 genes) (Venkatesh & Park, 2015), foxtail millet (35 genes) (Zhang et al., 2017), cucumber (36 genes) (Wen et al., 2016), chickpea (37 genes) (Nasim et al., 2016), and pigeonpea (38 genes) (Malviya et al., 2015), suggesting that *Dof* genes usually form multigene families in plants. Duplication events were found to be the primary driving force for the evolution of *Dof* genes. For example, two pairs of tandemly duplicated genes and six pairs of segmentally duplicated genes were identified in the cucumber genome (Wen et al., 2016). In poplar, up to 49% (20 out of 41) of *PtrDof* genes were found to be located in both segmental and tandem duplicated regions (Wang et al., 2017). In apple, a total of 57 and 18 *MdDof* genes were located in segmental and tandem duplicated regions, respectively, and 13 *MdDof* genes were both segmentally and tandemly duplicated genes (Hong et al., 2019). In this study, more than half of the *ClDof* genes (20 out of 36) exhibited segmental duplications, while no tandem duplication was identified in watermelon chromosomes, suggesting that segmental duplication has been predominant in the expansion of the *Dof* genes in watermelon. Similar results have also been reported in other plants such as cotton (Li et al., 2018).

The phylogenetic results revealed that ClDofs could be clearly divided into nine groups: A, B1, B2, C1, C2.1, C2.2, C3, D1 and D2 (Fig. 1), which is consistent with the results in eggplant (Wei et al., 2018), pear (Liu et al., 2020), *Arabidopsis* and rice (Lijavetzky, Carbonero & Vicente-Carbajosa, 2003). Besides, each of the watermelon *Dof* genes has at least one homologous gene in *Arabidopsis* (Fig. 1), implying that *Dof* genes might play similar roles in watermelon as their homologs in *Arabidopsis*. In addition, nearly all ClDofs had a common Dof motif (motif 1), but there were also some unique motifs in certain groups with nearly conserved motif compositions (Fig. 2). However, gain or loss of certain motifs was observed between several duplicate pairs, such as ClDof3/ClDof23, ClDof13/ClDof15, ClDof14/ClDof16, ClDof13/ClDof20 and ClDof20/ClDof36 (Figs. 2 and 5), suggesting that these motifs might be involved in the functional divergence of ClDofs. The organization of exon–intron structures can provide insights into the evolutionary relationships within certain gene families (Zhou et al., 2018a). In this study, the number of introns of *ClDof* genes varied from 0 to a maximum of 2, and most of them contained one intron or no intron at all (Fig. 4). Similar results were obtained in many other plant species, such as cucumber (Wen et al., 2016), poplar (Wang et al., 2017), eggplant (Wei et al., 2018), pear (Liu et al., 2020), *Arabidopsis* and rice (Lijavetzky, Carbonero & Vicente-Carbajosa, 2003), revealing that the exon–intron structure of *Dof* genes is highly conserved in plants, which may be related to their similar functions.

The specificity of gene expression in plant tissues and developmental stages can provide important information about the possible functions of genes, and previous reports have revealed that some *Dof* genes usually have tissue-specific expression patterns (Ma et al., 2015; Venkatesh & Park, 2015; Zou & Yang, 2019). For example, *ZmDof3* was found to be exclusively expressed in the endosperm of maize kernel and participate in the regulation of starch accumulation and aleurone development in maize endosperm (Qi et al., 2017). Another maize *Dof* gene *ZmDof36* was also reported to be highly expressed in maize endosperm and function in starch synthesis by regulating the expression of starch synthesis genes (Wu et al., 2019). In this study, *ClDof2*, *ClDof5*, *ClDof8*, *ClDof21* and *ClDof35* showed much higher expression in leaves than in other tissues, suggesting that they play essential roles in leaf development. Similarly, seven potato *Dof* genes (*StDof15a*, *StDof22*, *StDof26*, *StDof29a*, *StDof32* and *StDof34*) exhibited higher expression in leaf tissues than in other tissues (Venkatesh & Park, 2015). In addition, *ClDof11*, *ClDof27*, *ClDof29* and *ClDof36* were predominantly expressed in fruits (Fig. 8), suggesting that they may be associated with fruit development of watermelon. In a previous study, a number of *MaDof* genes were markedly regulated throughout the fruit development of banana (Dong, Hu & Xie, 2016), and MaDof23 can act as a transcriptional repressor and interacts with MaERF9 to regulate the fruit ripening by controlling specific ripening-related genes (Feng et al., 2016). Besides fruits, the flowers also showed high expression of *ClDof11*, *ClDof21*, *ClDof27*, *ClDof29*, *ClDof35* and *ClDof36*, which was also observed in other plants. For example, all *PheDof* genes displayed differential expression patterns during the flower development stage of moso bamboo (Cheng et al., 2018; Wang et al., 2016), and overexpression of *PheDof12-1* in *Arabidopsis* resulted in early flowering under long-day conditions (Liu et al., 2019a). In rubber tree, the *HbDof* genes in Cluster III and Cluster VI are typically expressed in male and female flowers, respectively (Zou & Yang, 2019). It should be noted that *ClDof21* and *ClDof27* were segmental duplication genes and they exhibited similar expression patterns in various tissues (Figs. 5 and 8). The qRT-PCR results revealed that both of them were highly expressed in mature leaves and fruits but lowly expressed in tendrils (Fig. 8). Therefore, the tissue-specific expression patterns revealed that *ClDof* genes play vital and seemingly redundant roles in watermelon growth and development.

*Dof* genes are known to play a crucial role in stress responses. For example, tomato *SlCDF1*–*5* genes were differentially up-regulated by osmotic, salt, heat and cold stresses, and transgenic *Arabidopsis* plants overexpressing *SlCDF1* or *SlCDF3* displayed higher drought and salt tolerance (Corrales et al., 2014). Another *Dof* gene *SlDof22* was shown to control the ascorbate accumulation and salt stress in tomato (Cai et al., 2016). In this study, many stress-related *cis*-elements were found in the promoter regions of the *ClDof* genes (Fig. 7), implying their roles in regulating responses to various stresses. In addition, all of the detected *ClDof* genes showed differential expression under salt stress (Fig. 9), suggesting their regulatory roles in salt stress response. It should be noted that *ClDof5* was induced gradually by salt stress (Fig. 9), and had the highest expression in leaves (Fig. 8). Similarly, *GhDof1* also showed the highest expression in leaves as compared with in any other tissues, and salt treatment induced its transcript accumulation. Overexpression of *GhDof1* in cotton resulted in significantly higher salt and cold tolerance (Su et al., 2017). However, the expression levels of *ClDof2*, *ClDof11* and *ClDof21* significantly declined across all time points (Fig. 9), implying that they may play negative regulatory roles in salt stress response. In banana, many *MaDof* genes were found to be down-regulated under drought and salt stress conditions (Dong, Hu & Xie, 2016). In addition, two pairs of segmental duplication genes, *ClDof3/ClDof22* and *ClDof20/ClDof36*, displayed similar expression patterns under salt treatment, whose expression levels decreased at early time points but increased at the late time points (Fig. 9), suggesting their similar roles in response to salt stress. Notably, since the expression of detected *ClDof* genes was altered under salt treatment, *ClDof29* exhibited much higher expression levels than other detected genes (Fig. 9), indicating that *ClDof29* might be the primary regulator of response to salt stress in watermelon. Moreover, a large number of *cis*-elements associated with stress-related hormonal response, such as ABA, GA, SA, MeJA, ethylene and auxin (Fig. 7), and all of the detected *ClDof* genes exhibited ABA-dependent expression patterns (Fig. 10). In castor bean, a large number of *RcDof* genes were regulated (13 up-regulated and 2 down-regulated) in response to ABA treatment (Jin, Chandrasekaran & Liu, 2014). In *Arabidopsis*, the expression of *AtCDF3* was induced by cold, drought, salt, and ABA treatment, and *AtCDF3* overexpression could promote tolerance to drought, cold and osmotic stress (Corrales et al., 2017). ABA is thought to participate in the adaptation of plants to various abiotic stresses by regulating the expression of numerous stress-related genes (Zhou et al., 2019). These results indicate that the *ClDof* genes may play important roles in plant adaptation to salt stress through ABA-dependent pathways.

## Conclusions

In this study, we performed a comprehensive analysis of the phylogenetic relationships, conserved motifs, gene structures, chromosome distributions, and gene duplications of 36 *Dof* genes in watermelon. In addition, qRT-PCR was employed to examine the expression profiles of the *ClDof* genes in different tissues and in responses to salt and ABA treatments. All of the detected *ClDof* genes were regulated by salt and ABA treatments. Our findings may help the functional research of *ClDof* genes for dissecting their roles in the growth, development and stress response of watermelon.

## Supplemental Information

10.7717/peerj.8358/supp-1 Supplemental Information 1 Dof protein sequences used for phylogenetic tree analysis.

10.7717/peerj.8358/supp-2 Supplemental Information 2 Primer sequences used in qRT-PCR.

10.7717/peerj.8358/supp-3 Supplemental Information 3 The amino acid sequences of ClD of members.

10.7717/peerj.8358/supp-4 Supplemental Information 4 The CDS sequences of ClD of members.

10.7717/peerj.8358/supp-5 Supplemental Information 5 The gDNA sequences of ClD of members.

10.7717/peerj.8358/supp-6 Supplemental Information 6 GO analysis of the watermelon Dof family proteins.

10.7717/peerj.8358/supp-7 Supplemental Information 7 The qRT-PCR results of the expression profiles of nine selected ClDof genes in various tissues, under salt and ABA treatment.

10.7717/peerj.8358/supp-8 Supplemental Information 8 GO analysis results for ClD of genes. Three categories, including cellular component, molecular function, and biological process, were identified and visualized with the WEGO program.

## Additional Information and Declarations

### Data Availability

The following information was supplied regarding data availability: Data is available in Figs. 8–10. The qRT-PCR results of the expression profiles of nine selected ClDof genes in various tissues twelve selected *ClDof* genes, under salt and ABA treatment are available as a Supplemental File Table S7.
