# Supplementary material for: Genome-wide characterization and expression analysis of the Dof gene family related to abiotic stress in watermelon
Source: PeerJ. 2020 Feb 17;8:e8358. doi: 10.7717/peerj.8358 (PMC7032062; doi:10.7717/peerj.8358)
Supplement: Supplemental Information 3 [file peerj-08-8358-s003.doc]

**Table S2.** The amino acid sequences of ClDof members.

>ClDof1

QNIQSKSDAFKCPRCHSLHTKFCYYNNYNYSQPRHLCKTCCRYWTLGGLLCNVSIGGGTH

RSKKNSKAKTAIVRDSPPSNSAVLRSDLEMFSIMLKLNQAGDWCGWIENGRELDGGIVGA

ARDSTVEGSGSSDHLATSGGNEGMVEISVASPSMEVPGSNSMN

>ClDof2

MKASLEEEIRQKSSSSGRTKQNNNGDHCEQELGGLKCPRCDSTNTKFCYYNNYSLTQPRH

FCKTCRRYWTKGGALRNVPIGGGCRKTKKLKSSSSSSSSVTTAAPLPDHSSLAFKFFAGI

SPPPPPPSVDFGAIGGEGSCLHLASSIESLSSLNQDLHWKLQQQRLAMIFGDQEQQQQSQ

NKDNQNHNFIQPILFHNLHHQKSAAPSTTTTTISDQWFFPNQVTATNNIASRSVDDGSGW

NSNNNDNHGGIQAWEDLHHFTAFP

>ClDof5

MGLTSLQVCMDSSDWLQGTINEESGMDSSSLSGDMLSCSRPLTERRLRPQHDQALKCPRC

DSTHTKFCYYNNYSLSQPRYFCKTCRRYWTKGGTLRNIPVGGGCRKNKKVSTTKKSNDQQ

QQQQQQQPQIQQPISQNHHHLLHHHHHHHGPSSSSSSSLHIHNPTDLHLSFPDQVQFPHF

NPLIGTTPPNFTIGMLETHHHHHQTRPIDFMDTKMEAIVGNNGHYSTNTDHLAMVGGLNG

DHHITAAATNFHGLCSPYGLSLDGNINQMMIPYDQHQTNEDPNGMDVKPNTKLLALEWQD

QGCSEKVESYGYINGIGSSWNGMMNGYGPSTTNPL

>ClDof9

MGLSSKQVSIDGFDWSKALMQAQKLELPKLAPSSGVKRSQHQNQNQIQPQIEQLKCPRCD

STNTKFCYYNNYNKSQPRHFCRACKRHWTKGGTLRNVPVGGGRKNKRLKKKPTPKSTKSS

SSAAADVINPQMDVHHFQNLPLYQGLIFSPPSSSNWAECENFTTNYGILNSQPPDFSAVS

TTTSTHSPMSPKFNNYSDQELKPTETEQPANSTSTHHPWQLPSTACGVADMSNSYWSWDD

INTFAATDLNIPWDDDHDIKP

>ClDof6

MPSDAGDHRKAATKPLGSGGGVCPPPEQEHLPCPRCDSTNTKFCYYNNYNFSQPRHFCKS

CRRYWTHGGTLRDIPVGGGSRKNAKRSRTCIPSSAAVASSSSGSLPNSISRLDHHSLPAT

PVLVPLVSGHGGGVGGDLKVGGGNMCGSFTSLLNTHAPGFWGLGGFGLGLGSGFEDVGYG

AASPRVAWPFLGLGDGGSGVGGHGGTPNAWQFENGDAAGFVGAAECLSWPELAISTPGNG

L

>ClDof7

MIQELFGVSGLLAAAGDTKISINGSILDSSPSTSLSASATAATTATITTTTTAAASTANA

TSTTSSISEGQNLRCPRCDSSNTKFCYYNNYNLTQPRHFCKTCRRYWTKGGALRNVPIGG

GCRKNKSTAATVAAAVGKSAAGKMKTLSSEILGRVGFRNGSALDHEIISSPKQILWGSPQ

NSHLLAILRSATQNPNPNNLAASHVINNDPPPSTPFHARTMGFDDPVAATHISSLGLCSS

YWRNNQTQVHHQQNGYPHGGGDQVHGGAGGIKELYQKIKSSSNNYFTDGHQGSVGVTSVG

TTTTAAILEAAPVGGGETTGFWNPAFSWSDVHASANGAY

>ClDof3

MGIDYCGFDDQFAGGGVVVKQMEGGGSSSSSKPNNNNNNNNNNGNLLERKARPQKEQALN

CPRCNSTNTKFCYYNNYSLSQPRYFCKACRRYWTEGGSLRNVPVGGGSRKNKRSSLSSSA

DKKINASDQNPKIIHHAHQDLNLAIFPPNNNNNNNTPPIPSSTSTSSSSHLLGSFMTAAP

AAGVFNGGGGFGLSELKPSSLSFSLEGFENGGGYGDLHHNHHHHHHHHHHHQDQTAVMFP

MEELKQSNGRGEFEENRGGGGHGAGDNNSGGGFWNGMLGGGS

>ClDof10

MQYQDLRSKSLQVQDQVQPQPQKCPRCDSLNTKFCYYNNYSLSQPRYLCKACRRYWTQGG

ILRNVPVGGGCRKGKRPKQSSQSSNSIVKSVQPSTSLPPPPQIVPLSLTTTTTEHVIFSG

TPVITPSTFFNPGGELLSSSSWINSFGSSQGQSQIQPPEIIYEMMDQSSGKAATSSSSTV

AGWTESYINNSISNHMAVAGDAIVWPAGNNNAANVTTAAATTTINQWPDYMPGFCPP

>ClDof11

MASTSRIMDKPRQEQQQLQQPPSATLKCPRCDSSNTKFCYYNNYSLSQPRHFCKACKRYW

TRGGTLRNVPVGGGCRKNKRLKTSSSSVSATSTSTPSQNPRLHNSINSSTTPNIISSNHI

NSPMFFGLDPIGSGGLGFSSSGLLSHFHDLHQQPFNSSQFHHPAISFDRNSHVLGNFEPS

LMTPMKEIKIEGLNRLYQNQSEQIDLSNFSDPSSVYWNSGTTATDNWHDPTNNNGSSIAS

L

>ClDof12

MAEVHSSNTDGIKLFGTMIHLQTRKMKEEPEKGGGEGDETEMKRPEKIIPCPRCKSMDTK

FCYFNNYNVNQPRHFCKGCQRYWTAGGALRNVPIGAGRRRTKPPPNCRTLSGELPEDYGQ

LYDAASGIIHQLELDAVEGWRLTLAEQDFSSVFPFKRRKIIGQHGQTS

>ClDof4

MDTAQWPQEIVVKPIEEIVTNTCPKPSVSILERKIRPQKEQALNCPRRYWTEGGSLRNIP

VGGGSRKNKRPSSSSSSTSPTPSSSQPIPKKILDLPQNPKSTQDLNLFFPPSNHQDHHHF

ITNSHPEIVAPAIASSSSASTVATAAVSSSPSAPSHHHPLSAMELLAGMANNNNNNSRGL

NCFFPMSSVVVPDPAGSLYTVGFPDLHDQFKSNLGLFSLDGYSVVPTEGSKGGGRLMQLP

FEDLKQGSNGGVEQGKEQGEHNSSGYWNGMLGGGSW

>ClDof8

MLPEKLAPGGCRPKNSQAKPTSDHNQQALKCPRCDSPNTKFCYYNNYSLTQPRYFCKTCR

RYWTKGGALRNVPVGGGCRKNKKVKSSSLSLPSSKDDSGSSNPEIGRLGFFVNGFSSGQL

GGNRNSSFSTAPTTGLYDQFGVFSLDQSRSLNSFIPSTGFVNSLNVDTSLASSIESLSSM

NQDLHWKLQQQRLAMLFGTGHNNTAIDKNRGALEDHGHELKPCSFQNLEISKPEACNSSD

FSNARNETVAVTGAGVRAGGEPAAADEWFFGDSYTAPSMTMGAAAAGYNSGDSAARCDGV

QEWHDLHPYTHLP

>ClDof34

MTTSTTEDVISAPHFKDLAIKLFGRTIPLPESQISTAPLQNPDACNNLKKAEQSVLGAED

SCPSERSSVLVGDNEENQASNVTLNKGELELPLKEEQADCNGTDQERVFKKPDKIIPCPR

CNSLETKFCYFNNYNVNQPRHFCKNCQRYWTAGGTMRNVPIGAGRRRNKQLASQYRQIIV

SSEGVATTRLETSDTTNHHQLLSGVESPPTLRPSTGNSTVLKFGPEAPLCESMETVLSLG

DQKRSIEIGSAYCGDSPEEPSSCGSSMTTTSIRGNELPKTIVERPEAVRLSNSSSDIAAS

NTVHCYPVPQLVFPLNQGSNGIISSAMAQSSDSTSVANTSSHPNPPVQWLPATMLAVPGF

CTPSLPLQFVPASCWGCTPVWTATGAGNLTVVPSDICASQTPPTAIGSCPTSSSPTLGKH

LRDANSLTEDEKSEKCVVVPKTLRVDNPSEASRSPIWTTFGIRPYPKETISKGSVFETSE

TTNADSKGHFRDAPHILEAKTGSFYSLL

>ClDof33

MSEPKDPAIKLFGKTIPLPEASPATPQLPSSLPILTPGDDNTAAVDHDHDSSSSSLSPEA

NIDGDAEDLEVDKETIRGKSGGTKLEDGDGDGDGDGDGDGDGDGDGGLSVSTEEFTNSDT

SAVRSENSKVLSGEESNPSSTTKTDEQNETSNSQEKTLKKPDKILPCPRCNSMDTKFCYY

NNYNVNQPRHFCKNCQRYWTAGGTMRNVPVGAGRRKNKSSASHHRQIIVSESLQHARTDV

SNGIHHSALKPNGNVLAFGSDAPLCESMASILNIADKTRQNSTRNGFQKPEALKIPVAYE

NGENDDQSQESAATPSFINNEEGKTGPQDQVIHNCQGFLPPQIPFFPGAPWPYPWNSPQW

SSPVPPPTFYPPGIPMPFYPTAPFWGCTVPGAWTVPWVSQPPSLSPVAQNHAPNSPTLGK

HSRDENVTKQSDFGEDEQQKDNKAERCLWIPKTLRIDDPGEAAKSSIWATLGIKNDKTDS

VSEGLFKAFQSKKTNGKNHKAEASPVLQVNPAALSRSIKFHESS

>ClDof35

MVEISPNCPRCGSSNTKFCYYNNYSLTQPRYFCKGCRRYWTKGGSLRNVPVGGGCRKNRR

PPKPLNLTPSPSNPKFHHSNDGNCHRNHRGLSDHDMSPTIDLAAVYANFVNQKADTPETL

PTANTTSNATGHFSVSGESGGVGCGNYCEFSMQECGLGANDYQVAGCFGLEENNNDHQVD

NIEVAARELQLPPLPGEDMLGGWGNPMIMVNNHHRLQPTRVEMFGMETQDPNLLSGNWSP

IDLSNYDTFSRA

>ClDof36

MKKENPEKHHEFFLHSPAYLDPSNWQQQVTHQVGSSSSTGVSSQLLPPPLPPPPPPPPPP

PHGVGGASSIRPGSMAERARMANIPMPEAALKCPRCESTNTKFCYFNNYSLTQPRHFCKT

CRRYWTRGGALRNVPVGGGCRRNKRSKGSSSKSPPVSSDRQQTSGSGNSSSSAIASNNSG

GLSPQIPPLGRFMAPLHQQLSDFDIGGFSYGGGLSAAATATGDLSFQLGNTTLAGGTSIG

SLLGFDQQWRLQQQPPQFPFLSGLDPFDGGSSSGGEGPGPGPGWQMRPKLPSSSRNVSQM

GNSVKMEETPDQVNNAGRQFLGNEQYWSSGSMAWSDLSGFSSSSSTRNP

>ClDof14

MLAISCPKVQQENRKPMRPQPEQALRCPRCDSTNTKFCYYNNYSLSQPRYFCKSCRRYWT

KGGTLRNVPVGGGCRKNKRPSPSTSSSSSPSSKRSHDQAANSSRLMLTDTHLPSLAFEST

DLSLAFAKLQHQSNGQIGLFDTTHNNFHNLYSGFGTGSSGEVENGGFPHFENYNGSGVAS

TATATAMKQELVCNGDQNNMVLLGFPWQFNNNGDGNYMGDLDAGRESWNFNGNGIASSWH

GLLNSPL

>ClDof13

MAHSSLPIFLDPPNWHQPNQPAAAATNDVHQDPRQLPAAFLQPPPPPTAGHGGGGIRPGS

MAYRARLAMIPQPEAALKCPRCDSTNTKFCYFNNYSLSQPRHFCKACRRYWTRGGALRNV

PVGGGFRKNKKKKKSNPSKSPTASQSQMGNSRSIMSRSCNNMESSSTMRDFPSHSSLPSL

NILPSLQQHFSRNFGNNFPGIHINNSAVAREYDVEWQQQQQPFIVAGLESPTTATTTAAA

YTQAEVNNNGNNNNFVANSLQQAAGLVQYNNNCHQQLQNLTPFSRIPMKNEEQNQEQGIL

LSNFLRPNDHNSQFWGSNHQNSWTDHLSALSSSSSSHL

>ClDof16

MMVCQNSKDQIRKPRPQPEQALKCPRCDSTNTKFCYYNNYSLSQPRYFCKSCRRYWTQGG

TLRNVPVGGGCRKNKRSSSTNSSTSSSKKSQDHPFGSGGLPHLSYDHQAHDLSLAFARLH

KNSCGSGSGPAFNDFDFSILGMPNGDVLNGFGYNSQSMYYGNDNNMGGIESNGDQMRLQP

YDQDHHHHHNQQYSNATTTAVTVTTMKQELFGGRDINGGDQSKILWGYPNWQMNNNNNAI

DSNTTTTTTMMAAMDFDSGSARESWNNNAFTNASSWHGLLNSPL

>ClDof15

MDFSSVPIYLDPPPNWHQQSNHHHHHHPQISSNNNGSPQHQHHLLSLPTQQLSSSPLSHV

GGGGGVSSIRRGSMADRARMANVPLPETALKCPRCDSTNTKFCYFNNYSLSQPRHFCKSC

RRYWTRGGALRNVPVGGGCRRNKKNKTRRSKSPAAAPAGNETQVMNNNSNSPTTTTIPLH

SSAENIIGHLQPQHPHLSFMASLNNFSRYGTTSLGLNFNEIQAQGNDIGNGALLNHHLWR

GSQNFPLSGGLETPPGLYPFQISGGDGDDNTTNSILTPNSRATHLPPVKIEETQVLNLLK

SSNLGINNSENNQFWSNGNGWTDLSAISSTSSGHISCDFN

>ClDof17

MGERKNNNEDQYEGGIKLFGATIMLQNNRQIKEEEEEEEEEEANKSDQQSLEKRPEKIIP

CPRCKSMDTKFCYFNNYNVNQPRHFCKGCQRYWTAGGALRNVPVGAGRRKTKPPCRTFAG

LPENCVFDSSGIVAVQPFELEGMVEEWHVVAATATQGGFRQILPVKRRRDCQDGQTC

>ClDof18

MDDNNNNKQLPTSQDQKTQQKQQHQEQLRCPRCDSSNTKFCYYNNYSLSQPRHFCKACKR

YWTRGGTLRNVPVGGGCRKNKRLKRPTTASTTITHSSSSPAPTAPDSTSSSSTSCNTNPL

HNIFYGPTSDLNLPFSGYDHHHHHHLQTHLNAIGLGFSSPNIDHRDFNLNGFNSSSLLSG

YSSLFGNNNNNPSSNSTSISSLLASKFDPNLFGNLANSQPFDDHVMGSNGGDQLGLNVKD

VKLEDGMKRLNWEDDDQQQNHQTDEIVAQSNDHNSLFGNNWASQNWHHDPPNLASSITSS

LI

>ClDof19

MQGAPNEDQTKTSTIIQKQKCPRCESSNTKFCYYNNYSLSQPRYFCKSCRRYWTHGGTLR

NVPIGGGSRKSKRPKTTMPSSSASSDTTTLTPPPPAAGGLPLDHLATSYSSAAFLPSLDV

GAYSFGGLPWTTSITNTIIHSSSSGNNQNSSLDIENNSCTLLPNHLPPYAHPP

>ClDof20

MVFPSIPSNYLDPTTTHQWHQQTASQQSGSSSFQLAGSPAPPQVRPVSMADRARMANLPV

PETALKCPRCESTNTKFCYFNNYSLSQPRHFCKTCRRYWTRGGALRSVPVGGGYRRNTKR

TKSTSKSPVNSQCQPTTTEVSGGEAMRLNYNNAISTQNEGAVDSTHHPIGIANFLGFDQI

QWRPQLQLHQTQQSPLLVNSARLLGNGGIEASPSYSIVRQIQKKMEATTKMEEDGQQVEM

NVAKPFWGVVGGSDHRHYWSGFNPSSSSHS

>ClDof21

MLDSKDPTIKLFGRNIPLSEDGEPPAILSRDFPSQKHAEPIKDDAVDDPEKPVDDSDDSR

NLEREEEASVNPKTPSIDEETATPTNGEQESEKPNSEKTLKKPDKLLPCPRCKSMETKFC

YYNNYNVNQPRHFCKACQRYWTAGGTMRNVPVGAGRRKSKNSASYYRHITISEALEAARI

EAPNGTHKPKFIGNNGRVLSFNLDAPSSDAVVGSVLNLAENRVLSNGVKKFEEKGSEGCD

KSSSLSSMAVQSSSELKINGFPSQISCLSGVPWPFIWNSSVPPPAFGPPGFPLSFFPAAP

WNCGVPGPWNTPWFSPQPEKSVRSDSEASSTLGKHQRDNETAKEDAISSKEEGVKQRNGH

VLTPKTLRIDDPSDAAKSSIWATLGIKNESITGGKNLFKTFHPKGHEKVHVAEASSVLQA

NPAALSRSLVFHES

>ClDof23

MDSAHWPQVEVKSMELEEEGIKAVVERKAKARKDQILNCPRCNSNNTKFCYYNNYSLSQP

RYFCKSCRRYWTAGGSLRNIPVGGASRKNKRPSANFSSPPSKNNQKNCYNDNNNNGDDDD

HQGISQLNINTTCSSTITNTATSCCWLSSDDHMNNQIMLRSSGIMSQRELIPFIPMPAPA

PPQPSTAAALEDFKQLSTIISTDQNGAKLGDVPAFWNGIFGGGSW

>ClDof24

MAEVEIGDPIIKLFGKTIALPLNHLDLPSDSKFLSSETSVLKAGEKETSCEALATEKRGI

SSGNQITDQTTSGMSENPSEEREISSPKASKNEEQSETSISPDNKTSKKPDKILPCPRCN

SMDTKFCYYNNYNVNQPRHFCKNCQRYWTAGGTMRNVPVGAGRRKNKSSSSSHFRQLIIP

DGGIHRHQFLGNNGTFLTFTSDSSISDSQNCNPSGFLISTENGDDHSSKSSITASNSSEK

DGKMISQQSVVKNVLPFQPQLQTFTGLSWPYSSTAPLPYYPPAFPVSYYPALPYWGCTAP

PSWTVINHNGQNSFTNSSVLGKRLRDGKLVRSPANSECEEIVKQRNCEEPCFWNPKTMKV

DDPNEAAKSCIWSTLGIKNEKAGASTINGGSLFVSLQQSAKGKEENHIESCSLLQANPAA

FSRALKFREI

>ClDof22

MEAMMESGSGGGGGGGGLKGKGRPQEQLNCPRCKSSNTKFCYYNNYSLTQPRYFCKSCRR

YWTEGGSLRNIPVGGGSRKNRKPPGPVVGGSGSAHHAPPLPQVYQAHDLNLGFATAEATA

TTMGMENGGHGGFGCYIPNLMPYSARDTAAVEENSGNNGYWNGMFGGGPPW

>ClDof25

MEDINPNSSTRSSPSSSSILEIKKSTKPPKDQLNCPRCKSNNTKFCYYNNYSLTQPRYFC

KSCRRYWTEGGSLRNVPIGGGSRKNRSKSTTTITIMSNSSAPSSSSSSSSDHFNNNNPKL

PNYPASQFSSQNPVGKDLNLAFPCAMSHYADHQLSKVENVNNSNYNCGIGFRGLSSFIPN

LMPNSNNNNTNLGINSNGQYPTGFSLPDLDEFKPSLGINSVDGFGDGRNLLPFQELNQQS

AAPNEGDHQNERQQGNNISTGYWSGMLEDESF

>ClDof26

MQDIHSIAGGSRLFGGGGGGDRRLRPHLHHHQNHQALKCPRCDSLNTKFCYYNNYNLSQP

RHFCKSCRRYWTKGGVLRNVPVGGGCRKTKRSSSSKSKVNSDAAATPPPPPSLRERKSTS

HSSSESSSLTATTTTAAAAAAAATEAVSAPSSNSASTLLNVQDTKLFPGSTTNTNPNFEG

TAAAAISDCGIFSEIGSFTSLITSSNETLAFGFGNMTDVTAFTMNNHQALANQWPLPQRM

MNVNDELKMQEITDGGGGGYMDQTAQVYPSGLQNNRSNNIGFGPLDWQSNGDHQVLFDLP

NAVDQAYWSQNQWSDQDQPNLYLP

>ClDof27

MLEIKDPAIKIFGKEIQLPADCEVSLIESDDSVSTSDKESGDGALQKDVGKATESLAAKD

GTLHDSEDSACVQTANEAHMNPEVVSMDENDKFATSKPEKEQNDAPNSKEKLKKPDKILP

CPRCNSMETKFCYYNNYNVNQPRHFCKACQRYWTEGGTMRNVPVGAGRRKNKNSASHYRQ

ITISEALQAAQIDIPNGVNCLATKSNGRVLNFSVNAPVCESMSTVLNPAGRKVLNGTRNE

FHRLDDQGIKAPCKGGETGDDCSSASSVTMSSSMEEGARRCPQEPQMQNINGFPPQIPYL

PGVPWPCSWNPPMPPPAFCPPGVPLSFYPATYWSCGVPGAWNIPWFPPQPCSPNSGANSP

TLGKHSRDGDKLQADNSENEEPPKQKNGSVLIPKTLRIDDPDEAAKSSIWETLGIKNDSI

KAVDLSKVFQSKGDQKNRVSEVLSPVLQANPAALSRSLTFHERS

>ClDof28

MVFSSSLPSYLDPPNWQPLTNHHTGGNDTAEDSGQVLLPPPPGGGGGSDSGGVGGGTGSI

RPVSMTDRARLAKIPQPEAGLKCPRCESTNTKFCYFNNYNLSQPRHFCKTCRRYWTRGGA

LRNVPVGGGCRRNKRTKSRNRSKSPVAGERQLLGGSANSTAGVSLSTQPHVPFLSSLHNF

SNYGLNLGIIPPQAPPSSGGSTTGGVDVHEFQGDHWRLQQPQQFPLLANDQQPNLLYTFE

PPEGSMTRYNLGGFSRLGIENDMGMTTEAGAVKVEESKGMNLGKNFQFWVGGGGGSDVNA

WSGGGGDLHGFSSSSASHLLRQ

>ClDof29

MQDPSTFQPIKPQFPEQEQLKCPRCDSTNTKFCYYNNYNLSQPRHFCKNCRRYWTKGGAL

RNIPVGGGTRKNSKRAVAATVKRQPSTSSSSSSTLTNPNPITAPDHNPIGIYGGGLDIPG

SFSSLLASNGQFGNLLEGVDPNQSELKMVELGEFSGSGRKSAAEEQSTAVPENYLGVLQN

GDSNCWTGGGSHGWPDLAIFTPGSSYQ

>ClDof30

MSDHHQQLSHPPPRLPPPPPPFASHLERKWKPHLETAPNCPRCASANTKFCYYNNYSLSQ

PRYFCKSCRRYWTKGGSLRNVPVGGGCRKSRRAKSSRSSAVSRPTKPDVSTQITDSSSNG

SDIDLAAVFARFLNSPPCSEPQALTSSPESVDPLENSEIFFEGLSDLLFDEENQGQEEKQ

GIPGNYENLPYGLETELGIDEELWPSHQMQEQLEELDSFSCNYNANHFRVSDQFCQVGDN

WSSFDFAAVNNVEYF

>ClDof31

MPSESANRTNSRPQPQHTMGFPPPPQSDPLPCPRCDSLNTKFCYYNNYNLSQPRHFCKSC

RRYWTHGGTLRDVPVGGGSRKNSKRSRYHNINNTPSSASASSSTSVSSSTSASSSSSVSL

LPTAAALNDINDTIPPPGTFTSLLSSQASGFLALGGYASASGSGHPPAAFDDMGFALGRG

LWGTACTYPEVGDLVGSYAAAGAPETSSSGYNAWQMNATDGGGNGGVVDGDLLGWPDLAI

SMQGKSL

>ClDof32

MVFSSVPVFLDPPNWPSQQPTQLQGSGCETNNAQHLPPPAPPTSGGGGSGPGSIRPGSMT

DRARLAKIPQPEAGLKCPRCESTNTKFCYFNNYSLTQPRHFCKTCRRYWTRGGAMRNVPV

GGGCRRSSKRSSKGGSNRSKSPGGSSTSTSTVSSNSCTTDIISQQLGHHPPPPTTHLPFF

SQNLHHLSDFGNLGLNFEALQIPSLPSSAGGVESSILSSGITDHHQWKIPFFANLHQQNG

MYSNFMADQDHHHHQQGADFSNYQRLSKPLLESGFNTTTTTNHHQLENINNNNNNSMKIL

EETQGINNLSRNFLGIQPNDDQFWNSSTTTTTTPGNIAWSSDQISDHFNSTSSTTHLL
