# Supplementary material for: Genome-wide characterization and expression analysis of the Dof gene family related to abiotic stress in watermelon
Source: PeerJ. 2020 Feb 17;8:e8358. doi: 10.7717/peerj.8358 (PMC7032062; doi:10.7717/peerj.8358)
Supplement: Supplemental Information 4 [file peerj-08-8358-s004.doc]

**Table S3.** The CDS sequences of ClDof members.

>ClDof22

ATGGAGGCGATGATGGAGTCCGGCAGCGGCGGCGGAGGCGGCGGCGGAGG

GTTGAAGGGAAAGGGAAGGCCGCAAGAGCAATTGAACTGCCCAAGGTGCA

AATCAAGCAACACAAAATTCTGTTATTACAATAATTATAGTCTCACACAG

CCGAGGTATTTTTGCAAATCTTGCCGCCGGTATTGGACGGAGGGCGGTTC

TCTTAGGAACATCCCTGTCGGAGGAGGATCCCGGAAGAATAGGAAACCGC

CCGGTCCGGTTGTTGGCGGGTCGGGTTCGGCTCACCACGCGCCGCCGCTG

CCGCAGGTGTATCAAGCCCATGATCTGAATTTGGGGTTTGCGACGGCGGA

GGCCACGGCGACGACGATGGGGATGGAAAACGGTGGCCACGGAGGATTTG

GATGTTACATACCGAATTTGATGCCGTATTCCGCACGTGACACGGCAGCG

GTGGAAGAGAATTCCGGTAATAATGGGTATTGGAATGGGATGTTCGGTGG

AGGGCCGCCGTGGTAG

>ClDof31

ATGCCGTCGGAATCAGCCAACAGAACCAACAGTAGGCCCCAACCCCAACA

CACCATGGGCTTCCCTCCCCCTCCCCAATCCGACCCTCTCCCCTGTCCTC

GCTGCGATTCCCTCAACACCAAGTTCTGTTACTACAACAACTACAACCTC

TCCCAGCCTCGCCACTTCTGCAAATCCTGCCGCCGCTACTGGACTCACGG

CGGCACCCTACGCGACGTTCCTGTCGGCGGCGGCTCCCGCAAGAACTCCA

AACGCTCTCGCTATCACAACATCAACAATACCCCTTCCTCTGCCTCTGCT

TCCTCTTCCACCTCTGTTTCTTCTTCCACCTCTGCTTCCTCTTCCTCCTC

TGTTTCTCTTCTCCCCACCGCCGCCGCCCTCAACGACATCAACGACACCA

TCCCTCCTCCTGGCACTTTTACCTCTTTGCTCAGCTCCCAAGCCTCCGGA

TTCTTAGCTCTTGGCGGTTACGCCTCTGCCTCTGGGTCGGGTCATCCCCC

TGCAGCGTTCGACGATATGGGGTTTGCCCTCGGCAGGGGATTGTGGGGTA

CTGCTTGTACTTACCCTGAAGTTGGGGATCTCGTCGGGAGTTACGCCGCC

GCTGGGGCTCCGGAGACGTCTTCTTCCGGGTACAACGCATGGCAGATGAA

TGCCACCGACGGCGGTGGTAACGGAGGAGTTGTCGATGGTGATTTGCTGG

GTTGGCCGGACCTTGCCATTTCCATGCAAGGCAAGAGCTTGAAGTAA

>ClDof33

ATGTCGGAGCCAAAAGATCCGGCGATCAAGCTCTTTGGCAAGACGATTCC

TCTACCTGAGGCTTCTCCGGCCACTCCTCAGCTTCCATCTTCTTTACCTA

TTCTCACACCCGGTGACGATAACACCGCCGCTGTTGATCATGACCACGAT

TCTTCTTCTTCAAGCTTGTCCCCGGAAGCCAATATCGATGGGGATGCTGA

AGATCTGGAGGTCGATAAGGAGACTATAAGAGGAAAATCAGGTGGAACTA

AGCTGGAAGATGGAGATGGAGATGGAGATGGAGATGGAGATGGAGATGGA

GATGGCGATGGAGATGGAGGCTTATCTGTTTCGACCGAAGAGTTTACTAA

TTCGGATACTTCTGCAGTGAGATCAGAGAATTCTAAAGTACTTTCTGGTG

AGGAAAGTAATCCGTCAAGTACTACAAAGACTGATGAACAGAATGAAACA

AGTAACTCGCAAGAGAAGACTCTCAAGAAACCCGACAAGATACTTCCGTG

CCCCCGCTGTAACAGCATGGATACCAAGTTCTGTTATTATAATAACTACA

ATGTCAATCAACCGCGCCACTTTTGTAAGAACTGTCAAAGGTATTGGACT

GCTGGAGGGACAATGAGGAATGTTCCTGTGGGTGCCGGACGTCGTAAGAA

CAAAAGCTCTGCTTCTCACCATCGTCAGATTATTGTTTCAGAATCGCTTC

AGCATGCTCGGACTGATGTTTCTAATGGTATCCATCATTCTGCCCTCAAA

CCTAATGGCAATGTATTGGCATTTGGTTCAGATGCCCCTCTTTGTGAATC

AATGGCTTCCATTTTGAACATTGCTGATAAAACAAGGCAGAATAGTACTA

GAAATGGTTTTCAAAAACCTGAAGCTCTGAAAATTCCTGTGGCTTATGAG

AATGGTGAAAATGATGATCAGTCTCAGGAATCTGCTGCAACTCCATCATT

TATAAATAATGAAGAAGGCAAAACTGGGCCACAAGATCAAGTGATTCATA

ATTGTCAAGGCTTTTTACCTCCTCAGATTCCATTCTTCCCTGGGGCTCCT

TGGCCTTACCCTTGGAATTCACCACAATGGAGCTCTCCTGTTCCACCTCC

TACTTTCTATCCACCAGGCATCCCTATGCCGTTCTATCCGACCGCCCCGT

TTTGGGGTTGTACCGTTCCCGGCGCCTGGACAGTCCCGTGGGTTTCCCAG

CCACCATCTCTGAGCCCTGTTGCCCAAAACCATGCTCCCAACTCTCCAAC

CTTGGGGAAACATTCAAGAGATGAGAATGTGACCAAGCAAAGCGATTTTG

GGGAAGACGAGCAGCAGAAAGATAACAAAGCTGAGAGATGTCTTTGGATA

CCAAAAACGTTGAGGATTGACGACCCCGGCGAAGCAGCTAAAAGCTCCAT

ATGGGCAACTCTGGGAATAAAAAATGACAAGACTGATTCAGTCAGTGAAG

GGCTTTTCAAGGCTTTTCAATCAAAGAAGACCAACGGAAAGAATCACAAA

GCCGAAGCCTCTCCAGTCTTGCAAGTCAATCCAGCTGCATTGTCCAGATC

AATCAAATTTCATGAAAGCTCGTAA

>ClDof35

ATGGTGGAAATATCACCAAATTGTCCAAGGTGTGGCTCTTCCAACACTAA

GTTTTGTTATTACAACAACTATAGCCTTACCCAACCTCGCTACTTTTGCA

AAGGCTGCCGCCGCTATTGGACCAAAGGTGGTTCCCTTCGTAACGTCCCC

GTCGGTGGTGGCTGTCGGAAGAACCGACGACCTCCCAAGCCCTTAAACTT

AACCCCATCTCCTTCAAACCCTAAATTTCACCATAGCAATGATGGCAATT

GCCATCGCAACCACCGTGGTCTCTCCGACCACGACATGTCGCCGACCATT

GATTTGGCGGCCGTCTATGCCAATTTTGTGAACCAGAAGGCAGACACCCC

CGAGACTCTCCCGACAGCTAATACCACGTCGAACGCAACGGGTCACTTTT

CGGTTTCCGGTGAGAGTGGTGGGGTTGGATGTGGCAATTATTGTGAGTTT

TCCATGCAGGAATGTGGATTGGGGGCCAATGATTATCAAGTGGCCGGTTG

CTTTGGATTAGAGGAGAATAATAATGATCACCAAGTGGATAACATTGAAG

TGGCGGCGCGTGAGCTTCAATTGCCGCCGTTGCCGGGGGAGGACATGCTA

GGAGGTTGGGGAAACCCTATGATAATGGTGAATAATCATCATCGTTTGCA

ACCAACACGAGTGGAAATGTTTGGAATGGAAACTCAAGACCCAAATTTGC

TGAGTGGTAATTGGAGCCCTATTGATTTGTCAAATTATGATACATTCTCA

AGGGCTTAA

>ClDof3

ATGGGAATTGATTATTGTGGATTTGATGATCAGTTTGCAGGTGGTGGAGT

GGTTGTTAAACAAATGGAAGGAGGTGGATCATCAAGTTCTTCAAAGCCTA

ATAACAATAACAACAACAACAATAATAATGGTAATTTGTTGGAGAGAAAA

GCAAGACCTCAAAAGGAGCAAGCTTTGAATTGTCCAAGGTGCAATTCGAC

CAACACAAAATTCTGTTATTACAACAATTACAGCCTGTCTCAACCAAGGT

ATTTTTGCAAAGCTTGTCGGAGGTATTGGACTGAAGGTGGTTCTTTAAGG

AATGTTCCAGTGGGTGGTGGTTCAAGGAAGAACAAAAGATCTTCACTTTC

ATCATCAGCTGATAAGAAGATCAACGCCTCTGATCAAAACCCTAAAATCA

TCCATCATGCTCATCAAGATCTCAATCTTGCAATCTTCCCTCCAAACAAT

AATAATAATAATAATACTCCTCCTATTCCTTCTTCTACTTCTACTTCTTC

TTCTTCTCACCTTTTGGGTTCTTTCATGACGGCGGCACCGGCGGCCGGAG

TGTTCAATGGCGGTGGTGGGTTTGGATTGAGCGAGCTGAAGCCATCAAGT

TTAAGTTTTTCTTTGGAAGGGTTTGAGAATGGTGGAGGGTATGGGGATCT

TCATCATAATCATCATCATCATCATCATCATCATCATCATCATCAAGATC

AGACGGCTGTGATGTTTCCAATGGAAGAATTGAAGCAAAGTAATGGAAGA

GGTGAATTTGAGGAGAATAGGGGAGGAGGAGGGCATGGAGCTGGGGATAA

CAATTCAGGTGGTGGATTTTGGAATGGAATGTTGGGAGGTGGATCATGGT

AA

>ClDof30

ATGTCTGACCACCACCAGCAACTCTCCCACCCGCCGCCTCGTCTCCCTCC

TCCTCCGCCGCCATTCGCCTCCCATCTCGAACGAAAATGGAAGCCCCATC

TCGAAACAGCCCCAAATTGCCCCCGCTGCGCCTCCGCCAACACCAAATTC

TGTTATTACAACAACTACAGCCTCTCCCAACCCAGATACTTTTGTAAATC

CTGCCGCCGCTACTGGACCAAAGGCGGCTCCCTCCGCAACGTCCCCGTCG

GCGGTGGCTGCCGCAAGAGCCGCCGTGCCAAATCCTCAAGATCCTCCGCC

GTCTCCCGTCCTACAAAACCCGACGTTTCTACCCAAATCACCGACTCCTC

CTCCAATGGTTCCGATATCGATCTCGCCGCTGTTTTCGCCCGATTTTTGA

ACAGTCCCCCATGTAGTGAACCCCAGGCGTTGACCAGCTCGCCGGAATCG

GTTGACCCATTGGAGAATTCCGAGATCTTCTTCGAAGGTCTCTCAGATTT

GCTATTCGACGAAGAAAATCAAGGCCAAGAAGAAAAACAGGGGATCCCCG

GAAATTACGAGAATCTTCCATATGGGTTAGAAACAGAGTTGGGCATTGAT

GAAGAACTATGGCCATCACATCAAATGCAAGAACAACTTGAAGAATTGGA

CTCATTTTCTTGCAATTACAATGCGAATCATTTTCGGGTCTCGGATCAAT

TCTGTCAAGTGGGGGATAATTGGAGTTCATTCGACTTTGCAGCCGTAAAT

AATGTCGAATATTTCTAA

>ClDof17

ATGGGAGAGAGAAAGAATAATAATGAAGATCAATACGAAGGTGGAATTAA

GCTGTTTGGGGCAACAATTATGTTGCAAAATAACAGGCAAATCAAAGAAG

AAGAAGAAGAAGAAGAAGAAGAAGAGGCCAATAAATCAGATCAGCAATCA

CTAGAAAAAAGGCCTGAGAAGATCATCCCTTGCCCAAGATGCAAAAGCAT

GGACACCAAATTTTGTTACTTCAATAATTACAATGTTAATCAGCCAAGGC

ATTTCTGTAAGGGCTGCCAAAGGTATTGGACTGCCGGCGGGGCCCTTCGC

AACGTCCCCGTCGGAGCCGGCCGTCGCAAAACCAAGCCCCCTTGTCGGAC

CTTCGCTGGCTTGCCGGAAAATTGTGTCTTTGATTCTTCAGGGATCGTTG

CGGTTCAACCGTTTGAGTTGGAAGGGATGGTCGAGGAGTGGCATGTCGTC

GCTGCCACCGCCACACAAGGCGGTTTTCGACAAATTTTACCGGTAAAGCG

GCGGAGGGATTGCCAAGATGGCCAAACTTGTTGA

>ClDof20

ATGGTATTTCCTTCCATTCCATCTAATTATCTTGATCCAACAACAACCCA

TCAATGGCATCAGCAAACAGCAAGCCAACAATCTGGGAGTAGCAGTTTTC

AGCTGGCGGGGTCACCAGCACCGCCACAAGTTCGGCCGGTGTCTATGGCA

GATCGGGCTCGAATGGCGAACCTGCCGGTGCCGGAGACGGCACTGAAGTG

TCCAAGATGTGAATCAACAAACACAAAGTTTTGTTACTTCAACAACTATA

GCCTCTCTCAGCCCCGCCATTTCTGCAAGACCTGCCGCCGCTACTGGACC

AGAGGTGGCGCTCTCAGAAGCGTCCCGGTCGGCGGCGGCTACCGCCGGAA

TACTAAAAGAACCAAAAGTACCTCTAAATCTCCGGTTAACTCCCAATGTC

AACCTACTACTACTGAGGTTTCAGGAGGAGAAGCAATGAGATTAAATTAC

AATAATGCCATTTCCACACAAAATGAAGGAGCTGTGGATTCAACTCATCA

TCCCATTGGGATAGCCAACTTTTTAGGGTTTGATCAAATTCAATGGCGAC

CACAGCTTCAACTTCACCAAACACAACAGTCTCCATTGCTTGTAAATTCA

GCTCGGTTGCTTGGAAATGGTGGCATCGAGGCATCGCCGAGCTATAGCAT

CGTTAGACAGATTCAAAAGAAGATGGAAGCGACGACGAAAATGGAGGAAG

ATGGGCAACAAGTAGAGATGAATGTTGCAAAGCCATTTTGGGGAGTTGTT

GGAGGAAGTGATCATCGCCATTATTGGAGTGGTTTTAATCCTTCATCTTC

TTCCCATTCATAA

>ClDof32

ATGGTTTTCTCTTCAGTTCCTGTGTTTCTTGATCCACCCAATTGGCCTTC

TCAGCAACCAACTCAGCTACAAGGAAGTGGCTGTGAGACAAATAATGCTC

AACACCTTCCACCTCCGGCACCGCCAACTAGCGGTGGCGGCGGTAGTGGA

CCTGGCTCGATTAGACCAGGGTCAATGACGGATCGAGCTAGGTTGGCGAA

GATACCACAACCGGAAGCCGGGTTAAAATGTCCTAGATGCGAATCAACAA

ACACAAAGTTTTGTTACTTCAACAACTACAGTCTCACTCAACCTCGCCAC

TTTTGCAAGACGTGCCGACGGTACTGGACGAGAGGTGGAGCGATGAGGAA

CGTACCGGTTGGAGGCGGGTGTCGGCGGTCGAGCAAGAGATCAAGCAAAG

GAGGAAGCAATAGGTCAAAATCTCCAGGGGGTTCAAGCACTTCAACAAGC

ACAGTGTCATCAAATAGTTGCACAACAGACATAATTAGCCAACAACTGGG

TCATCATCCTCCTCCTCCAACAACCCATTTGCCCTTTTTTTCTCAAAATT

TACATCATCTTAGTGATTTTGGGAACCTTGGTTTAAACTTTGAAGCCCTT

CAAATTCCTTCACTCCCATCATCAGCTGGAGGAGTTGAATCCTCAATTTT

ATCAAGTGGAATTACAGATCATCATCAATGGAAAATCCCCTTTTTTGCAA

ATTTACATCAACAAAATGGGATGTATTCAAATTTCATGGCTGATCAAGAT

CATCATCATCATCAACAAGGAGCCGATTTTTCAAATTACCAGCGGTTGTC

AAAGCCATTATTGGAGAGTGGATTCAATACTACTACTACTACAAATCATC

ATCAATTGGAGAATATTAATAATAATAATAATAATAGCATGAAAATCTTG

GAGGAGACACAAGGGATTAACAATTTGTCAAGAAATTTTCTTGGAATTCA

ACCAAATGATGATCAATTCTGGAATTCTAGTACTACAACAACAACAACAC

CTGGGAATATTGCATGGAGTAGTGATCAAATTTCAGATCATTTCAATTCA

ACTTCTTCCACTACTCATCTCTTGTAA

>ClDof18

ATGGACGACAACAACAACAACAAACAATTACCTACATCACAAGATCAAAA

GACTCAACAAAAACAACAACATCAAGAACAACTCAGATGTCCTCGTTGTG

ATTCATCAAACACCAAATTTTGTTACTACAACAACTACAGCTTGTCTCAG

CCTCGCCACTTTTGCAAAGCTTGTAAGCGTTACTGGACACGTGGTGGCAC

GTTGAGGAACGTTCCCGTCGGCGGTGGTTGCCGGAAAAACAAGCGTCTCA

AAAGACCTACAACTGCCTCCACCACCATCACGCATTCGTCATCGTCCCCG

GCTCCAACCGCCCCAGATTCGACATCTTCATCTTCAACTTCTTGTAACAC

TAACCCTCTTCATAATATCTTTTATGGCCCCACCTCTGATCTGAACCTTC

CATTTTCCGGGTATGATCATCATCATCATCATCATCTTCAAACTCACTTA

AATGCTATTGGTTTAGGGTTTTCTTCTCCTAATATTGATCATAGGGACTT

TAATCTTAATGGGTTCAATTCAAGTTCACTCCTTTCAGGTTATTCTAGTT

TATTTGGTAATAACAACAATAACCCATCTTCTAATTCAACCTCTATATCT

TCATTGCTTGCTTCTAAGTTTGATCCAAATCTTTTTGGAAACCTAGCTAA

TTCTCAGCCGTTTGATGATCATGTGATGGGATCTAACGGTGGAGATCAAC

TAGGGTTGAATGTGAAGGATGTGAAATTGGAAGATGGGATGAAGAGGTTA

AATTGGGAGGATGATGATCAACAACAAAATCATCAGACAGATGAAATTGT

TGCTCAATCTAATGATCATAATTCTCTTTTTGGTAATAATTGGGCCTCTC

AAAATTGGCATCATGATCCACCAAATCTTGCTTCTTCTATCACTTCTTCC

CTCATCTAG

>ClDof12

ATGGCTGAGGTTCATAGTAGCAACACGGATGGAATTAAGCTTTTTGGCAC

TATGATTCATTTACAGACAAGAAAAATGAAGGAGGAACCGGAGAAAGGCG

GCGGTGAAGGTGATGAAACAGAGATGAAGAGGCCAGAAAAAATTATTCCA

TGCCCAAGATGTAAGAGTATGGACACCAAATTCTGTTACTTCAACAATTA

CAATGTTAATCAGCCTAGACATTTCTGCAAGGGTTGCCAAAGGTACTGGA

CGGCCGGTGGGGCGCTCCGGAACGTACCCATCGGAGCTGGCCGCCGGAGA

ACCAAGCCGCCGCCTAATTGCCGGACCCTTTCCGGCGAGCTGCCAGAGGA

TTATGGGCAGCTTTACGATGCGGCTTCTGGGATTATTCATCAGCTTGAAT

TAGATGCGGTGGAAGGGTGGCGTCTCACGTTGGCGGAACAGGATTTTAGC

TCGGTTTTTCCTTTCAAACGCCGGAAGATTATCGGTCAACACGGTCAAAC

GTCTTAA

>ClDof29

ATGCAAGACCCATCAACGTTTCAGCCGATTAAACCCCAATTTCCCGAACA

AGAGCAACTTAAATGTCCTCGTTGTGATTCTACGAACACCAAATTTTGCT

ATTACAATAACTATAATCTTTCTCAGCCTCGTCACTTTTGTAAGAATTGC

CGTCGGTACTGGACTAAAGGCGGCGCTCTTCGCAACATCCCCGTCGGCGG

TGGCACTCGGAAAAACTCCAAGCGTGCGGTGGCCGCCACCGTCAAACGCC

AGCCGTCTACCTCGTCTTCGTCTTCTTCAACCTTGACGAATCCAAACCCG

ATCACGGCGCCGGATCATAACCCGATCGGGATTTACGGCGGTGGGCTGGA

TATTCCAGGGAGCTTCAGTTCGCTGCTGGCGTCAAATGGGCAATTTGGGA

ATCTTTTGGAGGGTGTTGATCCGAATCAATCGGAGCTGAAAATGGTGGAA

TTGGGTGAATTCTCTGGGTCTGGCAGGAAATCTGCAGCGGAGGAACAGAG

CACCGCCGTGCCGGAGAATTATTTGGGTGTTCTGCAAAATGGGGATTCTA

ATTGTTGGACCGGTGGCGGGAGTCATGGGTGGCCCGATCTTGCTATTTTC

ACACCAGGGTCAAGTTATCAGTAA

>ClDof34

ATGACGACTTCTACTACAGAGGATGTCATCTCCGCCCCACATTTCAAGGA

TCTCGCCATTAAACTCTTCGGCCGGACCATCCCTTTGCCGGAATCCCAGA

TTTCCACCGCCCCTCTTCAAAATCCGGATGCTTGCAACAATTTGAAAAAA

GCTGAGCAGTCTGTCTTGGGTGCAGAAGACTCTTGCCCATCTGAGAGGTC

TTCGGTTTTGGTAGGGGATAATGAAGAAAACCAAGCTTCGAACGTGACGT

TGAACAAGGGAGAATTGGAACTGCCTTTGAAAGAGGAGCAGGCGGACTGT

AATGGCACGGATCAAGAAAGAGTTTTTAAGAAGCCAGACAAAATCATTCC

TTGTCCTAGATGTAATAGTTTGGAAACAAAATTCTGTTACTTCAACAACT

ACAATGTCAATCAACCTAGGCATTTCTGCAAGAACTGCCAAAGATATTGG

ACTGCTGGTGGGACAATGAGAAACGTTCCTATCGGTGCTGGACGGCGAAG

GAACAAGCAGTTGGCATCTCAATACCGACAGATAATAGTATCCTCTGAGG

GAGTTGCGACAACCCGGTTGGAAACTTCGGATACGACGAATCACCATCAA

CTACTCTCAGGCGTAGAGTCTCCTCCTACTTTGAGGCCTTCCACTGGAAA

TAGTACTGTCCTAAAGTTTGGACCCGAAGCACCACTTTGCGAATCTATGG

AGACTGTGCTTAGTCTTGGAGATCAGAAGAGATCCATTGAGATTGGCTCT

GCATATTGTGGAGACAGTCCTGAAGAACCATCCTCGTGCGGATCGTCCAT

GACAACTACTAGTATTCGTGGAAACGAATTGCCTAAAACCATTGTTGAGA

GGCCAGAGGCAGTCAGATTATCCAACTCTAGTAGCGATATCGCTGCATCG

AATACAGTTCATTGTTATCCGGTTCCACAATTGGTTTTTCCATTGAATCA

GGGCAGCAACGGGATAATTTCATCAGCAATGGCACAAAGTTCAGATAGCA

CAAGCGTAGCAAATACTAGCAGTCACCCCAATCCACCAGTTCAATGGCTC

CCCGCAACAATGCTGGCAGTCCCAGGCTTTTGCACTCCAAGCCTTCCATT

ACAATTTGTGCCAGCTTCGTGTTGGGGTTGTACGCCCGTATGGACCGCTA

CTGGTGCCGGGAATTTAACCGTTGTTCCTTCTGATATCTGTGCATCTCAA

ACACCCCCAACCGCCATTGGTAGCTGCCCTACAAGTTCGTCGCCTACACT

AGGGAAGCATTTGAGAGATGCAAACAGCTTAACAGAGGACGAGAAGTCAG

AGAAATGCGTCGTGGTTCCAAAGACATTACGAGTCGATAACCCAAGTGAG

GCTTCAAGGAGTCCTATATGGACGACATTTGGAATTCGTCCATATCCAAA

GGAAACCATTTCTAAAGGTAGTGTTTTTGAAACTTCAGAAACTACTAATG

CAGATAGCAAAGGCCATTTTAGGGATGCTCCTCATATCTTGGAAGCAAAA

ACTGGGAGCTTTTACTCTCTTTTATAG

>ClDof26

ATGCAAGACATACACTCGATCGCCGGAGGAAGTCGCTTGTTCGGCGGCGG

TGGCGGCGGAGACCGTCGTCTACGGCCACACCTCCACCACCATCAAAACC

ACCAAGCCTTGAAGTGTCCGAGGTGTGATTCTCTCAATACGAAGTTTTGT

TATTATAATAATTATAATCTCTCTCAGCCACGTCACTTCTGCAAGAGCTG

CCGTCGTTACTGGACGAAAGGCGGTGTTCTCCGTAACGTCCCCGTCGGCG

GTGGCTGCCGGAAAACCAAGCGCTCTTCCTCCTCTAAGTCTAAGGTCAAC

TCAGACGCAGCGGCGACTCCTCCTCCACCTCCTTCCCTCCGTGAACGCAA

ATCCACTTCTCATTCCAGTAGCGAGAGTTCCAGTTTAACCGCCACAACTA

CTACCGCTGCTGCCGCAGCGGCGGCAGCGACTGAGGCCGTGTCGGCTCCG

TCGTCTAATTCGGCCTCGACTTTATTGAACGTACAGGATACGAAATTGTT

CCCTGGTTCGACTACAAATACAAACCCTAATTTCGAAGGTACGGCGGCGG

CGGCGATTTCGGACTGCGGAATTTTCTCGGAAATCGGTAGCTTCACGAGC

TTGATCACATCGTCTAACGAGACATTGGCGTTTGGATTCGGTAACATGAC

GGACGTGACGGCGTTTACGATGAATAATCATCAGGCGTTGGCGAATCAAT

GGCCGCTGCCGCAGAGGATGATGAACGTTAACGATGAACTGAAGATGCAG

GAGATCACAGACGGCGGAGGAGGAGGGTACATGGATCAGACGGCTCAGGT

GTATCCATCAGGGCTTCAGAATAACAGATCAAACAACATTGGATTTGGTC

CGTTGGATTGGCAATCAAACGGAGATCATCAAGTTCTGTTTGATCTCCCC

AACGCCGTAGATCAAGCGTACTGGAGTCAAAATCAATGGAGCGATCAAGA

CCAGCCTAATCTGTACCTTCCGTAA

>ClDof1

CAAAACATTCAATCCAAATCTGATGCATTCAAATGCCCTAGATGCCATTC

TCTCCACACCAAATTTTGCTATTACAATAATTACAATTACTCTCAACCTC

GCCACCTTTGCAAGACCTGTTGCCGCTACTGGACCCTCGGCGGCCTCCTC

TGCAACGTCTCTATTGGTGGCGGAACTCACAGATCGAAGAAGAATTCCAA

AGCCAAGACTGCGATTGTTAGAGATTCGCCGCCTTCGAACTCTGCTGTTC

TTCGTTCTGATTTAGAAATGTTTTCGATTATGTTGAAGTTGAATCAGGCG

GGTGATTGGTGCGGTTGGATTGAGAATGGAAGGGAATTAGATGGTGGGAT

TGTGGGTGCGGCAAGGGATTCGACGGTGGAGGGATCTGGATCGTCGGATC

ATCTGGCGACAAGTGGTGGAAATGAAGGAATGGTTGAGATTTCTGTGGCG

TCACCGTCGATGGAGGTTCCTGGTTCAAATTCTATGAATTAA

>ClDof4

ATGGATACTGCTCAATGGCCACAGGAGATTGTGGTGAAGCCAATTGAAGA

AATTGTGACAAACACATGCCCAAAACCAAGTGTTTCAATTTTAGAGAGGA

AGATTAGACCTCAAAAGGAACAAGCTTTGAATTGTCCAAGAAGGTATTGG

ACTGAAGGTGGCTCCTTAAGAAACATTCCTGTTGGTGGAGGCTCAAGGAA

GAACAAAAGACCTTCTTCTTCATCATCATCTACATCTCCCACACCTTCTT

CATCTCAACCTATTCCCAAGAAAATTCTTGATCTCCCTCAAAACCCTAAA

TCCACCCAAGATCTCAACTTGTTCTTCCCACCCTCGAACCATCAAGATCA

TCATCATTTCATCACCAATAGCCACCCCGAAATCGTTGCCCCTGCGATTG

CCAGTTCCTCATCAGCCTCTACTGTTGCGACGGCGGCGGTGTCGTCGTCG

CCTTCGGCCCCATCGCATCATCATCCGCTTTCGGCAATGGAGCTTCTCGC

CGGGATGGCGAACAACAACAACAACAATTCTAGGGGTTTGAATTGTTTCT

TTCCAATGTCGTCTGTTGTTGTCCCTGATCCAGCTGGCTCCCTTTACACG

GTAGGGTTTCCCGATCTCCATGATCAGTTCAAGTCAAACCTCGGATTATT

CTCTCTTGATGGCTACAGCGTTGTCCCGACCGAGGGAAGCAAGGGTGGGG

GGAGGCTTATGCAGCTGCCATTTGAGGATTTGAAGCAAGGGTCTAATGGA

GGAGTTGAACAAGGCAAAGAGCAAGGAGAGCATAATTCAAGTGGGTATTG

GAATGGAATGTTGGGTGGAGGATCATGGTAA

>ClDof2

ATGAAGGCCTCATTGGAAGAAGAAATAAGGCAAAAATCATCATCAAGTGG

AAGAACAAAACAAAATAATAATGGAGATCATTGTGAGCAAGAATTAGGAG

GTCTGAAATGCCCTCGATGTGATTCCACCAACACCAAATTCTGTTATTAC

AATAACTACAGTCTCACACAGCCTCGTCATTTTTGCAAGACTTGCCGGAG

ATATTGGACTAAAGGTGGAGCTTTGAGAAATGTTCCAATCGGTGGTGGTT

GCCGGAAAACCAAAAAGCTCAAATCTTCTTCTTCTTCTTCCTCCTCCGTC

ACCACCGCGGCTCCACTGCCTGATCACTCCTCTCTTGCCTTCAAATTCTT

CGCCGGAATCTCACCGCCTCCGCCGCCACCGTCTGTTGATTTTGGTGCCA

TTGGAGGAGAAGGTTCTTGTTTGCATCTTGCATCTTCAATTGAATCTTTG

AGCTCCTTAAATCAAGACCTTCATTGGAAATTACAACAACAACGTTTGGC

TATGATTTTTGGAGACCAAGAACAACAACAACAATCACAAAACAAGGATA

ATCAAAATCACAACTTTATTCAACCCATTTTGTTTCACAATCTACATCAT

CAAAAGTCAGCTGCACCATCTACCACAACAACTACTATTAGTGATCAATG

GTTTTTTCCAAATCAAGTGACTGCCACAAACAACATAGCTTCAAGATCAG

TTGATGATGGGAGTGGGTGGAATAGTAATAACAATGATAATCATGGAGGA

ATTCAAGCTTGGGAAGATTTGCATCATTTCACTGCCTTCCCCTAA

>ClDof7

ATGATCCAAGAACTATTTGGTGTATCAGGGCTTTTAGCTGCTGCTGGAGA

CACCAAAATCTCCATCAATGGTTCAATTCTAGATTCTTCCCCTTCCACTT

CCCTTTCTGCCTCTGCCACCGCCGCCACCACTGCCACAATCACCACCACC

ACCACCGCCGCCGCAAGCACTGCCAACGCCACATCAACAACCTCCTCAAT

TTCTGAAGGCCAAAACCTGCGTTGCCCCAGATGTGATTCTTCAAACACTA

AATTCTGTTACTACAACAACTACAACCTCACTCAACCTCGTCACTTCTGT

AAGACATGTCGCCGTTATTGGACCAAAGGTGGGGCCCTCAGGAACGTTCC

CATCGGTGGTGGGTGTCGGAAGAACAAGAGCACCGCCGCGACGGTGGCTG

CTGCTGTAGGGAAGTCTGCAGCTGGAAAAATGAAAACTTTATCGTCTGAG

ATATTGGGTCGGGTCGGATTCCGAAATGGGTCTGCGTTGGATCATGAAAT

AATATCATCGCCCAAGCAAATTCTGTGGGGGTCGCCGCAAAATTCTCACC

TTTTGGCTATTTTGAGATCCGCCACCCAAAACCCTAACCCTAATAATCTC

GCCGCTTCCCACGTCATTAACAACGACCCACCGCCGTCCACGCCGTTTCA

TGCTCGGACGATGGGTTTCGATGATCCGGTGGCAGCCACTCATATTTCTT

CTCTGGGGCTCTGCAGCTCTTACTGGAGAAACAATCAGACTCAAGTTCAT

CATCAACAAAATGGGTATCCTCACGGCGGCGGAGATCAGGTCCACGGCGG

CGCCGGTGGGATTAAGGAGCTCTATCAGAAGATTAAATCGTCGTCGAACA

ATTATTTCACCGACGGCCATCAGGGATCGGTGGGAGTAACGAGTGTGGGT

ACAACGACTACGGCGGCGATTTTGGAAGCGGCTCCGGTGGGCGGCGGCGA

AACGACAGGTTTCTGGAATCCAGCTTTTTCTTGGTCTGATGTTCATGCTA

GTGCTAATGGAGCATATCCTTAA

>ClDof10

ATGCAATATCAGGATCTGAGATCGAAGAGTTTACAAGTGCAAGATCAAGT

TCAACCACAACCTCAAAAATGTCCTCGTTGTGATTCTCTCAATACTAAGT

TTTGTTATTACAATAATTACAGTTTGTCTCAGCCTCGTTATCTTTGCAAG

GCCTGTAGAAGGTACTGGACTCAAGGTGGCATTCTCCGAAACGTCCCCGT

CGGCGGCGGTTGTCGGAAGGGTAAACGACCGAAGCAATCTTCACAATCAT

CCAATAGTATTGTAAAGTCAGTACAACCGTCAACGTCGCTGCCACCACCA

CCGCAGATAGTCCCGTTGAGTTTAACAACAACAACTACAGAACACGTAAT

CTTTTCGGGTACTCCTGTTATCACTCCGTCAACGTTTTTCAATCCGGGTG

GGGAATTGTTGTCATCTTCAAGCTGGATTAATTCATTTGGATCATCACAA

GGGCAGTCTCAAATTCAACCACCAGAAATAATCTATGAAATGATGGATCA

GTCGTCGGGAAAGGCCGCCACGAGTTCATCTTCTACAGTGGCGGGATGGA

CAGAAAGCTACATCAACAATTCCATTTCTAACCATATGGCCGTCGCCGGA

GATGCTATTGTCTGGCCAGCTGGCAATAATAACGCTGCCAACGTCACCAC

CGCTGCCGCCACCACCACAATTAACCAGTGGCCTGATTATATGCCAGGAT

TTTGTCCTCCTCCATGA

>ClDof28

ATGGTTTTTTCTTCTTCTCTCCCTTCTTATTTGGATCCTCCTAACTGGCA

ACCGTTGACGAATCACCACACGGGAGGAAATGATACTGCAGAGGATTCTG

GTCAAGTTTTACTACCTCCACCACCTGGGGGTGGGGGAGGTAGTGACAGT

GGTGGTGTTGGAGGTGGTACTGGGTCGATTAGGCCGGTTTCGATGACTGA

TCGAGCCCGGTTGGCGAAGATTCCGCAGCCAGAGGCCGGCTTGAAATGCC

CTAGATGTGAATCTACAAATACCAAATTTTGTTACTTCAATAACTATAAT

CTTTCTCAGCCTCGCCACTTTTGTAAGACTTGTCGTCGGTATTGGACACG

TGGTGGTGCACTGAGGAATGTTCCGGTCGGTGGTGGTTGCCGGAGGAACA

AGAGGACCAAATCCCGGAATCGGTCTAAGTCTCCGGTGGCCGGTGAACGG

CAATTATTAGGTGGATCAGCTAACTCTACTGCTGGGGTTTCACTTTCAAC

ACAACCCCATGTGCCCTTTTTGTCTTCTTTACATAATTTCTCTAATTATG

GCCTCAATTTGGGCATAATTCCGCCGCAAGCTCCTCCATCAAGCGGTGGC

TCCACCACCGGAGGAGTGGATGTTCATGAATTTCAAGGTGACCATTGGAG

ATTACAACAACCACAACAATTCCCTCTCTTGGCTAATGATCAACAACCTA

ATTTGCTTTACACTTTCGAGCCACCGGAAGGCAGCATGACTCGGTATAAT

CTTGGCGGGTTCTCTAGATTAGGGATTGAAAACGACATGGGAATGACGAC

GGAGGCTGGGGCGGTGAAGGTGGAGGAGAGTAAAGGAATGAACTTAGGGA

AGAATTTTCAATTTTGGGTCGGTGGTGGTGGCGGAAGCGACGTTAATGCA

TGGAGCGGCGGTGGCGGCGATCTTCATGGCTTCTCTTCATCCTCCGCCAG

CCACTTATTGAGACAGTAA

>ClDof15

ATGGATTTCTCTTCTGTTCCAATCTATTTAGATCCTCCTCCCAATTGGCA

CCAGCAATCAAATCATCATCATCACCACCATCCTCAGATAAGTAGTAATA

ATAATGGATCTCCTCAACACCAACACCACCTTCTTTCATTGCCTACTCAG

CAACTCTCCTCCTCACCGTTGTCTCATGTCGGTGGTGGCGGTGGTGTTAG

CTCAATCCGGCGTGGTTCGATGGCGGACCGAGCTCGGATGGCTAACGTAC

CACTACCGGAGACAGCACTCAAGTGTCCTCGATGTGATTCAACCAACACG

AAGTTTTGTTACTTTAATAACTATAGTCTCTCTCAGCCTCGCCACTTTTG

TAAGAGCTGCCGCCGATATTGGACTCGTGGGGGTGCCCTAAGGAACGTTC

CAGTTGGGGGCGGTTGTCGCCGGAATAAGAAGAATAAAACTAGACGCTCG

AAGTCTCCGGCTGCAGCTCCGGCCGGAAATGAAACTCAAGTGATGAATAA

CAATTCAAACTCACCCACCACTACTACAATACCCTTGCACAGTAGTGCTG

AGAATATTATTGGTCATTTACAGCCTCAACATCCTCACCTTTCATTCATG

GCTTCATTGAATAATTTTTCTCGATACGGGACAACGAGTTTAGGGTTGAA

TTTCAACGAGATCCAGGCTCAAGGTAACGATATCGGCAACGGCGCCCTTC

TGAATCATCATCTGTGGCGTGGTTCCCAAAACTTTCCTCTCTCAGGTGGC

CTAGAAACTCCACCCGGGTTGTACCCGTTTCAAATTAGCGGCGGGGACGG

CGACGACAACACAACAAATTCAATCCTAACACCAAATTCAAGGGCAACCC

ATTTGCCTCCAGTGAAGATTGAAGAAACCCAAGTGCTGAATTTGTTGAAA

TCATCCAATTTGGGTATCAATAATTCTGAAAACAATCAATTTTGGAGCAA

TGGAAATGGTTGGACAGATCTTTCAGCTATCAGTTCTACTTCAAGTGGCC

ATATTTCTTGTGACTTCAATCATTAG

>ClDof21

ATGTTGGATTCTAAAGATCCTACTATTAAGCTTTTTGGCCGTAATATTCC

ACTTTCCGAAGACGGGGAACCTCCGGCGATTCTTTCCCGCGACTTCCCTT

CTCAGAAACATGCAGAACCAATAAAGGATGATGCTGTTGATGATCCTGAG

AAACCTGTTGATGATTCAGATGATTCAAGAAATCTAGAGAGAGAGGAAGA

GGCTAGCGTGAATCCCAAAACGCCATCTATAGATGAAGAAACTGCGACGC

CTACTAATGGAGAACAAGAGAGTGAGAAACCTAATTCAGAGAAAACCCTA

AAGAAGCCTGATAAATTGCTTCCATGTCCTCGTTGCAAAAGTATGGAGAC

AAAGTTCTGTTACTACAACAATTACAATGTAAATCAACCTCGCCATTTTT

GTAAAGCTTGTCAAAGATACTGGACCGCCGGTGGGACTATGAGGAACGTA

CCGGTAGGAGCTGGTCGTCGGAAGAGCAAAAACTCTGCTTCTTATTACCG

TCACATCACGATCTCTGAGGCTCTTGAAGCTGCTCGAATTGAGGCTCCAA

ATGGAACCCACAAACCAAAATTCATTGGCAACAATGGCAGAGTTCTCAGT

TTCAATTTGGATGCACCGAGTTCTGATGCTGTGGTGGGCTCTGTTTTAAA

CCTTGCAGAAAACAGAGTTTTGAGTAATGGGGTAAAGAAGTTTGAAGAGA

AAGGATCTGAAGGATGTGATAAAAGCTCGAGTTTGTCTTCTATGGCAGTT

CAAAGTTCATCAGAATTGAAAATCAATGGCTTCCCTTCTCAGATTTCATG

TCTTTCTGGAGTTCCATGGCCTTTTATTTGGAATTCATCAGTTCCACCAC

CAGCTTTTGGCCCTCCAGGATTTCCCTTGTCTTTTTTTCCAGCAGCTCCT

TGGAACTGTGGGGTTCCAGGACCATGGAACACTCCATGGTTTTCACCACA

ACCTGAAAAATCTGTGCGTTCTGACTCTGAAGCTTCTTCAACACTGGGAA

AGCATCAAAGAGACAATGAAACGGCCAAGGAAGATGCCATTTCAAGTAAA

GAAGAGGGTGTTAAGCAGAGAAATGGACATGTCTTAACCCCAAAGACTTT

AAGGATTGATGACCCAAGTGACGCTGCAAAAAGTTCCATATGGGCAACAC

TTGGGATAAAGAATGAATCAATCACTGGAGGAAAAAATCTGTTCAAAACC

TTTCATCCCAAAGGCCATGAGAAGGTTCATGTTGCTGAAGCCTCATCAGT

TTTGCAGGCAAATCCTGCAGCCTTGTCGAGATCTCTCGTCTTTCACGAGA

GCTCTTGA

>ClDof5

ATGGGTCTTACTTCTCTTCAAGTCTGCATGGACTCTTCTGATTGGCTACA

GGGTACTATAAACGAGGAGTCAGGGATGGATTCTTCTTCATTATCAGGAG

ATATGCTTTCATGTTCAAGGCCATTAACAGAGAGGAGACTAAGGCCACAA

CATGATCAAGCTCTGAAATGTCCAAGATGTGATTCAACTCACACCAAATT

TTGTTATTACAACAATTATAGTCTTTCCCAGCCTCGTTACTTTTGCAAAA

CTTGTCGTAGGTATTGGACTAAAGGAGGAACTCTAAGAAACATTCCAGTT

GGTGGTGGCTGTAGAAAGAACAAGAAAGTTTCCACTACTAAAAAATCCAA

TGATCAACAACAACAACAACAACAACAACAACCACAAATTCAACAACCCA

TTTCTCAAAATCATCATCATCTTCTTCATCATCATCATCATCATCATGGC

CCTTCTTCTTCTTCTTCTTCTTCTCTTCACATTCACAATCCAACTGATCT

CCATCTCTCATTTCCAGACCAAGTTCAATTCCCTCATTTCAATCCTCTCA

TTGGAACAACTCCTCCAAATTTCACCATTGGAATGCTTGAAACTCATCAC

CATCATCATCAAACTAGACCCATTGATTTCATGGACACTAAAATGGAAGC

CATTGTTGGGAATAATGGTCATTATTCCACAAACACTGATCATTTAGCTA

TGGTTGGAGGGTTAAATGGAGATCATCATATCACAGCTGCTGCAACAAAT

TTCCATGGTCTTTGTTCTCCTTATGGACTATCACTTGATGGGAATATTAA

CCAAATGATGATTCCTTATGATCAACATCAAACAAATGAAGATCCAAATG

GAATGGATGTGAAACCAAATACAAAGCTTTTGGCTTTGGAATGGCAAGAT

CAAGGATGTTCAGAGAAAGTTGAATCTTATGGATATATAAATGGAATTGG

GTCTTCTTGGAATGGGATGATGAATGGATATGGACCATCAACAACCAACC

CATTGGTGTAA

>ClDof16

ATGATGGTATGTCAAAATTCAAAAGATCAAATCAGAAAGCCAAGGCCACA

GCCAGAACAAGCCTTGAAATGTCCAAGATGTGACTCAACAAACACCAAAT

TTTGTTACTACAACAACTACAGCCTCTCTCAACCAAGATACTTTTGCAAA

TCCTGCCGCCGTTACTGGACACAAGGCGGCACTCTCCGCAATGTTCCAGT

AGGCGGAGGTTGTCGTAAAAACAAAAGATCATCCTCCACGAATTCATCAA

CCTCTTCCTCTAAAAAATCCCAAGACCATCCCTTTGGCTCCGGGGGCCTA

CCCCACTTGTCCTACGATCACCAAGCCCATGACCTAAGTTTGGCATTCGC

AAGGCTTCACAAAAACTCGTGTGGTAGTGGGAGTGGACCCGCATTCAACG

ACTTCGATTTCTCCATTTTGGGAATGCCAAACGGCGACGTTTTGAATGGC

TTTGGGTATAATTCTCAAAGCATGTATTATGGAAATGACAACAACATGGG

AGGAATCGAAAGTAATGGAGATCAAATGAGATTACAACCGTACGATCAAG

ATCATCATCATCATCATAATCAACAGTATAGCAATGCTACAACGACGGCA

GTGACGGTGACAACGATGAAGCAAGAGCTATTTGGAGGACGAGACATCAA

TGGAGGTGACCAAAGTAAAATCCTTTGGGGATACCCAAATTGGCAAATGA

ACAATAATAATAATGCAATAGATTCAAACACCACCACCACCACGACGATG

ATGGCAGCCATGGATTTCGATTCGGGTTCGGCTCGCGAAAGCTGGAACAA

CAATGCCTTCACTAACGCTTCTTCTTGGCATGGCCTTCTCAATAGTCCAC

TCATGTAA

>ClDof8

ATGTTGCCGGAAAAGCTCGCGCCCGGTGGTTGCCGGCCGAAAAATTCTCA

GGCAAAACCCACATCGGATCATAATCAGCAGGCACTCAAATGCCCTCGTT

GTGATTCCCCCAACACAAAATTCTGTTACTACAACAATTATAGCCTAACT

CAGCCTAGGTATTTCTGCAAGACCTGCCGTAGATACTGGACCAAAGGTGG

GGCGTTGCGCAATGTTCCCGTAGGCGGCGGCTGCAGGAAGAACAAGAAGG

TCAAATCATCGTCATTGAGCCTTCCGTCATCCAAGGACGACTCCGGTTCA

TCCAATCCGGAAATCGGCCGGTTGGGTTTCTTCGTTAACGGGTTTTCGTC

GGGTCAATTGGGTGGAAATCGGAACTCGTCATTCTCTACTGCTCCGACGA

CGGGTCTCTACGATCAGTTTGGAGTTTTTTCTCTCGATCAATCGAGAAGC

TTAAATTCCTTCATTCCTTCCACCGGATTTGTTAATTCACTGAATGTGGA

CACGAGTCTGGCGTCTTCGATTGAATCTTTGAGTTCTATGAACCAAGACC

TTCACTGGAAACTGCAGCAGCAAAGACTGGCTATGCTATTTGGAACGGGG

CACAACAATACCGCCATTGATAAAAACAGGGGAGCACTTGAAGATCACGG

CCATGAACTGAAACCATGCTCGTTTCAGAATCTGGAGATCTCGAAACCGG

AGGCTTGCAATTCTTCCGATTTCAGTAACGCAAGAAACGAAACCGTGGCC

GTGACCGGAGCCGGAGTCAGAGCCGGAGGAGAACCTGCGGCCGCCGATGA

GTGGTTCTTTGGGGATTCTTATACAGCCCCCTCAATGACGATGGGTGCAG

CGGCAGCGGGATATAACTCCGGCGACAGCGCCGCCAGGTGCGATGGTGTT

CAAGAGTGGCACGATTTGCATCCATACACCCATTTACCGTAG

>ClDof14

ATGCTGGCCATTTCATGTCCAAAAGTACAGCAAGAAAACAGAAAGCCAAT

GAGGCCACAGCCAGAGCAAGCTCTGAGATGCCCAAGATGTGATTCAACCA

ATACCAAGTTCTGTTACTACAATAACTACAGTCTTTCCCAGCCAAGGTAC

TTTTGCAAGTCTTGTAGAAGGTATTGGACTAAAGGGGGTACTCTAAGAAA

TGTTCCTGTCGGTGGAGGCTGTAGGAAGAACAAAAGACCATCACCTTCTA

CTTCATCTTCTTCATCTCCTTCTTCAAAGAGATCGCATGATCAAGCTGCT

AATTCTAGCCGTCTAATGCTCACTGATACTCATCTTCCATCTTTAGCCTT

TGAGTCCACTGACCTAAGTTTGGCATTTGCTAAGCTCCAACATCAATCAA

ATGGGCAGATAGGTCTCTTTGATACAACCCACAACAACTTTCATAACTTG

TACTCTGGGTTTGGTACTGGGAGTTCTGGGGAGGTTGAAAATGGTGGATT

TCCTCATTTTGAAAATTATAACGGCAGTGGTGTTGCATCTACAGCCACTG

CTACAGCGATGAAGCAAGAGTTGGTTTGCAATGGAGATCAAAATAATATG

GTTTTGTTGGGATTTCCATGGCAGTTCAACAATAATGGAGATGGAAATTA

CATGGGTGATCTTGATGCTGGAAGAGAGAGCTGGAATTTCAATGGAAATG

GAATAGCTTCATCTTGGCATGGACTTCTCAACAGCCCTCTAATGTAA

>ClDof6

ATGCCTTCCGACGCCGGCGACCACCGCAAGGCTGCCACCAAGCCCCTTGG

CTCCGGCGGTGGTGTTTGCCCTCCGCCGGAGCAAGAGCATCTTCCTTGCC

CACGTTGTGATTCCACTAATACTAAGTTCTGTTACTATAATAACTATAAT

TTCTCTCAGCCCAGACATTTTTGTAAGTCCTGTCGTCGTTATTGGACTCA

CGGTGGGACTCTTCGTGACATTCCCGTTGGCGGTGGCAGTCGGAAGAATG

CTAAACGTTCTCGTACTTGTATTCCGTCGTCTGCGGCGGTTGCTTCATCT

TCATCTGGGTCGTTGCCTAACTCTATTTCTCGCTTGGATCATCATTCGTT

ACCGGCGACTCCGGTGTTGGTGCCTCTCGTCAGTGGCCACGGTGGTGGCG

TCGGAGGGGACTTGAAGGTGGGCGGTGGGAATATGTGTGGGAGCTTTACG

TCGTTGTTGAACACGCACGCGCCTGGGTTTTGGGGTTTGGGTGGGTTCGG

GCTTGGGCTTGGGTCGGGGTTCGAGGATGTGGGCTATGGGGCGGCTAGTC

CTAGGGTGGCTTGGCCGTTTCTTGGACTGGGCGACGGAGGCTCTGGGGTC

GGCGGCCACGGTGGAACGCCGAACGCGTGGCAGTTTGAGAATGGGGACGC

CGCCGGATTTGTTGGGGCTGCGGAATGTTTGTCTTGGCCGGAATTGGCAA

TTTCCACTCCGGGGAATGGGCTCAAATGA

>ClDof13

ATGGCTCACTCTTCTCTTCCAATCTTCTTAGATCCTCCAAATTGGCATCA

ACCGAATCAGCCAGCAGCTGCAGCAACAAATGATGTTCATCAAGACCCAC

GTCAGCTTCCGGCAGCATTTCTACAACCGCCGCCGCCGCCAACGGCAGGT

CATGGAGGAGGCGGAATCCGGCCGGGTTCTATGGCGTATAGAGCCCGGCT

GGCGATGATTCCCCAGCCGGAGGCAGCACTAAAATGCCCTCGTTGTGATT

CTACAAACACCAAATTTTGCTATTTCAACAATTACAGCCTTTCTCAGCCG

CGCCACTTCTGTAAAGCTTGCCGCCGGTACTGGACCCGCGGCGGCGCTCT

TAGAAACGTCCCCGTCGGTGGTGGCTTTCGCAAGAACAAGAAGAAGAAGA

AATCTAACCCGTCTAAGTCCCCCACTGCCTCTCAATCTCAAATGGGTAAT

TCAAGATCAATAATGTCAAGAAGCTGCAACAACATGGAGAGCAGTAGTAC

AATGAGGGATTTCCCATCACATTCTTCTTTGCCTTCATTAAATATCCTTC

CATCTCTTCAACAACATTTTTCTCGTAATTTTGGAAACAACTTCCCCGGA

ATTCACATCAATAATTCCGCAGTCGCCAGAGAATACGACGTCGAATGGCA

ACAACAACAACAACCATTCATCGTGGCCGGACTAGAATCTCCGACGACTG

CGACAACAACTGCCGCAGCATACACACAAGCTGAAGTCAACAACAACGGC

AACAACAACAACTTTGTTGCTAACTCATTACAACAAGCAGCTGGTTTAGT

ACAATACAATAATAACTGTCATCAACAACTGCAAAATCTGACACCCTTTT

CCAGAATTCCTATGAAAAATGAAGAACAAAACCAAGAACAAGGAATATTA

TTATCAAATTTCTTACGACCAAATGATCACAACAGCCAGTTTTGGGGAAG

TAATCATCAAAATTCATGGACGGATCATTTGTCAGCTCTCAGTTCTTCTT

CAAGCAGCCATCTTTTGTAA

>ClDof25

ATGGAAGACATAAACCCTAATTCAAGCACAAGATCATCACCATCATCATC

ATCCATATTGGAGATCAAGAAATCAACAAAGCCTCCTAAAGATCAATTGA

ACTGCCCAAGATGCAAGTCCAACAACACAAAATTTTGTTATTACAACAAT

TACAGCCTTACACAACCAAGATACTTTTGCAAGTCTTGTAGAAGATATTG

GACTGAAGGTGGGTCCCTCAGAAATGTCCCTATTGGAGGAGGCTCAAGAA

AGAACAGATCAAAATCAACAACAACAATAACAATAATGTCTAATTCTTCA

GCTCCTTCTTCTTCATCTTCATCTTCATCTGATCATTTCAACAACAACAA

CCCAAAATTACCAAATTACCCTGCATCTCAATTTTCTTCACAGAACCCAG

TTGGAAAGGATCTTAACCTTGCTTTCCCTTGTGCCATGTCTCATTATGCT

GATCATCAGCTCTCTAAGGTTGAAAATGTCAACAATAGTAATTATAATTG

TGGAATTGGTTTTAGGGGTTTGAGTTCTTTTATCCCAAATCTAATGCCAA

ATTCCAATAACAATAATACTAATTTGGGGATTAATAGTAATGGCCAATAC

CCAACTGGGTTTTCTTTGCCTGATCTTGATGAGTTTAAGCCAAGTTTGGG

TATTAATTCTGTTGATGGGTTTGGAGATGGAAGAAATTTGCTTCCTTTTC

AAGAATTGAACCAACAATCTGCTGCCCCAAATGAAGGTGATCATCAAAAT

GAGAGGCAACAAGGGAATAACATTTCAACTGGATATTGGAGTGGGATGCT

TGAGGATGAATCATTTTAA

>ClDof23

ATGGATTCTGCTCACTGGCCTCAGGTTGAAGTTAAAAGCATGGAATTAGA

AGAAGAGGGAATAAAGGCAGTTGTGGAAAGAAAAGCAAAGGCTAGGAAAG

ATCAAATATTGAACTGCCCAAGATGCAATTCCAACAACACAAAGTTCTGT

TACTACAACAATTACAGCCTTTCACAGCCAAGATACTTCTGTAAGTCTTG

TAGAAGATATTGGACAGCAGGTGGGTCTTTGAGGAACATTCCAGTAGGTG

GGGCCTCCAGGAAGAACAAGAGACCTTCAGCTAATTTTTCATCACCTCCT

TCAAAGAACAATCAAAAGAACTGTTATAACGATAACAACAATAATGGTGA

TGATGATGATCATCAAGGCATTTCCCAATTGAATATCAATACTACTTGTT

CTTCAACTATTACTAATACTGCTACTTCTTGTTGTTGGCTGTCATCTGAT

GATCATATGAATAATCAAATAATGCTGAGAAGTAGTGGAATAATGTCTCA

AAGGGAGCTGATTCCTTTTATTCCAATGCCTGCACCAGCTCCTCCACAGC

CCAGTACTGCTGCTGCTCTGGAGGATTTCAAGCAACTTTCAACTATCATT

TCTACTGATCAAAATGGAGCCAAATTGGGGGATGTTCCTGCCTTTTGGAA

TGGCATTTTTGGAGGAGGTTCATGGTGA

>ClDof24

ATGGCTGAAGTTGAAATTGGAGATCCAATCATAAAGTTGTTTGGGAAGAC

CATTGCTTTGCCACTGAACCATCTGGATTTACCTTCTGATTCCAAATTTC

TTTCTTCAGAAACCTCTGTACTTAAAGCTGGCGAAAAGGAAACTTCATGT

GAAGCATTGGCAACAGAAAAACGAGGGATTAGCAGTGGAAACCAAATCAC

CGATCAAACAACATCAGGCATGAGTGAGAACCCATCAGAAGAAAGAGAAA

TTTCATCTCCAAAAGCTTCCAAAAATGAAGAACAGAGTGAAACAAGTATC

TCCCCGGACAACAAAACCTCAAAAAAACCAGACAAAATTCTTCCTTGCCC

TCGATGTAACAGCATGGACACTAAATTCTGTTACTACAACAACTACAATG

TCAATCAACCTCGTCATTTTTGCAAGAATTGCCAAAGATATTGGACTGCT

GGTGGGACCATGAGGAACGTTCCAGTCGGTGCCGGCCGGCGGAAGAACAA

AAGCTCCTCATCTTCACATTTTCGTCAGCTAATAATTCCCGACGGTGGAA

TTCATCGCCATCAATTTCTAGGGAACAATGGAACCTTTCTCACTTTCACC

TCAGATTCTTCCATTTCCGACTCACAGAACTGTAACCCAAGTGGGTTTCT

CATTAGTACAGAAAATGGAGATGATCATTCCAGTAAATCTTCCATTACAG

CTTCAAATTCCTCGGAGAAAGATGGTAAAATGATCTCACAGCAATCAGTA

GTGAAAAACGTTTTGCCTTTTCAACCTCAATTACAGACATTTACAGGACT

TTCATGGCCTTATTCTTCAACGGCTCCACTGCCGTACTACCCGCCGGCGT

TTCCAGTGTCGTATTACCCTGCACTGCCGTACTGGGGCTGCACAGCGCCG

CCGTCTTGGACAGTAATTAATCACAACGGTCAAAATTCGTTTACTAATTC

CTCTGTTTTGGGGAAACGTTTAAGAGATGGGAAATTGGTTAGAAGTCCAG

CAAATTCAGAGTGTGAAGAGATTGTTAAACAGAGGAATTGTGAAGAACCC

TGTTTCTGGAATCCAAAGACAATGAAAGTGGATGATCCAAATGAAGCTGC

AAAGAGTTGTATATGGTCAACACTTGGAATTAAGAATGAAAAAGCTGGTG

CAAGTACAATCAATGGAGGTAGCCTTTTTGTCTCTTTACAACAATCTGCA

AAGGGAAAGGAAGAAAACCACATTGAAAGTTGTTCGCTTTTACAGGCAAA

TCCAGCTGCTTTTTCTCGGGCTTTGAAATTCCGTGAGATTGCTTAG

>ClDof36

ATGAAGAAAGAGAACCCAGAAAAACACCATGAATTTTTCCTCCATTCTCC

TGCTTATCTTGATCCTTCCAACTGGCAACAGCAAGTCACCCATCAAGTGG

GAAGCAGTAGTAGCACTGGAGTTAGTTCTCAGCTTCTACCACCGCCGCTG

CCGCCGCCTCCACCGCCTCCTCCCCCACCACCCCATGGCGTCGGCGGTGC

CAGTTCGATCCGCCCTGGTTCGATGGCCGAGCGAGCTCGAATGGCTAATA

TTCCGATGCCCGAGGCAGCATTGAAATGCCCAAGATGTGAATCAACCAAT

ACTAAGTTCTGTTACTTCAACAATTACAGCCTCACTCAGCCTCGCCACTT

TTGCAAGACTTGTAGAAGGTACTGGACCAGGGGTGGGGCCCTACGGAACG

TCCCGGTTGGGGGTGGGTGCCGGAGGAACAAAAGAAGCAAAGGGAGCAGT

TCGAAGTCGCCACCGGTCAGCTCGGACCGACAACAAACCAGCGGGTCAGG

CAATTCTTCATCAAGTGCAATAGCTTCCAACAACAGTGGAGGCCTTTCCC

CACAGATCCCACCACTCGGCCGGTTCATGGCTCCTCTCCATCAACAGCTC

AGTGACTTCGACATAGGTGGTTTCAGCTACGGGGGTGGCCTCTCAGCTGC

TGCCACCGCCACCGGTGACCTGAGTTTCCAATTGGGCAACACTACTTTAG

CTGGAGGAACAAGCATTGGATCTCTCTTGGGATTTGACCAACAATGGAGG

CTCCAGCAACAACCTCCCCAATTTCCCTTCTTGTCCGGGTTAGACCCTTT

CGACGGCGGTAGCAGTAGCGGCGGAGAAGGACCCGGACCCGGACCCGGCT

GGCAAATGAGGCCAAAATTACCATCAAGTTCAAGGAATGTAAGCCAAATG

GGGAATTCAGTGAAAATGGAAGAAACCCCAGATCAAGTTAATAATGCTGG

AAGACAATTTCTTGGGAATGAACAATATTGGAGCAGTGGATCAATGGCAT

GGTCAGATCTTTCTGGTTTCAGTTCTTCATCTTCAACAAGAAACCCATTA

TAG

>ClDof19

ATGCAAGGGGCTCCCAATGAAGATCAAACCAAAACTTCAACAATAATCCA

AAAACAAAAGTGCCCTCGTTGTGAGTCTTCCAACACCAAGTTTTGTTACT

ACAATAACTACAGCCTTTCACAGCCTCGTTACTTTTGTAAGTCTTGTCGA

AGGTATTGGACTCATGGTGGCACTCTCCGTAATGTCCCGATCGGCGGCGG

CTCGAGAAAATCCAAGCGCCCTAAGACCACAATGCCTTCCTCTTCAGCTT

CTTCCGACACAACTACCCTAACTCCGCCACCGCCTGCTGCCGGAGGATTA

CCTCTTGATCATCTTGCAACTTCTTATTCTAGTGCTGCCTTTTTGCCATC

TTTGGATGTGGGGGCTTATTCATTTGGAGGATTACCTTGGACTACTTCAA

TCACCAACACAATTATTCATTCTTCTTCTTCAGGTAATAACCAAAATTCA

AGCCTTGATATTGAAAATAATTCTTGTACTTTGCTCCCAAATCATCTCCC

TCCCTATGCCCATCCTCCCTGA

>ClDof9

ATGGGTTTGAGTTCAAAACAGGTTTCAATCGATGGATTTGATTGGAGCAA

GGCGCTAATGCAAGCACAGAAACTGGAACTTCCAAAATTGGCTCCGAGCT

CCGGCGTGAAACGATCACAACACCAAAATCAGAATCAAATTCAGCCACAG

ATAGAGCAATTGAAATGCCCCAGATGCGATTCAACCAACACAAAATTCTG

TTACTACAACAATTACAACAAATCTCAGCCTCGCCATTTTTGCAGAGCTT

GTAAACGCCATTGGACCAAAGGTGGAACTCTACGAAACGTCCCTGTTGGT

GGCGGCCGTAAAAACAAGCGCCTCAAAAAAAAACCAACCCCCAAATCCAC

AAAATCTTCCTCCTCCGCCGCCGCCGACGTGATTAATCCCCAAATGGATG

TTCATCATTTTCAAAACCTTCCTCTGTATCAGGGGCTAATTTTTTCCCCG

CCGTCGTCCAGCAATTGGGCGGAATGCGAAAATTTCACCACAAATTACGG

GATTCTAAATTCTCAACCCCCCGATTTCTCGGCCGTTTCAACAACCACCT

CAACACATTCTCCAATGTCCCCAAAATTTAATAACTATTCAGATCAAGAA

TTGAAACCCACAGAAACAGAGCAACCGGCCAACAGCACCAGTACTCATCA

TCCATGGCAGCTTCCATCCACCGCCTGCGGCGTCGCCGACATGTCCAACA

GCTACTGGAGCTGGGACGACATCAACACCTTCGCCGCAACTGATCTCAAC

ATCCCATGGGACGACGACCATGACATCAAACCTTGA

>ClDof27

ATGCTGGAAATTAAAGACCCTGCCATTAAAATCTTCGGCAAGGAGATTCA

ACTTCCGGCCGATTGTGAGGTTTCGTTGATCGAAAGCGATGATTCCGTTT

CTACTTCCGATAAAGAAAGCGGAGATGGAGCACTACAAAAGGATGTAGGA

AAAGCCACTGAGAGTTTAGCTGCTAAAGATGGAACTCTTCATGATTCAGA

GGATTCTGCATGTGTACAAACAGCCAATGAGGCGCACATGAATCCAGAAG

TGGTATCGATGGATGAAAATGACAAATTCGCAACAAGCAAACCCGAGAAA

GAACAGAATGATGCACCCAACTCAAAAGAGAAATTGAAGAAGCCGGACAA

AATACTTCCATGCCCCCGCTGCAATAGTATGGAAACCAAGTTTTGTTATT

ACAATAATTATAATGTCAACCAACCACGCCATTTCTGTAAAGCCTGTCAA

AGATATTGGACTGAAGGCGGTACCATGAGGAATGTCCCCGTTGGAGCTGG

CCGCCGAAAGAACAAGAACTCTGCCTCACACTACCGACAGATTACAATCT

CTGAGGCTCTTCAAGCTGCACAAATTGATATTCCTAATGGAGTCAACTGC

CTAGCAACCAAAAGCAATGGGCGAGTCCTAAATTTCAGTGTAAACGCACC

TGTATGTGAATCTATGTCCACTGTCCTCAATCCGGCAGGAAGAAAGGTCT

TGAATGGGACTAGGAATGAATTTCATAGACTCGATGACCAAGGAATTAAA

GCACCTTGCAAGGGTGGGGAAACTGGGGATGATTGTTCCAGTGCATCTTC

AGTGACGATGTCGAGTTCAATGGAGGAAGGAGCCAGGAGATGCCCTCAAG

AACCACAGATGCAGAATATCAATGGTTTCCCTCCTCAAATTCCATATCTC

CCTGGTGTTCCTTGGCCTTGTTCATGGAATCCACCAATGCCTCCACCGGC

CTTCTGCCCTCCTGGAGTTCCTTTATCATTCTATCCTGCGACATACTGGA

GCTGTGGCGTTCCAGGTGCTTGGAATATTCCTTGGTTCCCTCCACAACCC

TGTTCACCAAATTCTGGTGCAAATTCACCTACACTGGGGAAGCATTCCAG

AGATGGTGACAAACTCCAGGCTGATAATTCTGAGAATGAAGAACCTCCAA

AACAGAAAAACGGATCTGTTTTGATTCCCAAAACTTTGAGGATTGATGAT

CCGGATGAAGCCGCTAAAAGTTCAATATGGGAGACACTTGGTATTAAGAA

TGATTCAATCAAAGCTGTTGACCTGTCTAAGGTCTTCCAATCAAAGGGTG

ATCAAAAGAATAGGGTTTCTGAAGTGTTATCACCAGTTTTGCAAGCTAAC

CCTGCAGCCTTGTCAAGATCTCTTACTTTCCATGAACGTTCGTGA

>ClDof11

ATGGCTTCAACTTCTAGGATTATGGACAAACCCCGTCAAGAACAACAACA

ATTACAACAACCACCATCCGCTACTCTAAAGTGCCCTCGTTGTGATTCTT

CCAACACCAAATTTTGTTACTACAACAATTACAGCCTCTCTCAGCCAAGG

CACTTTTGCAAAGCTTGCAAACGCTATTGGACTCGCGGCGGAACCCTAAG

AAATGTCCCCGTTGGTGGCGGCTGCCGGAAGAACAAGCGCCTCAAAACTT

CTTCCTCCTCAGTCAGCGCTACTTCCACCTCAACCCCAAGCCAAAACCCT

CGTCTTCACAATTCCATTAATTCTAGTACTACTCCTAATATTATTTCTTC

AAATCATATCAACAGCCCAATGTTCTTTGGGTTGGATCCCATTGGTAGTG

GTGGTTTAGGGTTTTCCTCTTCTGGCCTTCTCTCCCACTTCCATGATCTC

CATCAACAACCCTTTAATTCAAGTCAATTTCATCATCCTGCCATCTCCTT

CGATCGAAATTCTCACGTTTTGGGTAATTTCGAACCAAGTTTGATGACAC

CAATGAAGGAAATCAAGATTGAGGGTTTGAATAGGTTGTACCAGAATCAA

TCAGAGCAAATCGATCTATCGAATTTCTCTGATCCTTCTTCGGTCTATTG

GAACTCGGGAACAACGGCAACAGACAATTGGCACGATCCGACAAATAATA

ATGGGTCCTCAATTGCTTCTCTATTCTAG
