# Supplementary material for: Genome-wide characterization and expression analysis of the Dof gene family related to abiotic stress in watermelon
Source: PeerJ. 2020 Feb 17;8:e8358. doi: 10.7717/peerj.8358 (PMC7032062; doi:10.7717/peerj.8358)
Supplement: Supplemental Information 5 [file peerj-08-8358-s005.doc]

**Table S4.** The gDNA sequences of ClDof members.

>ClDof29

ATGCAAGACCCATCAACGTTTCAGCCGATTAAACCCCAATTTCCCGAACA

AGAGCAACTTAAATGTCCTCGTTGTGATTCTACGAACACCAAATTTTGCT

ATTACAATAACTATAATCTTTCTCAGCCTCGTCACTTTTGTAAGAATTGC

CGTCGGTACTGGACTAAAGGCGGCGCTCTTCGCAACATCCCCGTCGGCGG

TGGCACTCGGAAAAACTCCAAGCGTGCGGTGGCCGCCACCGTCAAACGCC

AGCCGTCTACCTCGTCTTCGTCTTCTTCAACCTTGACGAATCCAAACCCG

ATCACGGCGCCGGATCATAACCCGATCGGGATTTACGGCGGTGGGCTGGA

TATTCCAGGGAGCTTCAGTTCGCTGCTGGCGTCAAATGGGCAATTTGGGA

ATCTTTTGGAGGGTGTTGATCCGAATCAATCGGAGCTGAAAATGGTGGAA

TTGGGTGAATTCTCTGGGTCTGGCAGGAAATCTGCAGCGGAGGAACAGAG

CACCGCCGTGCCGGAGAATTATTTGGGTGTTCTGCAAAATGGGGATTCTA

ATTGTTGGACCGGTGGCGGGAGTCATGGGTGGCCCGATCTTGCTATTTTC

ACACCAGGGTCAAGTTATCAGTAA

>ClDof3

ATGGGAATTGATTATTGTGGATTTGATGATCAGTTTGCAGGTGGTGGAGT

GGTTGTTAAACAAATGGAAGGAGGTGGATCATCAAGTTCTTCAAAGCCTA

ATAACAATAACAACAACAACAATAATAATGGTAATTTGTTGGAGAGAAAA

GCAAGACCTCAAAAGGAGCAAGCTTTGAATTGTCCAAGGTGCAATTCGAC

CAACACAAAATTCTGTTATTACAACAATTACAGCCTGTCTCAACCAAGGT

ATTTTTGCAAAGCTTGTCGGAGGTATTGGACTGAAGGTGGTTCTTTAAGG

AATGTTCCAGTGGGTGGTGGTTCAAGGAAGAACAAAAGATCTTCACTTTC

ATCATCAGCTGATAAGAAGATCAACGCCTCTGATCAAAACCCTAAAATCA

TCCATCATGCTCATCAAGATCTCAATCTTGCAATCTTCCCTCCAAACAAT

AATAATAATAATAATACTCCTCCTATTCCTTCTTCTACTTCTACTTCTTC

TTCTTCTCACCTTTTGGGTTCTTTCATGACGGCGGCACCGGCGGCCGGAG

TGTTCAATGGCGGTGGTGGGTTTGGATTGAGCGAGCTGAAGCCATCAAGT

TTAAGTTTTTCTTTGGAAGGGTTTGAGAATGGTGGAGGGTATGGGGATCT

TCATCATAATCATCATCATCATCATCATCATCATCATCATCATCAAGATC

AGACGGCTGTGATGTTTCCAATGGAAGAATTGAAGCAAAGTAATGGAAGA

GGTGAATTTGAGGAGAATAGGGGAGGAGGAGGGCATGGAGCTGGGGATAA

CAATTCAGGTGGTGGATTTTGGAATGGAATGTTGGGAGGTGGATCATGGT

AA

>ClDof35

ATGGTGGAAATATCACCAAATTGTCCAAGGTGTGGCTCTTCCAACACTAA

GTTTTGTTATTACAACAACTATAGCCTTACCCAACCTCGCTACTTTTGCA

AAGGCTGCCGCCGCTATTGGACCAAAGGTGGTTCCCTTCGTAACGTCCCC

GTCGGTGGTGGCTGTCGGAAGAACCGACGACCTCCCAAGCCCTTAAACTT

AACCCCATCTCCTTCAAACCCTAAATTTCACCATAGCAATGATGGCAATT

GCCATCGCAACCACCGTGGTCTCTCCGACCACGACATGTCGCCGACCATT

GATTTGGCGGCCGTCTATGCCAATTTTGTGAACCAGAAGGCAGACACCCC

CGAGACTCTCCCGACAGCTAATACCACGTCGAACGCAACGGGTCACTTTT

CGGTTTCCGGTGAGAGTGGTGGGGTTGGATGTGGCAATTATTGTGAGTTT

TCCATGCAGGAATGTGGATTGGGGGCCAATGATTATCAAGTGGCCGGTTG

CTTTGGATTAGAGGAGAATAATAATGATCACCAAGTGGATAACATTGAAG

TGGCGGCGCGTGAGCTTCAATTGCCGCCGTTGCCGGGGGAGGACATGCTA

GGAGGTTGGGGAAACCCTATGATAATGGTGAATAATCATCATCGTTTGCA

ACCAACACGAGTGGAAATGTTTGGAATGGAAACTCAAGACCCAAATTTGC

TGAGTGGTAATTGGAGCCCTATTGATTTGTCAAATTATGATACATTCTCA

AGGGCTTAA

>ClDof11

ATGGCTTCAACTTCTAGGATTATGGACAAACCCCGTCAAGAACAACAACA

ATTACAACAACCACCATCCGCTACTCTAAAGTGCCCTCGTTGTGATTCTT

CCAACACCAAATTTTGTTACTACAACAATTACAGCCTCTCTCAGCCAAGG

CACTTTTGCAAAGCTTGCAAACGCTATTGGACTCGCGGCGGAACCCTAAG

AAATGTCCCCGTTGGTGGCGGCTGCCGGAAGAACAAGCGCCTCAAAACTT

CTTCCTCCTCAGTCAGCGCTACTTCCACCTCAACCCCAAGCCAAAACCCT

CGTCTTCACAATTCCATTAATTCTAGTACTACTCCTAATATTATTTCTTC

AAATCATATCAACAGCCCAATGTTCTTTGGGGTACATAACACTAACCCAT

CTTCACTTTTTAATTCTTGATCTTCTTTCTGTGTCTAATTAATGGTTTTC

TTCTTAACCTTTCCTCTCCTTGCAGTTGGATCCCATTGGTAGTGGTGGTT

TAGGGTTTTCCTCTTCTGGCCTTCTCTCCCACTTCCATGATCTCCATCAA

CAACCCTTTAATTCAAGTCAATTTCATCATCCTGCCATCTCCTTCGATCG

AAATTCTCACGTTTTGGGTAATTTCGAACCAAGTTTGATGACACCAATGA

AGGAAATCAAGATTGAGGGTTTGAATAGGTAACTATCATGACTCAAAAGC

TTAAGCTAATAGACGTGTAATTCATATAATTGTTTGTTCGTATTCATTTA

TTAGGTTGTACCAGAATCAATCAGAGCAAATCGATCTATCGAATTTCTCT

GATCCTTCTTCGGTCTATTGGAACTCGGGAACAACGGCAACAGACAATTG

GCACGATCCGACAAATAATAATGGGTCCTCAATTGCTTCTCTATTCTAG

>ClDof21

ATGTTGGATTCTAAAGATCCTACTATTAAGCTTTTTGGCCGTAATATTCC

ACTTTCCGAAGACGGGGAACCTCCGGCGATTCTTTCCCGCGACTTCCCTT

CTCAGAAACATGCAGAACCAATAAAGGTACAGAGTGTTCTAAACAGAGAA

GCTCTCTTATACTTTAATCCAACTGCTGCCCATTTCACCAAATGCTTATA

AAAATTCTGGGTCGTTTTTACTTTTTCACTTCAGTTTTTTCCTACCTGAA

AAAAAAGCCATTTTTTCTTTTCTTTGTTTTTACTTTTGGTTGATTATGTA

TCCTTCCAGTGTTTTGTCCTTTTCATCTGTAATTTGGATTTTTTTTTTAT

TTTTTTATTTGTTGTGTTTTTCTTTGGTTATACATACTGTTAGGAATCCG

TAAATGTTCTTGGGTTATGGTGAATTCTTCTTTCTCTGTTCCTCTGTTTT

TCAATCAGTTTTCCAATCATTGACAGAGAAAAAAGTTACTGATTTACTCG

AAAAGGAAAAAGGGGGTTTCTAACTTTATATCTTTCTCAATTGGGATTTT

AGCAATGAACTAAACAGAAACTGGATTAACTTAATTTCTATATGGATCAT

GACACTTTCTTTTTGTGGATTTCAGGATGATGCTGTTGATGATCCTGAGA

AACCTGTTGATGATTCAGATGATTCAAGAAATCTAGAGAGAGAGGAAGAG

GCTAGCGTGAATCCCAAAACGCCATCTATAGATGAAGAAACTGCGACGCC

TACTAATGGAGAACAAGAGAGTGAGAAACCTAATTCAGAGAAAACCCTAA

AGAAGCCTGATAAATTGCTTCCATGTCCTCGTTGCAAAAGTATGGAGACA

AAGTTCTGTTACTACAACAATTACAATGTAAATCAACCTCGCCATTTTTG

TAAAGCTTGTCAAAGATACTGGACCGCCGGTGGGACTATGAGGAACGTAC

CGGTAGGAGCTGGTCGTCGGAAGAGCAAAAACTCTGCTTCTTATTACCGT

CACATCACGATCTCTGAGGCTCTTGAAGCTGCTCGAATTGAGGCTCCAAA

TGGAACCCACAAACCAAAATTCATTGGCAACAATGGCAGAGTTCTCAGTT

TCAATTTGGATGCACCGAGTTCTGATGCTGTGGTGGGCTCTGTTTTAAAC

CTTGCAGAAAACAGAGTTTTGAGTAATGGGGTAAAGAAGTTTGAAGAGAA

AGGATCTGAAGGATGTGATAAAAGCTCGAGTTTGTCTTCTATGGCAGTTC

AAAGTTCATCAGAATTGAAAATCAATGGCTTCCCTTCTCAGATTTCATGT

CTTTCTGGAGTTCCATGGCCTTTTATTTGGAATTCATCAGTTCCACCACC

AGCTTTTGGCCCTCCAGGATTTCCCTTGTCTTTTTTTCCAGCAGCTCCTT

GGAACTGTGGGGTTCCAGGACCATGGAACACTCCATGGTTTTCACCACAA

CCTGAAAAATCTGTGCGTTCTGACTCTGAAGCTTCTTCAACACTGGGAAA

GCATCAAAGAGACAATGAAACGGCCAAGGAAGATGCCATTTCAAGTAAAG

AAGAGGGTGTTAAGCAGAGAAATGGACATGTCTTAACCCCAAAGACTTTA

AGGATTGATGACCCAAGTGACGCTGCAAAAAGTTCCATATGGGCAACACT

TGGGATAAAGAATGAATCAATCACTGGAGGAAAAAATCTGTTCAAAACCT

TTCATCCCAAAGGCCATGAGAAGGTTCATGTTGCTGAAGCCTCATCAGTT

TTGCAGGCAAATCCTGCAGCCTTGTCGAGATCTCTCGTCTTTCACGAGAG

CTCTTGA

>ClDof20

ATGGTATTTCCTTCCATTCCATCTAATTATCTTGATCCAACAACAACCCA

TCAATGGCATCAGGTAAACCAATTAACTTCTTAATTTATTCACTACAATT

TTTCTTTTCTAAAAAAATGTTTTTATCTCAATATAACTAAATATTTTTTC

AACAGCAAACAGCAAGCCAACAATCTGGGAGTAGCAGTTTTCAGCTGGCG

GGGTCACCAGCACCGCCACAAGTTCGGCCGGTGTCTATGGCAGATCGGGC

TCGAATGGCGAACCTGCCGGTGCCGGAGACGGCACTGAAGTGTCCAAGAT

GTGAATCAACAAACACAAAGTTTTGTTACTTCAACAACTATAGCCTCTCT

CAGCCCCGCCATTTCTGCAAGACCTGCCGCCGCTACTGGACCAGAGGTGG

CGCTCTCAGAAGCGTCCCGGTCGGCGGCGGCTACCGCCGGAATACTAAAA

GAACCAAAAGTACCTCTAAATCTCCGGTTAACTCCCAATGTCAACCTACT

ACTACTGGTACTATTTTTTTTGGTCTAACAAATCATATATGAACTGGTGA

TGTGTTAAATTATTGCTTAGATCTAAAAAGTTTAACCCTTTTAAATCACC

ATTTTGATTGTAATTTTTACTTTTTAATTAAAATAATACCATGGTCTCTA

TTTAAGTTTCTGTTTTTCACTTTCTAGATCAACTTTTAGAATCTGAAAAT

TGGGTGATAAAAAAAGTTAATTTTGTTTTTTTTTTAAACTCATTAATTAT

GAACATAGTTAAACGTATGCATCTCTTCGACTACGACGAGATTATATGTT

CGAATTCTCCATCTCGAATATTGTTGCGGTAAAAAAACTACAAACTCTTC

TTATTAAATCTTCTTAAAATGTGTGGGGGAGAAAATTCGAACTTTTGATA

TTTTGATCGAGATTATATTATTTAGCTCAAGATTTTACAATAAATGAGAA

GTTAAAAGACATGTTTATTTAAAAATATTATAATATTTGTCATGTAATAC

TTTTAATTTGATAATTTATTGACTATGGGAAAAGTATATATTTTGTAGGG

TTTTTCTAACCTATGAAACTTTAAAATTAACCACATATAGTTTCAATTTC

ATTTTACATAAACTTTCAGAGGTTTCAGGAGGAGAAGCAATGAGATTAAA

TTACAATAATGCCATTTCCACACAAAATGAAGGAGCTGTGGATTCAACTC

ATCATCCCATTGGGATAGCCAACTTTTTAGGGTTTGATCAAATTCAATGG

CGACCACAGCTTCAACTTCACCAAACACAACAGTCTCCATTGCTTGTAAA

TTCAGCTCGGTTGCTTGGAAATGGTGGCATCGAGGCATCGCCGAGCTATA

GCATCGTTAGACAGATTCAAAAGAAGATGGAAGCGACGACGAAAATGGAG

GAAGATGGGCAACAAGTAGAGATGAATGTTGCAAAGCCATTTTGGGGAGT

TGTTGGAGGAAGTGATCATCGCCATTATTGGAGTGGTTTTAATCCTTCAT

CTTCTTCCCATTCATAA

>ClDof26

ATGCAAGACATACACTCGATCGCCGGAGGAAGTCGCTTGTTCGGCGGCGG

TGGCGGCGGAGACCGTCGTCTACGGCCACACCTCCACCACCATCAAAACC

ACCAAGCCTTGAAGTGTCCGAGGTGTGATTCTCTCAATACGAAGTTTTGT

TATTATAATAATTATAATCTCTCTCAGCCACGTCACTTCTGCAAGAGCTG

CCGTCGTTACTGGACGAAAGGCGGTGTTCTCCGTAACGTCCCCGTCGGCG

GTGGCTGCCGGAAAACCAAGCGCTCTTCCTCCTCTAAGTCTAAGGTCAAC

TCAGACGCAGCGGCGACTCCTCCTCCACCTCCTTCCCTCCGTGAACGCAA

ATCCACTTCTCATTCCAGTAGCGAGAGTTCCAGTTTAACCGCCACAACTA

CTACCGCTGCTGCCGCAGCGGCGGCAGCGACTGAGGCCGTGTCGGCTCCG

TCGTCTAATTCGGCCTCGACTTTATTGAACGTACAGGATACGAAATTGTT

CCCTGGTTCGACTACAAATACAAACCCTAATTTCGAAGGTACGGCGGCGG

CGGCGATTTCGGACTGCGGAATTTTCTCGGAAATCGGTAGCTTCACGAGC

TTGATCACATCGTCTAACGAGACATTGGCGTTTGGATTCGGTAACATGAC

GGACGTGACGGCGTTTACGATGAATAATCATCAGGCGTTGGCGAATCAAT

GGCCGCTGCCGCAGAGGATGATGAACGTTAACGATGAACTGAAGATGCAG

GAGATCACAGACGGCGGAGGAGGAGGGTACATGGATCAGACGGCTCAGGT

GTATCCATCAGGGCTTCAGAATAACAGATCAAACAACATTGGATTTGGTC

CGTTGGATTGGCAATCAAACGGAGATCATCAAGTTCTGTTTGATCTCCCC

AACGCCGTAGATCAAGCGTACTGGAGTCAAAATCAATGGAGCGATCAAGA

CCAGCCTAATCTGTACCTTCCGTAA

>ClDof8

ATGTTGCCGGAAAAGCTCGCGCCCGGTGGTTGCCGGCCGAAAAATTCTCA

GGCAAAACCCACATCGGATCATAATCAGCAGGCACTCAAATGCCCTCGTT

GTGATTCCCCCAACACAAAATTCTGTTACTACAACAATTATAGCCTAACT

CAGCCTAGGTATTTCTGCAAGACCTGCCGTAGATACTGGACCAAAGGTGG

GGCGTTGCGCAATGTTCCCGTAGGCGGCGGCTGCAGGAAGAACAAGAAGG

TCAAATCATCGTCATTGAGCCTTCCGTCATCCAAGGACGACTCCGGTTCA

TCCAATCCGGAAATCGGCCGGTTGGGTTTCTTCGTTAACGGGTTTTCGTC

GGGTCAATTGGGTGGAAATCGGAACTCGTCATTCTCTACTGCTCCGACGA

CGGGTCTCTACGATCAGTTTGGAGTTTTTTCTCTCGATCAATCGAGAAGC

TTAAATTCCTTCATTCCTTCCACCGGATTTGTTAATTCACTGAATGTGGA

CACGAGTCTGGCGTCTTCGATTGAATCTTTGAGTTCTATGAACCAAGACC

TTCACTGGAAACTGCAGCAGCAAAGACTGGCTATGCTATTTGGAACGGGG

CACAACAATACCGCCATTGATAAAAACAGGGGAGCACTTGAAGATCACGG

CCATGAACTGAAACCATGCTCGTTTCAGAATCTGGAGATCTCGAAACCGG

AGGCTTGCAATTCTTCCGATTTCAGTAACGCAAGAAACGAAACCGTGGCC

GTGACCGGAGCCGGAGTCAGAGCCGGAGGAGAACCTGCGGCCGCCGATGA

GTGGTTCTTTGGGGATTCTTATACAGCCCCCTCAATGACGATGGGTGCAG

CGGCAGCGGGATATAACTCCGGCGACAGCGCCGCCAGGTGCGATGGTGTT

CAAGAGTGGCACGATTTGCATCCATACACCCATTTACCGTAG

>ClDof12

ATGGCTGAGGTTCATAGTAGCAACACGGATGGAATTAAGCTTTTTGGCAC

TATGATTCATTTACAGACAAGAAAAATGAAGGAGGAACCGGAGAAAGGCG

GCGGTGAAGGTGATGAAACAGAGATGAAGAGGCCAGAAAAAATTATTCCA

TGCCCAAGATGTAAGAGTATGGACACCAAATTCTGTTACTTCAACAATTA

CAATGTTAATCAGCCTAGACATTTCTGCAAGGGTTGCCAAAGGTACTGGA

CGGCCGGTGGGGCGCTCCGGAACGTACCCATCGGAGCTGGCCGCCGGAGA

ACCAAGCCGCCGCCTAATTGCCGGACCCTTTCCGGCGAGCTGCCAGAGGA

TTATGGGCAGCTTTACGATGCGGCTTCTGGGATTATTCATCAGCTTGAAT

TAGATGCGGTGGAAGGGTGGCGTCTCACGTTGGCGGAACAGGATTTTAGC

TCGGTTTTTCCTTTCAAACGCCGGAAGATTATCGGTCAACACGGTCAAAC

GTCTTAA

>ClDof19

ATGCAAGGGGCTCCCAATGAAGATCAAACCAAAACTTCAACAATAATCCA

AAAACAAAAGTGCCCTCGTTGTGAGTCTTCCAACACCAAGTTTTGTTACT

ACAATAACTACAGCCTTTCACAGCCTCGTTACTTTTGTAAGTCTTGTCGA

AGGTATTGGACTCATGGTGGCACTCTCCGTAATGTCCCGATCGGCGGCGG

CTCGAGAAAATCCAAGCGCCCTAAGACCACAATGCCTTCCTCTTCAGCTT

CTTCCGACACAACTACCCTAACTCCGCCACCGCCTGCTGCCGGAGGATTA

CCTCTTGATCATCTTGCAACTTCTTATTCTAGTGCTGCCTTTTTGCCATC

TTTGGATGTGGGGGCTTATTCATTTGGAGGATTACCTTGGACTACTTCAA

TCACCAACACAATTATTCATTCTTCTTCTTCAGGTAATAACCAAAATTCA

AGCCTTGATATTGAAAATAATTCTTGTACTTTGCTCCCAAATCATCTCCC

TCCCTATGCCCATCCTCCCTGA

>ClDof18

ATGGACGACAACAACAACAACAAACAATTACCTACATCACAAGATCAAAA

GACTCAACAAAAACAACAACATCAAGAACAACTCAGATGTCCTCGTTGTG

ATTCATCAAACACCAAATTTTGTTACTACAACAACTACAGCTTGTCTCAG

CCTCGCCACTTTTGCAAAGCTTGTAAGCGTTACTGGACACGTGGTGGCAC

GTTGAGGAACGTTCCCGTCGGCGGTGGTTGCCGGAAAAACAAGCGTCTCA

AAAGACCTACAACTGCCTCCACCACCATCACGCATTCGTCATCGTCCCCG

GCTCCAACCGCCCCAGATTCGACATCTTCATCTTCAACTTCTTGTAACAC

TAACCCTCTTCATAATATCTTTTATGGCCCCACCTCTGATCTGAACCTTC

CATTTTCCGGGTATGATCATCATCATCATCATCATCTTCAAACTCACTTA

AATGCTATTGGTTTAGGGTTTTCTTCTCCTAATATTGATCATAGGGACTT

TAATCTTAATGGGTTCAATTCAAGTTCACTCCTTTCAGGTTATTCTAGTT

TATTTGGTAATAACAACAATAACCCATCTTCTAATTCAACCTCTATATCT

TCATTGCTTGCTTCTAAGTTTGATCCAAATCTTTTTGGAAACCTAGCTAA

TTCTCAGCCGTTTGATGATCATGTGATGGGATCTAACGGTGGAGATCAAC

TAGGGTTGAATGTGAAGGATGTGAAATTGGAAGATGGGATGAAGAGGTTA

AATTGGGAGGATGATGATCAACAACAAAATCATCAGACAGATGAAATTGT

TGCTCAATCTAATGATCATAATTCTCTTTTTGGTAATAATTGGGCCTCTC

AAAATTGGCATCATGATCCACCAAATCTTGCTTCTTCTATCACTTCTTCC

CTCATCTAG

>ClDof2

ATGAAGGCCTCATTGGAAGAAGAAATAAGGCAAAAATCATCATCAAGTGG

AAGAACAAAACAAAATAATAATGGAGATCATTGTGAGCAAGAATTAGGAG

GTCTGAAATGCCCTCGATGTGATTCCACCAACACCAAATTCTGTTATTAC

AATAACTACAGTCTCACACAGCCTCGTCATTTTTGCAAGACTTGCCGGAG

ATATTGGACTAAAGGTGGAGCTTTGAGAAATGTTCCAATCGGTGGTGGTT

GCCGGAAAACCAAAAAGCTCAAATCTTCTTCTTCTTCTTCCTCCTCCGTC

ACCACCGCGGCTCCACTGCCTGATCACTCCTCTCTTGCCTTCAAATTCTT

CGCCGGAATCTCACCGCCTCCGCCGCCACCGTCTGTTGATTTTGGTGCCA

TTGGAGGAGAAGGTTCTTGTTTGCATCTTGCATCTTCAATTGAATCTTTG

AGCTCCTTAAATCAAGACCTTCATTGGAAATTACAACAACAACGTTTGGC

TATGATTTTTGGAGACCAAGAACAACAACAACAATCACAAAACAAGGATA

ATCAAAATCACAACTTTATTCAACCCATTTTGTTTCACAATCTACATCAT

CAAAAGTCAGCTGCACCATCTACCACAACAACTACTATTAGTGATCAATG

GTTTTTTCCAAATCAAGTGACTGCCACAAACAACATAGCTTCAAGATCAG

TTGATGATGGGAGTGGGTGGAATAGTAATAACAATGATAATCATGGAGGA

ATTCAAGCTTGGGAAGATTTGCATCATTTCACTGCCTTCCCCTAA

>ClDof1

CAAAACATTCAATCCAAATCTGATGCATTCAAATGCCCTAGATGCCATTC

TCTCCACACCAAATTTTGCTATTACAATAATTACAATTACTCTCAACCTC

GCCACCTTTGCAAGACCTGTTGCCGCTACTGGACCCTCGGCGGCCTCCTC

TGCAACGTCTCTATTGGTGGCGGAACTCACAGATCGAAGAAGAATTCCAA

AGCCAAGACTGCGATTGTTAGAGATTCGCCGCCTTCGAACTCTGCTGTTC

TTCGTTCTGATTTAGAAATGTTTTCGATTATGTTGAAGTTGAATCAGGCG

GGTGATTGGTGCGGTTGGATTGAGAATGGAAGGGAATTAGATGGTGGGAT

TGTGGGTGCGGCAAGGGATTCGACGGTGGAGGGATCTGGATCGTCGGATC

ATCTGGCGACAAGTGGTGGAAATGAAGGAATGGTTGAGATTTCTGTGGCG

TCACCGTCGATGGAGGTTCCTGGTTCAAATTCTATGAATTAA

>ClDof9

ATGGGTTTGAGTTCAAAACAGGTTTCAATCGATGGATTTGATTGGAGCAA

GGCGCTAATGCAAGCACAGAAACTGGAACTTCCAAAATTGGCTCCGAGCT

CCGGCGTGAAACGATCACAACACCAAAATCAGAATCAAATTCAGCCACAG

ATAGAGCAATTGAAATGCCCCAGATGCGATTCAACCAACACAAAATTCTG

TTACTACAACAATTACAACAAATCTCAGCCTCGCCATTTTTGCAGAGCTT

GTAAACGCCATTGGACCAAAGGTGGAACTCTACGAAACGTCCCTGTTGGT

GGCGGCCGTAAAAACAAGCGCCTCAAAAAAAAACCAACCCCCAAATCCAC

AAAATCTTCCTCCTCCGCCGCCGCCGACGTGATTAATCCCCAAATGGATG

TTCATCATTTTCAAAACCTTCCTCTGTATCAGGGGCTAATTTTTTCCCCG

CCGTCGTCCAGCAATTGGGCGGAATGCGAAAATTTCACCACAAATTACGG

GATTCTAAATTCTCAACCCCCCGATTTCTCGGCCGTTTCAACAACCACCT

CAACACATTCTCCAATGTCCCCAAAATTTAATAACTATTCAGATCAAGAA

TTGAAACCCACAGAAACAGAGCAACCGGCCAACAGCACCAGTACTCATCA

TCCATGGCAGCTTCCATCCACCGCCTGCGGCGTCGCCGACATGTCCAACA

GCTACTGGAGCTGGGACGACATCAACACCTTCGCCGCAACTGATCTCAAC

ATCCCATGGGACGACGACCATGACATCAAACCTTGA

>ClDof25

ATGGAAGACATAAACCCTAATTCAAGCACAAGATCATCACCATCATCATC

ATCCATATTGGAGATCAAGAAATCAACAAAGCCTCCTAAAGATCAATTGA

ACTGCCCAAGATGCAAGTCCAACAACACAAAATTTTGTTATTACAACAAT

TACAGCCTTACACAACCAAGATACTTTTGCAAGTCTTGTAGAAGATATTG

GACTGAAGGTGGGTCCCTCAGAAATGTCCCTATTGGAGGAGGCTCAAGAA

AGAACAGATCAAAATCAACAACAACAATAACAATAATGTCTAATTCTTCA

GCTCCTTCTTCTTCATCTTCATCTTCATCTGATCATTTCAACAACAACAA

CCCAAAATTACCAAATTACCCTGCATCTCAATTTTCTTCACAGAACCCAG

TTGGAAAGGATCTTAACCTTGCTTTCCCTTGTGCCATGTCTCATTATGCT

GATCATCAGCTCTCTAAGGTTGAAAATGTCAACAATAGTAATTATAATTG

TGGAATTGGTTTTAGGGGTTTGAGTTCTTTTATCCCAAATCTAATGCCAA

ATTCCAATAACAATAATACTAATTTGGGGATTAATAGTAATGGCCAATAC

CCAACTGGGTTTTCTTTGCCTGATCTTGATGAGTTTAAGCCAAGTTTGGG

TATTAATTCTGTTGATGGGTTTGGAGATGGAAGAAATTTGCTTCCTTTTC

AAGAATTGAACCAACAATCTGCTGCCCCAAATGAAGGTGATCATCAAAAT

GAGAGGCAACAAGGGAATAACATTTCAACTGGATATTGGAGTGGGATGCT

TGAGGATGAATCATTTTAA

>ClDof24

ATGGCTGAAGTTGAAATTGGAGATCCAATCATAAAGTTGTTTGGGAAGAC

CATTGCTTTGCCACTGAACCATCTGGATTTACCTTCTGATTCCAAATTTC

TTTCTTCAGAAACCTCTGTACTTAAAGCTGGCGAAAAGGTAGGAATTTTT

ATTTTTTATTTCAGACTCTCTCTCAATTCTGTCTCTGCTTTTTGCCAGTC

AAGTTCTAGACTTGATTGGGTTTCTTTTTTCTATCTGGGTTTTGAATATT

GGATAGTGAGATTCAAACGAATTTTGGGTAAGTAGTGTTTAATCCTACGT

TACTCTGTTTTTAAGACTTCAAATTCTTGACTTTCTTCCACAGGAAACTT

CATGTGAAGCATTGGCAACAGAAAAACGAGGGATTAGCAGTGGAAACCAA

ATCACCGATCAAACAACATCAGGCATGAGTGAGAACCCATCAGAAGAAAG

AGAAATTTCATCTCCAAAAGCTTCCAAAAATGAAGAACAGAGTGAAACAA

GTATCTCCCCGGACAACAAAACCTCAAAAAAACCAGACAAAATTCTTCCT

TGCCCTCGATGTAACAGCATGGACACTAAATTCTGTTACTACAACAACTA

CAATGTCAATCAACCTCGTCATTTTTGCAAGAATTGCCAAAGATATTGGA

CTGCTGGTGGGACCATGAGGAACGTTCCAGTCGGTGCCGGCCGGCGGAAG

AACAAAAGCTCCTCATCTTCACATTTTCGTCAGCTAATAATTCCCGACGG

TGGAATTCATCGCCATCAATTTCTAGGGAACAATGGAACCTTTCTCACTT

TCACCTCAGATTCTTCCATTTCCGACTCACAGAACTGTAACCCAAGTGGG

TTTCTCATTAGTACAGAAAATGGAGATGATCATTCCAGTAAATCTTCCAT

TACAGCTTCAAATTCCTCGGAGAAAGATGGTAAAATGATCTCACAGCAAT

CAGTAGTGAAAAACGTTTTGCCTTTTCAACCTCAATTACAGACATTTACA

GGACTTTCATGGCCTTATTCTTCAACGGCTCCACTGCCGTACTACCCGCC

GGCGTTTCCAGTGTCGTATTACCCTGCACTGCCGTACTGGGGCTGCACAG

CGCCGCCGTCTTGGACAGTAATTAATCACAACGGTCAAAATTCGTTTACT

AATTCCTCTGTTTTGGGGAAACGTTTAAGAGATGGGAAATTGGTTAGAAG

TCCAGCAAATTCAGAGTGTGAAGAGATTGTTAAACAGAGGAATTGTGAAG

AACCCTGTTTCTGGAATCCAAAGACAATGAAAGTGGATGATCCAAATGAA

GCTGCAAAGAGTTGTATATGGTCAACACTTGGAATTAAGAATGAAAAAGC

TGGTGCAAGTACAATCAATGGAGGTAGCCTTTTTGTCTCTTTACAACAAT

CTGCAAAGGGAAAGGAAGAAAACCACATTGAAAGTTGTTCGCTTTTACAG

GCAAATCCAGCTGCTTTTTCTCGGGCTTTGAAATTCCGTGAGATTGCTTA

G

>ClDof10

ATGCAATATCAGGATCTGAGATCGAAGAGTTTACAAGTGCAAGATCAAGT

TCAACCACAACCTCAAAAATGTCCTCGTTGTGATTCTCTCAATACTAAGT

TTTGTTATTACAATAATTACAGTTTGTCTCAGCCTCGTTATCTTTGCAAG

GCCTGTAGAAGGTACTGGACTCAAGGTGGCATTCTCCGAAACGTCCCCGT

CGGCGGCGGTTGTCGGAAGGGTAAACGACCGAAGCAATCTTCACAATCAT

CCAATAGTATTGTAAAGTCAGTACAACCGTCAACGTCGCTGCCACCACCA

CCGCAGATAGTCCCGTTGAGTTTAACAACAACAACTACAGAACACGTAAT

CTTTTCGGGTACTCCTGTTATCACTCCGTCAACGTTTTTCAATCCGGGTG

AGTAACAGGAGTAGAGCTAGAAATTTTCATAGAGGAAGGATAAAACTTTA

ACCTTGATTTTGATTATAATTAGATTTTCCTTCCCCTTTTTTTCATGGAT

TAGTCTTTGAATAAAGATCCAATTTTTATGATGGGGTTTTTGTTGGAGCA

GGTGGGGAATTGTTGTCATCTTCAAGCTGGATTAATTCATTTGGATCATC

ACAAGGGCAGTCTCAAATTCAACCACCAGAAATAATCTATGAAATGATGG

ATCAGTCGTCGGGAAAGGCCGCCACGAGTTCATCTTCTACAGTGGCGGGA

TGGACAGAAAGCTACATCAACAATTCCATTTCTAACCATATGGCCGTCGC

CGGAGATGCTATTGTCTGGCCAGCTGGCAATAATAACGCTGCCAACGTCA

CCACCGCTGCCGCCACCACCACAATTAACCAGTGGCCTGATTATATGCCA

GGATTTTGTCCTCCTCCATGA

>ClDof13

ATGGCTCACTCTTCTCTTCCAATCTTCTTAGATCCTCCAAATTGGCATCA

AGTAAGTTTTTTTTTTTTTTGAGGTTCAATTTAATTTCATTTGCAAAGTT

ATGGAAAGAAAAGAAAAAAGAAGAATGGGAGATTTTGTTTCTTTTTTTTT

AAATTTATTCTATTCTTGAATATACCCAATTTCAAAATGTTTATTAATTT

AATTTTATTGTATTAGCCGAATCAGCCAGCAGCTGCAGCAACAAATGATG

TTCATCAAGACCCACGTCAGCTTCCGGCAGCATTTCTACAACCGCCGCCG

CCGCCAACGGCAGGTCATGGAGGAGGCGGAATCCGGCCGGGTTCTATGGC

GTATAGAGCCCGGCTGGCGATGATTCCCCAGCCGGAGGCAGCACTAAAAT

GCCCTCGTTGTGATTCTACAAACACCAAATTTTGCTATTTCAACAATTAC

AGCCTTTCTCAGCCGCGCCACTTCTGTAAAGCTTGCCGCCGGTACTGGAC

CCGCGGCGGCGCTCTTAGAAACGTCCCCGTCGGTGGTGGCTTTCGCAAGA

ACAAGAAGAAGAAGAAATCTAACCCGTCTAAGTCCCCCACTGCCTCTCAA

TCTCAAATGGGTAACCTTCAAAATTTCCTTTTTTTTTTTTTTTTTTTTTT

TTTTTTTTTTTTTTTTTTTTTTTATTTAATTATTTAAAAAACATCACTGA

CCCGACCAAAATTAGTTTAAGCTGATAAGTTGTATCAAACACAGAATTCA

ACCAATAATCTATAATGCCAAAGGAATTCTAACTTTAAAACTTTTATTTA

TTTAAAAATATTCTTATCCAAAAGTTTTGTCACCAGACGAGTTCTTAACT

TTTCTTTTCGATTAATGAAAATATCCCTAATTAGTACATAAATCAAAACA

ATTTCACCTCGATTTTATTAGGATATTATTTTATTTTCATTTTTTTGAGT

TCATTGGCATAAATATAAGATAAAAGACATAAATGTTTGTTTCATAAGAC

GTCAATGGTTAAATTAGGTTGCATATATATATCTTATTTAACTTAGCTAT

ATTTGAATTGATTTCAATTTCGTATTTCTCCAATCTTTAAACGAAAATTA

AATCACTGCTAAATTAAGAAGGGTTAAAATTACAAAGTTATATTTAATAA

TAATAATAATAATTTTTTTTTTTTTTTTTTAAGGTAATTCAAGATCAATA

ATGTCAAGAAGCTGCAACAACATGGAGAGCAGTAGTACAATGAGGGATTT

CCCATCACATTCTTCTTTGCCTTCATTAAATATCCTTCCATCTCTTCAAC

AACATTTTTCTCGTAATTTTGGAAACAACTTCCCCGGAATTCACATCAAT

AATTCCGCAGTCGCCAGAGAATACGACGTCGAATGGCAACAACAACAACA

ACCATTCATCGTGGCCGGACTAGAATCTCCGACGACTGCGACAACAACTG

CCGCAGCATACACACAAGCTGAAGTCAACAACAACGGCAACAACAACAAC

TTTGTTGCTAACTCATTACAACAAGCAGCTGGTTTAGTACAATACAATAA

TAACTGTCATCAACAACTGCAAAATCTGACACCCTTTTCCAGAATTCCTA

TGAAAAATGAAGAACAAAACCAAGAACAAGGAATATTATTATCAAATTTC

TTACGACCAAATGATCACAACAGCCAGTTTTGGGGAAGTAATCATCAAAA

TTCATGGACGGATCATTTGTCAGCTCTCAGTTCTTCTTCAAGCAGCCATC

TTTTGTAA

>ClDof36

ATGAGTATGACTTAAAATGCATGTAAATCACTAATCTCTTTCCTCTTAAT

CTATGAATTAGCTAAACACAGTGATGAGAGAGAATATGAAAGGATCAAAA

GAAGCTTATGGACTAAGAAACAACATCTCTTATGCCCTAAAAAAGCAATA

GAGAACAAAAGGAAAGGAGGGAATGACAACTACTATTTTTTTCTTTTTTA

TATATATATAAAAGTTCTTCACTCCTTTAGTTTTGCTCCCTTCTTCCTCT

TCCTTCTTCCTCCCTCTCTTTTTCTCTCTCTTCTCTTGATGTGATTCTCC

ACTATTTATCTCTCCCCAAAACCCATCAACCTTTACAAAAGAGAAAGAGA

ACCCAGAAAAACACCATGAATTTTTCCTCCATTCTCCTGCTTATCTTGAT

CCTTCCAACTGGCAACAGGTAAGGGTTCCTTCATTTCTTTTTCTTTTTCT

TTTTCTTTTCTCTTTGGATTTTTTTTTTTTTTTCCTTTGCTAGTAAAGCT

AAAAAAACAATGGAACCCCCAAGAGAAAAAGATAAATATTCCCCTATTTT

GCTTTCCATATACCTTCTGTTGTTGTTTTTGAATATACTAGAAATATTTG

ATTTCATTCCCCTTCAAATAATTTTACTCATCAGCAAGTCACCCATCAAG

TGGGAAGCAGTAGTAGCACTGGAGTTAGTTCTCAGCTTCTACCACCGCCG

CTGCCGCCGCCTCCACCGCCTCCTCCCCCACCACCCCATGGCGTCGGCGG

TGCCAGTTCGATCCGCCCTGGTTCGATGGCCGAGCGAGCTCGAATGGCTA

ATATTCCGATGCCCGAGGCAGCATTGAAATGCCCAAGATGTGAATCAACC

AATACTAAGTTCTGTTACTTCAACAATTACAGCCTCACTCAGCCTCGCCA

CTTTTGCAAGACTTGTAGAAGGTAAATTCAAAAGATACAAAAATAATCTT

TATATATTCTCTAATATCTTCTTTTATAACTATATATACATAGAAGATTA

AGATTATTCCACCCCTAAAAGGGTTGGAACTGGACAAGGACAACTTAGCT

TGTTACAAAATTCAAAAAGTATCTATAAGATATTTAAGAAATCATAACTC

CTCTCTACTATATGTTGATTTTTAAATAAATTTTCCGCTCCGCTCTGGAA

GGTACTGGACCAGGGGTGGGGCCCTACGGAACGTCCCGGTTGGGGGTGGG

TGCCGGAGGAACAAAAGAAGCAAAGGGAGCAGTTCGAAGTCGCCACCGGT

CAGCTCGGACCGACAACAAACCAGCGGGTCAGGCAATTCTTCATCAAGTG

CAATAGCTTCCAACAACAGTGGAGGCCTTTCCCCACAGATCCCACCACTC

GGCCGGTTCATGGCTCCTCTCCATCAACAGCTCAGTGACTTCGACATAGG

TGGTTTCAGCTACGGGGGTGGCCTCTCAGCTGCTGCCACCGCCACCGGTG

ACCTGAGTTTCCAATTGGGCAACACTACTTTAGCTGGAGGAACAAGCATT

GGATCTCTCTTGGGATTTGACCAACAATGGAGGCTCCAGCAACAACCTCC

CCAATTTCCCTTCTTGTCCGGGTTAGACCCTTTCGACGGCGGTAGCAGTA

GCGGCGGAGAAGGACCCGGACCCGGACCCGGCTGGCAAATGAGGCCAAAA

TTACCATCAAGTTCAAGGAATGTAAGCCAAATGGGGAATTCAGTGAAAAT

GGAAGAAACCCCAGATCAAGTTAATAATGCTGGAAGACAATTTCTTGGGA

ATGAACAATATTGGAGCAGTGGATCAATGGCATGGTCAGATCTTTCTGGT

TTCAGTTCTTCATCTTCAACAAGAAACCCATTATAG

>ClDof4

ATGGATACTGCTCAATGGCCACAGGTAATCTTCTTCTTCTTCTTCTTCTT

CTTCTTCTTCTTCTTCTTTATGATCTCTTTTCTTTGTGGGGTTTTGGTTT

TTTTGCTATGGCTATCACAAACAAAACAAAAACAAAGACAATACTTTTAA

CTTTCTCTTTAACTTCTATGGGTTTTTTTTTTTATCTCCACTTCTCCCAA

AATATTCAGTGACAAAACTGATACTTTTTCTTCAGCACATTTGGCATTTG

TCCTTTCTCTTTATTTTGTGTCAAAAGAAAAAGGGTTTTCAGTTCTTTTT

TCCTTTTTTCTATTTAAAAAAAGAATTCTATCTTTTTCTCTCTTTTTCTC

TTTGGATCAGATAACCTTGAAAACTGTTCTAAAGCAGCTTTAGTTGAATT

TGTTGTTTTGTTATGTCTGTCTTTCTTTTACCTTAGAATATATAATTAAT

CTTTTTTTTTTCTTTTCCCCCCATCTTTTATCATCTTGGGGTTTTGTTTT

GATATATCTTTTGATGTCTTCTGGTTGCTTGTGTGTAAATTATATTGATT

TTTTTTTTAGGAGATTGTGGTGAAGCCAATTGAAGAAATTGTGACAAACA

CATGCCCAAAACCAAGTGTTTCAATTTTAGAGAGGAAGATTAGACCTCAA

AAGGAACAAGCTTTGAATTGTCCAAGGTGTAATTCAACCAATACCAAGTT

TTGTTATTACAATAATTATAGCCTTACACAACCAAGATACTTTTGCAAAA

CTTGTAGAAGGTATTGGACTGAAGGTGGCTCCTTAAGAAACATTCCTGTT

GGTGGAGGCTCAAGGAAGAACAAAAGACCTTCTTCTTCATCATCATCTAC

ATCTCCCACACCTTCTTCATCTCAACCTATTCCCAAGAAAATTCTTGATC

TCCCTCAAAACCCTAAATCCACCCAAGATCTCAACTTGTTCTTCCCACCC

TCGAACCATCAAGATCATCATCATTTCATCACCAATAGCCACCCCGAAAT

CGTTGCCCCTGCGATTGCCAGTTCCTCATCAGCCTCTACTGTTGCGACGG

CGGCGGTGTCGTCGTCGCCTTCGGCCCCATCGCATCATCATCCGCTTTCG

GCAATGGAGCTTCTCGCCGGGATGGCGAACAACAACAACAACAATTCTAG

GGGTTTGAATTGTTTCTTTCCAATGTCGTCTGTTGTTGTCCCTGATCCAG

CTGGCTCCCTTTACACGGTAGGGTTTCCCGATCTCCATGATCAGTTCAAG

TCAAACCTCGGATTATTCTCTCTTGATGGCTACAGCGTTGTCCCGACCGA

GGGAAGCAAGGGTGGGGGGAGGCTTATGCAGCTGCCATTTGAGGATTTGA

AGCAAGGGTCTAATGGAGGAGTTGAACAAGGCAAAGAGCAAGGAGAGCAT

AATTCAAGTGGGTATTGGAATGGAATGTTGGGTGGAGGATCATGGTAA

>ClDof34

ATGACGACTTCTACTACAGAGGATGTCATCTCCGCCCCACATTTCAAGGA

TCTCGCCATTAAACTCTTCGGCCGGACCATCCCTTTGCCGGAATCCCAGA

TTTCCACCGCCCCTCTTCAAAATCCGGTAACTATTTCATTTTCCCCTTCC

TTGATTCTCTCTCCATTTTCATGGGTTCTTGCTTTCTGGGTTTAATCTGA

AAAATCCCTTTACTGTGTTGTTTTGTGGGACATCGGGAAAATTATTGAAA

TGCCCATTTCACTGTCTTATTCATATTCTTCTTCTTCCATTGAGTTGTCT

CTGTCCCCTTTTCTACTCTCATTTCAACTCTTCATTTCATTGTGTGTGTG

TGTTTTTTTTGTTGTTTTGGTTCTCTAAGTATTTGTGTTGGGGCCCTTAT

TGGGGCTTAACTTCAATTACATGGTTTTGTTTAGATTTATTCATGTAATG

TTTTAGTGGCCTTGGTTGACTTTACTGTTCCTTCCCAACTTTATGTTTTT

GGAAGAAGTTAATTAGGAGAACTGTTTTTAACCAAATTAGAATGTGGAAT

TCAGGTGGGTTTTTGGGTTAGTTTGTATTAGATCCATTGATGGTTAAGTG

AAGTTCTTTTTTGAACATTTGAAGCTGCTGAAAATGTTTGATGAAGATTG

AGTTCTGTTTATTTATCTCCTGTTTCCTTTTATCTTGTCCTTTATGTTGC

ATATTGTACTATCTTTCTTACTTGAATGAATGTAATTTTTACCTTCTTTG

GAAAGGGTTTGTGAAGTCTTGTTTTGTGAAGTCCCTCCTTCAAGTAGGAT

TTGATGCATTTCTTGGCTTCTTTTCTTATGTACGACGAATGCGTTGACCG

TGTACTTCTCTATGTATTTCCGACTGTTGTTAGAGTGTCAATTTGTTGCA

TATTGTTTTCAGGATGCTTGCAACAATTTGAAAAAAGCTGAGCAGTCTGT

CTTGGGTGCAGAAGACTCTTGCCCATCTGAGAGGTCTTCGGTTTTGGTAG

GGGATAATGAAGAAAACCAAGCTTCGAACGTGACGTTGAACAAGGGAGAA

TTGGAACTGCCTTTGAAAGAGGAGCAGGCGGACTGTAATGGCACGGATCA

AGAAAGAGTTTTTAAGAAGCCAGACAAAATCATTCCTTGTCCTAGATGTA

ATAGTTTGGAAACAAAATTCTGTTACTTCAACAACTACAATGTCAATCAA

CCTAGGCATTTCTGCAAGAACTGCCAAAGATATTGGACTGCTGGTGGGAC

AATGAGAAACGTTCCTATCGGTGCTGGACGGCGAAGGAACAAGCAGTTGG

CATCTCAATACCGACAGATAATAGTATCCTCTGAGGGAGTTGCGACAACC

CGGTTGGAAACTTCGGATACGACGAATCACCATCAACTACTCTCAGGCGT

AGAGTCTCCTCCTACTTTGAGGCCTTCCACTGGAAATAGTACTGTCCTAA

AGTTTGGACCCGAAGCACCACTTTGCGAATCTATGGAGACTGTGCTTAGT

CTTGGAGATCAGAAGAGATCCATTGAGATTGGCTCTGCATATTGTGGAGA

CAGTCCTGAAGAACCATCCTCGTGCGGATCGTCCATGACAACTACTAGTA

TTCGTGGAAACGAATTGCCTAAAACCATTGTTGAGAGGCCAGAGGCAGTC

AGATTATCCAACTCTAGTAGCGATATCGCTGCATCGAATACAGTTCATTG

TTATCCGGTTCCACAATTGGTTTTTCCATTGAATCAGGGCAGCAACGGGA

TAATTTCATCAGCAATGGCACAAAGTTCAGATAGCACAAGCGTAGCAAAT

ACTAGCAGTCACCCCAATCCACCAGTTCAATGGCTCCCCGCAACAATGCT

GGCAGTCCCAGGCTTTTGCACTCCAAGCCTTCCATTACAATTTGTGCCAG

CTTCGTGTTGGGGTTGTACGCCCGTATGGACCGCTACTGGTGCCGGGAAT

TTAACCGTTGTTCCTTCTGATATCTGTGCATCTCAAACACCCCCAACCGC

CATTGGTAGCTGCCCTACAAGTTCGTCGCCTACACTAGGGAAGCATTTGA

GAGATGCAAACAGCTTAACAGAGGACGAGAAGTCAGAGAAATGCGTCGTG

GTTCCAAAGACATTACGAGTCGATAACCCAAGTGAGGCTTCAAGGAGTCC

TATATGGACGACATTTGGAATTCGTCCATATCCAAAGGAAACCATTTCTA

AAGGTAGTGTTTTTGAAACTTCAGAAACTACTAATGCAGATAGCAAAGGC

CATTTTAGGGATGCTCCTCATATCTTGGAAGCAAAAACTGGGAGCTTTTA

CTCTCTTTTATAG

>ClDof14

ATGCTGGCCATTTCATGTCCAAAAGTACAGCAAGAAAACAGAAAGCCAAT

GAGGCCACAGCCAGAGCAAGCTCTGAGATGCCCAAGATGTGATTCAACCA

ATACCAAGTTCTGTTACTACAATAACTACAGTCTTTCCCAGCCAAGGTAC

TTTTGCAAGTCTTGTAGAAGGTATTGGACTAAAGGGGGTACTCTAAGAAA

TGTTCCTGTCGGTGGAGGCTGTAGGAAGAACAAAAGACCATCACCTTCTA

CTTCATCTTCTTCATCTCCTTCTTCAAAGAGATCGCATGATCAAGCTGCT

AATTCTAGCCGTCTAATGCTCACTGATACTCATCTTCCATCTTTAGCCTT

TGAGTCCACTGACCTAAGTTTGGCATTTGCTAAGCTCCAACATCAATCAA

ATGGGCAGATAGGTCTCTTTGATACAACCCACAACAACTTTCATAACTTG

TACTCTGGGTTTGGTACTGGGAGTTCTGGGGAGGTTGAAAATGGTGGATT

TCCTCATTTTGAAAATTATAACGGCAGTGGTGTTGCATCTACAGCCACTG

CTACAGCGATGAAGCAAGAGTTGGTTTGCAATGGAGATCAAAATAATATG

GTTTTGTTGGGATTTCCATGGCAGTTCAACAATAATGGAGATGGAAATTA

CATGGGTGATCTTGATGCTGGAAGAGAGAGCTGGAATTTCAATGGAAATG

GAATAGCTTCATCTTGGCATGGACTTCTCAACAGCCCTCTAATGTAA

>ClDof15

ATGGATTTCTCTTCTGTTCCAATCTATTTAGATCCTCCTCCCAATTGGCA

CCAGGTAATAATAACCCTTTCTTTCTTCCTTGTTCTTCACTTATTTACAA

AGAATTATTCCCTATTCTCTTCTTTTGTAATCTCCCCTTGAATATCCCTC

TTATATCTCAACAATTTTCTTAATTGGATTTTTGCTTTTTGTGGTTTTGC

AGCAATCAAATCATCATCATCACCACCATCCTCAGATAAGTAGTAATAAT

AATGGATCTCCTCAACACCAACACCACCTTCTTTCATTGCCTACTCAGCA

ACTCTCCTCCTCACCGTTGTCTCATGTCGGTGGTGGCGGTGGTGTTAGCT

CAATCCGGCGTGGTTCGATGGCGGACCGAGCTCGGATGGCTAACGTACCA

CTACCGGAGACAGCACTCAAGTGTCCTCGATGTGATTCAACCAACACGAA

GTTTTGTTACTTTAATAACTATAGTCTCTCTCAGCCTCGCCACTTTTGTA

AGAGCTGCCGCCGATATTGGACTCGTGGGGGTGCCCTAAGGAACGTTCCA

GTTGGGGGCGGTTGTCGCCGGAATAAGAAGAATAAAACTAGACGCTCGAA

GTCTCCGGCTGCAGCTCCGGCCGGAAATGAAACTCAAGTGATGAATAACA

ATTCAAACTCACCCACCACTACTACAATACCCTTGCACAGTAGTGCTGAG

AATATTATTGGTCATTTACAGCCTCAACATCCTCACCTTTCATTCATGGC

TTCATTGAATAATTTTTCTCGATACGGGACAACGAGTTTAGGGTTGAATT

TCAACGAGATCCAGGCTCAAGGTAACGATATCGGCAACGGCGCCCTTCTG

AATCATCATCTGTGGCGTGGTTCCCAAAACTTTCCTCTCTCAGGTGGCCT

AGAAACTCCACCCGGGTTGTACCCGTTTCAAATTAGCGGCGGGGACGGCG

ACGACAACACAACAAATTCAATCCTAACACCAAATTCAAGGGCAACCCAT

TTGCCTCCAGTGAAGATTGAAGAAACCCAAGTGCTGAATTTGTTGAAATC

ATCCAATTTGGGTATCAATAATTCTGAAAACAATCAATTTTGGAGCAATG

GAAATGGTTGGACAGATCTTTCAGCTATCAGTTCTACTTCAAGTGGCCAT

ATTTCTTGTGACTTCAATCATTAG

>ClDof6

ATGCCTTCCGACGCCGGCGACCACCGCAAGGCTGCCACCAAGCCCCTTGG

CTCCGGCGGTGGTGTTTGCCCTCCGCCGGAGCAAGAGCATCTTCCTTGCC

CACGTTGTGATTCCACTAATACTAAGTTCTGTTACTATAATAACTATAAT

TTCTCTCAGCCCAGACATTTTTGTAAGTCCTGTCGTCGTTATTGGACTCA

CGGTGGGACTCTTCGTGACATTCCCGTTGGCGGTGGCAGTCGGAAGAATG

CTAAACGTTCTCGTACTTGTATTCCGTCGTCTGCGGCGGTTGCTTCATCT

TCATCTGGGTCGTTGCCTAACTCTATTTCTCGCTTGGATCATCATTCGTT

ACCGGCGACTCCGGTGTTGGTGCCTCTCGTCAGTGGCCACGGTGGTGGCG

TCGGAGGGGACTTGAAGGTGGGCGGTGGGAATATGTGTGGGAGCTTTACG

TCGTTGTTGAACACGCACGCGCCTGGGTTTTGGGGTTTGGGTGGGTTCGG

GCTTGGGCTTGGGTCGGGGTTCGAGGATGTGGGCTATGGGGCGGCTAGTC

CTAGGGTGGCTTGGCCGTTTCTTGGACTGGGCGACGGAGGCTCTGGGGTC

GGCGGCCACGGTGGAACGCCGAACGCGTGGCAGTTTGAGAATGGGGACGC

CGCCGGATTTGTTGGGGCTGCGGAATGTTTGTCTTGGCCGGAATTGGCAA

TTTCCACTCCGGGGAATGGGCTCAAATGA

>ClDof7

ATGATCCAAGAACTATTTGGTGTATCAGGGCTTTTAGCTGCTGCTGGAGA

CACCAAAATCTCCATCAATGGTTCAATTCTAGATTCTTCCCCTTCCACTT

CCCTTTCTGCCTCTGCCACCGCCGCCACCACTGCCACAATCACCACCACC

ACCACCGCCGCCGCAAGCACTGCCAACGCCACATCAACAACCTCCTCAAT

TTCTGAAGGCCAAAACCTGCGTTGCCCCAGATGTGATTCTTCAAACACTA

AATTCTGTTACTACAACAACTACAACCTCACTCAACCTCGTCACTTCTGT

AAGACATGTCGCCGTTATTGGACCAAAGGTGGGGCCCTCAGGAACGTTCC

CATCGGTGGTGGGTGTCGGAAGAACAAGAGCACCGCCGCGACGGTGGCTG

CTGCTGTAGGGAAGTCTGCAGCTGGAAAAATGAAAACTTTATCGTCTGAG

ATATTGGGTCGGGTCGGATTCCGAAATGGGTCTGCGTTGGATCATGAAAT

AATATCATCGCCCAAGCAAATTCTGTGGGGGTCGCCGCAAAATTCTCACC

TTTTGGCTATTTTGAGATCCGCCACCCAAAACCCTAACCCTAATAATCTC

GCCGCTTCCCACGTCATTAACAACGACCCACCGCCGTCCACGCCGTTTCA

TGCTCGGACGATGGGTTTCGATGATCCGGTGGCAGCCACTCATATTTCTT

CTCTGGGGCTCTGCAGCTCTTACTGGAGAAACAATCAGACTCAAGTTCAT

CATCAACAAAATGGGTATCCTCACGGCGGCGGAGATCAGGTCCACGGCGG

CGCCGGTGGGATTAAGGAGCTCTATCAGAAGATTAAATCGTCGTCGAACA

ATTATTTCACCGACGGCCATCAGGGATCGGTGGGAGTAACGAGTGTGGGT

ACAACGACTACGGCGGCGATTTTGGAAGCGGCTCCGGTGGGCGGCGGCGA

AACGACAGGTTTCTGGAATCCAGCTTTTTCTTGGTCTGATGTTCATGCTA

GTGCTAATGGAGCATATCCTTAA

>ClDof22

ATGGAGGCGATGATGGAGTCCGGCAGCGGCGGCGGAGGCGGCGGCGGAGG

GTTGAAGGGAAAGGGAAGGCCGCAAGAGCAATTGAACTGCCCAAGGTGCA

AATCAAGCAACACAAAATTCTGTTATTACAATAATTATAGTCTCACACAG

CCGAGGTATTTTTGCAAATCTTGCCGCCGGTATTGGACGGAGGGCGGTTC

TCTTAGGAACATCCCTGTCGGAGGAGGATCCCGGAAGAATAGGAAACCGC

CCGGTCCGGTTGTTGGCGGGTCGGGTTCGGCTCACCACGCGCCGCCGCTG

CCGCAGGTGTATCAAGCCCATGATCTGAATTTGGGGTTTGCGACGGCGGA

GGCCACGGCGACGACGATGGGGATGGAAAACGGTGGCCACGGAGGATTTG

GATGTTACATACCGAATTTGATGCCGTATTCCGCACGTGACACGGCAGCG

GTGGAAGAGAATTCCGGTAATAATGGGTATTGGAATGGGATGTTCGGTGG

AGGGCCGCCGTGGTAG

>ClDof32

ATGGTTTTCTCTTCAGTTCCTGTGTTTCTTGATCCACCCAATTGGCCTTC

TCAGGTATGGTGTTTAATTTCTTTTTCTTTTACTCTATAGCTTCTTCCCT

ACAAAAGAAAGAAATCAAAAAGAAATTTTATTCCCTATTCTTCTTTATTT

CATATCTATCTTTACTTGAATATACCAAACATTTTTGATTTGGGATTGTA

TGCTTTTTTGATCTCTCTCAATAGCAACCAACTCAGCTACAAGGAAGTGG

CTGTGAGACAAATAATGCTCAACACCTTCCACCTCCGGCACCGCCAACTA

GCGGTGGCGGCGGTAGTGGACCTGGCTCGATTAGACCAGGGTCAATGACG

GATCGAGCTAGGTTGGCGAAGATACCACAACCGGAAGCCGGGTTAAAATG

TCCTAGATGCGAATCAACAAACACAAAGTTTTGTTACTTCAACAACTACA

GTCTCACTCAACCTCGCCACTTTTGCAAGACGTGCCGACGGTACTGGACG

AGAGGTGGAGCGATGAGGAACGTACCGGTTGGAGGCGGGTGTCGGCGGTC

GAGCAAGAGATCAAGCAAAGGAGGAAGCAATAGGTCAAAATCTCCAGGGG

GTTCAAGCACTTCAACAAGCACAGTGTCATCAAATAGTTGCACAACAGAC

ATAATTAGCCAACAACTGGGTCATCATCCTCCTCCTCCAACAACCCATTT

GCCCTTTTTTTCTCAAAATTTACATCATCTTAGTGATTTTGGGAACCTTG

GTTTAAACTTTGAAGCCCTTCAAATTCCTTCACTCCCATCATCAGCTGGA

GGAGTTGAATCCTCAATTTTATCAAGTGGAATTACAGATCATCATCAATG

GAAAATCCCCTTTTTTGCAAATTTACATCAACAAAATGGGATGTATTCAA

ATTTCATGGCTGATCAAGATCATCATCATCATCAACAAGGAGCCGATTTT

TCAAATTACCAGCGGTTGTCAAAGCCATTATTGGAGAGTGGATTCAATAC

TACTACTACTACAAATCATCATCAATTGGAGAATATTAATAATAATAATA

ATAATAGCATGAAAATCTTGGAGGAGACACAAGGGATTAACAATTTGTCA

AGAAATTTTCTTGGAATTCAACCAAATGATGATCAATTCTGGAATTCTAG

TACTACAACAACAACAACACCTGGGAATATTGCATGGAGTAGTGATCAAA

TTTCAGATCATTTCAATTCAACTTCTTCCACTACTCATCTCTTGTAA

>ClDof27

ATGCTGGAAATTAAAGACCCTGCCATTAAAATCTTCGGCAAGGAGATTCA

ACTTCCGGCCGATTGTGAGGTTTCGTTGATCGAAAGCGATGATTCCGTTT

CTACTTCCGATAAAGAAAGCGGAGATGGAGCACTACAAAAGGTACACACA

GTTCACTCGATCAAGATTTTGTATACTCTCTTTTTTCCTATTTCTGAACT

ACGCTACTCTGGCATGGCGGTTGCCGTGTTGACTAGTTTATATGTTCTTC

AGATTTTGTTGTTTTCCGGTTTTTGATTGGTGGTTTGTAATTTGTTAACA

CCTTCTCGAGAAGATTTTGAACTATTTCGTCGCTAGGTTATCCTTATCTG

CGACTTTTGGGGTTCCCAATGGATATTAATTTGGTTTTCATGTGTGGAGT

TGGGGAAAATTGAATTCCGCAATTCGTTTTGGATTCAAATAAGGAACCTT

GTGAAACGGCAAATCGCTACAAGTTTTGCGATAATCAAGCTAGAGTTCGC

AGTTCTTATTGTTTTTGCCATTTTCTTTGAAATATCCAATTCCACGCAAT

TCGTGGTTAGATGTTCTTTATTGTCTTCAAATTTATATGGATTTTCTCAT

GTTTCAATGGATTGATTTCCAAACTTTGTTTGCTGTTTGACTTCTTCACA

CTTTGACAATGAACCAGTATAAAAAAGAGAAAAAGAAGAAGAAAATATTG

GGAAAATACTCACAATATGAAAACAAGTGTGAATTAAATATCATTGCTCT

TAGTTCTTCTAGGTACTGCCAACTAGTTTCCTTGATCTGTTCTTGATTTC

TAAGGTGGAGCTTTCTTGTTGACACGTTTGATTGATTTCTGATACATAAA

TTCAAATATGTATGGTATCTGTACTAGCCAAGGAGTATTAAGAACTGTTC

CAATGTTCAGGATGTAGGAAAAGCCACTGAGAGTTTAGCTGCTAAAGATG

GAACTCTTCATGATTCAGAGGATTCTGCATGTGTACAAACAGCCAATGAG

GCGCACATGAATCCAGAAGTGGTATCGATGGATGAAAATGACAAATTCGC

AACAAGCAAACCCGAGAAAGAACAGAATGATGCACCCAACTCAAAAGAGA

AATTGAAGAAGCCGGACAAAATACTTCCATGCCCCCGCTGCAATAGTATG

GAAACCAAGTTTTGTTATTACAATAATTATAATGTCAACCAACCACGCCA

TTTCTGTAAAGCCTGTCAAAGATATTGGACTGAAGGCGGTACCATGAGGA

ATGTCCCCGTTGGAGCTGGCCGCCGAAAGAACAAGAACTCTGCCTCACAC

TACCGACAGATTACAATCTCTGAGGCTCTTCAAGCTGCACAAATTGATAT

TCCTAATGGAGTCAACTGCCTAGCAACCAAAAGCAATGGGCGAGTCCTAA

ATTTCAGTGTAAACGCACCTGTATGTGAATCTATGTCCACTGTCCTCAAT

CCGGCAGGAAGAAAGGTCTTGAATGGGACTAGGAATGAATTTCATAGACT

CGATGACCAAGGAATTAAAGCACCTTGCAAGGGTGGGGAAACTGGGGATG

ATTGTTCCAGTGCATCTTCAGTGACGATGTCGAGTTCAATGGAGGAAGGA

GCCAGGAGATGCCCTCAAGAACCACAGATGCAGAATATCAATGGTTTCCC

TCCTCAAATTCCATATCTCCCTGGTGTTCCTTGGCCTTGTTCATGGAATC

CACCAATGCCTCCACCGGCCTTCTGCCCTCCTGGAGTTCCTTTATCATTC

TATCCTGCGACATACTGGAGCTGTGGCGTTCCAGGTGCTTGGAATATTCC

TTGGTTCCCTCCACAACCCTGTTCACCAAATTCTGGTGCAAATTCACCTA

CACTGGGGAAGCATTCCAGAGATGGTGACAAACTCCAGGCTGATAATTCT

GAGAATGAAGAACCTCCAAAACAGAAAAACGGATCTGTTTTGATTCCCAA

AACTTTGAGGATTGATGATCCGGATGAAGCCGCTAAAAGTTCAATATGGG

AGACACTTGGTATTAAGAATGATTCAATCAAAGCTGTTGACCTGTCTAAG

GTCTTCCAATCAAAGGGTGATCAAAAGAATAGGGTTTCTGAAGTGTTATC

ACCAGTTTTGCAAGCTAACCCTGCAGCCTTGTCAAGATCTCTTACTTTCC

ATGAACGTTCGTGA

>ClDof16

ATGATGGTATGTCAAAATTCAAAAGATCAAATCAGAAAGCCAAGGCCACA

GCCAGAACAAGCCTTGAAATGTCCAAGATGTGACTCAACAAACACCAAAT

TTTGTTACTACAACAACTACAGCCTCTCTCAACCAAGATACTTTTGCAAA

TCCTGCCGCCGTTACTGGACACAAGGCGGCACTCTCCGCAATGTTCCAGT

AGGCGGAGGTTGTCGTAAAAACAAAAGATCATCCTCCACGAATTCATCAA

CCTCTTCCTCTAAAAAATCCCAAGACCATCCCTTTGGCTCCGGGGGCCTA

CCCCACTTGTCCTACGATCACCAAGCCCATGACCTAAGTTTGGCATTCGC

AAGGCTTCACAAAAACTCGTGTGGTAGTGGGAGTGGACCCGCATTCAACG

ACTTCGATTTCTCCATTTTGGGAATGCCAAACGGCGACGTTTTGAATGGC

TTTGGGTATAATTCTCAAAGCATGTATTATGGAAATGACAACAACATGGG

AGGAATCGAAAGTAATGGAGATCAAATGAGATTACAACCGTACGATCAAG

ATCATCATCATCATCATAATCAACAGTATAGCAATGCTACAACGACGGCA

GTGACGGTGACAACGATGAAGCAAGAGCTATTTGGAGGACGAGACATCAA

TGGAGGTGACCAAAGTAAAATCCTTTGGGGATACCCAAATTGGCAAATGA

ACAATAATAATAATGCAATAGATTCAAACACCACCACCACCACGACGATG

ATGGCAGCCATGGATTTCGATTCGGGTTCGGCTCGCGAAAGCTGGAACAA

CAATGCCTTCACTAACGCTTCTTCTTGGCATGGCCTTCTCAATAGTCCAC

TCATGTAA

>ClDof33

ATGTCGGAGCCAAAAGATCCGGCGATCAAGCTCTTTGGCAAGACGATTCC

TCTACCTGAGGCTTCTCCGGCCACTCCTCAGCTTCCATCTTCTTTACCTA

TTCTCACACCCGGTGACGATAACACCGCCGCTGTTGATCATGACCACGAT

TCTTCTTCTTCAAGCTTGTCCCCGGAAGCCAATATCGATGGGGATGCTGA

AGATCTGGAGGTCGATAAGGTATGGAGTTACCTTAAATCGATTCTTGTTT

TTTCGGATACCCATTTGCTTTATTGCCGATATCTTTGCTGCTTTTATATT

GCTTCTATTCATTTTGTTGACTTGATGTATGTTCATCTGTCCTAGGAAAT

TTAGGTTTCTTTTCTATTTGGTTTCTTCTTTGATTGTTTTGATTTGTGGG

GTTTTGTGAGATCTTTTTCTGTGATATTTCTGATTCTGTTATGAAGATTT

TGGATTTTGATTTTGTACTCAGATTTACCAATTTGGAAAGCCTGTTTTGT

TTACTTAATTTAGTGATGCAGGTACTTTTTTGCTGTAATTTCCATAATTG

AGCGTGAAGTTTTTTTAGAAATGCTGCTTTGATGCTTTCAGAATTTGGGT

TAAAGTTATTGAGTTTGTTAGGAGTTTATTGAGATATCTTTTGAACTTGT

GTCACTGTGGTAGAGGTTTGTTGCTTAAATTTGGTAATGTTGTTTTAATT

TTTTCATAAAGAATTTTGTTTGGTATTTGTGTTTTGGTGTATTAGGAGAC

TATAAGAGGAAAATCAGGTGGAACTAAGCTGGAAGATGGAGATGGAGATG

GAGATGGAGATGGAGATGGAGATGGAGATGGCGATGGAGATGGAGGCTTA

TCTGTTTCGACCGAAGAGTTTACTAATTCGGATACTTCTGCAGTGAGATC

AGAGAATTCTAAAGTACTTTCTGGTGAGGAAAGTAATCCGTCAAGTACTA

CAAAGACTGATGAACAGAATGAAACAAGTAACTCGCAAGAGAAGACTCTC

AAGAAACCCGACAAGATACTTCCGTGCCCCCGCTGTAACAGCATGGATAC

CAAGTTCTGTTATTATAATAACTACAATGTCAATCAACCGCGCCACTTTT

GTAAGAACTGTCAAAGGTATTGGACTGCTGGAGGGACAATGAGGAATGTT

CCTGTGGGTGCCGGACGTCGTAAGAACAAAAGCTCTGCTTCTCACCATCG

TCAGATTATTGTTTCAGAATCGCTTCAGCATGCTCGGACTGATGTTTCTA

ATGGTATCCATCATTCTGCCCTCAAACCTAATGGCAATGTATTGGCATTT

GGTTCAGATGCCCCTCTTTGTGAATCAATGGCTTCCATTTTGAACATTGC

TGATAAAACAAGGCAGAATAGTACTAGAAATGGTTTTCAAAAACCTGAAG

CTCTGAAAATTCCTGTGGCTTATGAGAATGGTGAAAATGATGATCAGTCT

CAGGAATCTGCTGCAACTCCATCATTTATAAATAATGAAGAAGGCAAAAC

TGGGCCACAAGATCAAGTGATTCATAATTGTCAAGGCTTTTTACCTCCTC

AGATTCCATTCTTCCCTGGGGCTCCTTGGCCTTACCCTTGGAATTCACCA

CAATGGAGCTCTCCTGTTCCACCTCCTACTTTCTATCCACCAGGCATCCC

TATGCCGTTCTATCCGACCGCCCCGTTTTGGGGTTGTACCGTTCCCGGCG

CCTGGACAGTCCCGTGGGTTTCCCAGCCACCATCTCTGAGCCCTGTTGCC

CAAAACCATGCTCCCAACTCTCCAACCTTGGGGAAACATTCAAGAGATGA

GAATGTGACCAAGCAAAGCGATTTTGGGGAAGACGAGCAGCAGAAAGATA

ACAAAGCTGAGAGATGTCTTTGGATACCAAAAACGTTGAGGATTGACGAC

CCCGGCGAAGCAGCTAAAAGCTCCATATGGGCAACTCTGGGAATAAAAAA

TGACAAGACTGATTCAGTCAGTGAAGGGCTTTTCAAGGCTTTTCAATCAA

AGAAGACCAACGGAAAGAATCACAAAGCCGAAGCCTCTCCAGTCTTGCAA

GTCAATCCAGCTGCATTGTCCAGATCAATCAAATTTCATGAAAGCTCGTA

A

>ClDof23

ATGGATTCTGCTCACTGGCCTCAGGTATGGCTCATTGAACCCTTCTCTCT

TTTTCTAGTCTCTCTCAATGATCAGTTCTTATAATCTATTTGAGTGTTCT

TGTGATGAATATTGGGTGATGCAGGTTGAAGTTAAAAGCATGGAATTAGA

AGAAGAGGGAATAAAGGCAGTTGTGGAAAGAAAAGCAAAGGCTAGGAAAG

ATCAAATATTGAACTGCCCAAGATGCAATTCCAACAACACAAAGTTCTGT

TACTACAACAATTACAGCCTTTCACAGCCAAGATACTTCTGTAAGTCTTG

TAGAAGATATTGGACAGCAGGTGGGTCTTTGAGGAACATTCCAGTAGGTG

GGGCCTCCAGGAAGAACAAGAGACCTTCAGCTAATTTTTCATCACCTCCT

TCAAAGAACAATCAAAAGAACTGTTATAACGATAACAACAATAATGGTGA

TGATGATGATCATCAAGGCATTTCCCAATTGAATATCAATACTACTTGTT

CTTCAACTATTACTAATACTGCTACTTCTTGTTGTTGGCTGTCATCTGAT

GATCATATGAATAATCAAATAATGCTGAGAAGTAGTGGAATAATGTCTCA

AAGGGAGCTGATTCCTTTTATTCCAATGCCTGCACCAGCTCCTCCACAGC

CCAGTACTGCTGCTGCTCTGGAGGATTTCAAGCAACTTTCAACTATCATT

TCTACTGATCAAAATGGAGCCAAATTGGGGGATGTTCCTGCCTTTTGGAA

TGGCATTTTTGGAGGAGGTTCATGGTGA

>ClDof17

ATGGGAGAGAGAAAGAATAATAATGAAGATCAATACGAAGGTGGAATTAA

GCTGTTTGGGGCAACAATTATGTTGCAAAATAACAGGCAAATCAAAGAAG

AAGAAGAAGAAGAAGAAGAAGAAGAGGCCAATAAATCAGATCAGCAATCA

CTAGAAAAAAGGCCTGAGAAGATCATCCCTTGCCCAAGATGCAAAAGCAT

GGACACCAAATTTTGTTACTTCAATAATTACAATGTTAATCAGCCAAGGC

ATTTCTGTAAGGGCTGCCAAAGGTATTGGACTGCCGGCGGGGCCCTTCGC

AACGTCCCCGTCGGAGCCGGCCGTCGCAAAACCAAGCCCCCTTGTCGGAC

CTTCGCTGGCTTGCCGGAAAATTGTGTCTTTGATTCTTCAGGGATCGTTG

CGGTTCAACCGTTTGAGTTGGAAGGGATGGTCGAGGAGTGGCATGTCGTC

GCTGCCACCGCCACACAAGGCGGTTTTCGACAAATTTTACCGGTAAAGCG

GCGGAGGGATTGCCAAGATGGCCAAACTTGTTGA

>ClDof28

ATGGTTTTTTCTTCTTCTCTCCCTTCTTATTTGGATCCTCCTAACTGGCA

ACCGGTAAGTCGCTGAATTCGAACCTACAACTTATAAAGAGAGAACAGAA

AAAAGAAGCTTTGTTTTTGTTAATTCTTTAGCTTCCTTCTTCTCCAAAAA

AGTTCGTTTTTATCGGAAAATATTCCGACCCTTTTTCGATCTCTTCTTGA

ATATACTCGATATTTAATCTTTCTTTGCGAATTTTTTGTTTTTTGTTTTT

GATTTTTTTTTAGTTGACGAATCACCACACGGGAGGAAATGATACTGCAG

AGGATTCTGGTCAAGTTTTACTACCTCCACCACCTGGGGGTGGGGGAGGT

AGTGACAGTGGTGGTGTTGGAGGTGGTACTGGGTCGATTAGGCCGGTTTC

GATGACTGATCGAGCCCGGTTGGCGAAGATTCCGCAGCCAGAGGCCGGCT

TGAAATGCCCTAGATGTGAATCTACAAATACCAAATTTTGTTACTTCAAT

AACTATAATCTTTCTCAGCCTCGCCACTTTTGTAAGACTTGTCGTCGGTA

TTGGACACGTGGTGGTGCACTGAGGAATGTTCCGGTCGGTGGTGGTTGCC

GGAGGAACAAGAGGACCAAATCCCGGAATCGGTCTAAGTCTCCGGTGGCC

GGTGAACGGCAATTATTAGGTGGATCAGCTAACTCTACTGCTGGGGTTTC

ACTTTCAACACAACCCCATGTGCCCTTTTTGTCTTCTTTACATAATTTCT

CTAATTATGGCCTCAATTTGGGCATAATTCCGCCGCAAGCTCCTCCATCA

AGCGGTGGCTCCACCACCGGAGGAGTGGATGTTCATGAATTTCAAGGTGA

CCATTGGAGATTACAACAACCACAACAATTCCCTCTCTTGGCTAATGATC

AACAACCTAATTTGCTTTACACTTTCGAGCCACCGGAAGGCAGCATGACT

CGGTATAATCTTGGCGGGTTCTCTAGATTAGGGATTGAAAACGACATGGG

AATGACGACGGAGGCTGGGGCGGTGAAGGTGGAGGAGAGTAAAGGAATGA

ACTTAGGGAAGAATTTTCAATTTTGGGTCGGTGGTGGTGGCGGAAGCGAC

GTTAATGCATGGAGCGGCGGTGGCGGCGATCTTCATGGCTTCTCTTCATC

CTCCGCCAGCCACTTATTGAGACAGTAA

>ClDof31

ATGCCGTCGGAATCAGCCAACAGAACCAACAGTAGGCCCCAACCCCAACA

CACCATGGGCTTCCCTCCCCCTCCCCAATCCGACCCTCTCCCCTGTCCTC

GCTGCGATTCCCTCAACACCAAGTTCTGTTACTACAACAACTACAACCTC

TCCCAGCCTCGCCACTTCTGCAAATCCTGCCGCCGCTACTGGACTCACGG

CGGCACCCTACGCGACGTTCCTGTCGGCGGCGGCTCCCGCAAGAACTCCA

AACGCTCTCGCTATCACAACATCAACAATACCCCTTCCTCTGCCTCTGCT

TCCTCTTCCACCTCTGTTTCTTCTTCCACCTCTGCTTCCTCTTCCTCCTC

TGTTTCTCTTCTCCCCACCGCCGCCGCCCTCAACGACATCAACGACACCA

TCCCTCCTCCTGGCACTTTTACCTCTTTGCTCAGCTCCCAAGCCTCCGGA

TTCTTAGCTCTTGGCGGTTACGCCTCTGCCTCTGGGTCGGGTCATCCCCC

TGCAGCGTTCGACGATATGGGGTTTGCCCTCGGCAGGGGATTGTGGGGTA

CTGCTTGTACTTACCCTGAAGTTGGGGATCTCGTCGGGAGTTACGCCGCC

GCTGGGGCTCCGGAGACGTCTTCTTCCGGGTACAACGCATGGCAGATGAA

TGCCACCGACGGCGGTGGTAACGGAGGAGTTGTCGATGGTGATTTGCTGG

GTTGGCCGGACCTTGCCATTTCCATGCAAGGCAAGAGCTTGAAGTAA

>ClDof30

ATGTCTGACCACCACCAGCAACTCTCCCACCCGCCGCCTCGTCTCCCTCC

TCCTCCGCCGCCATTCGCCTCCCATCTCGAACGAAAATGGAAGCCCCATC

TCGAAACAGCCCCAAATTGCCCCCGCTGCGCCTCCGCCAACACCAAATTC

TGTTATTACAACAACTACAGCCTCTCCCAACCCAGATACTTTTGTAAATC

CTGCCGCCGCTACTGGACCAAAGGCGGCTCCCTCCGCAACGTCCCCGTCG

GCGGTGGCTGCCGCAAGAGCCGCCGTGCCAAATCCTCAAGATCCTCCGCC

GTCTCCCGTCCTACAAAACCCGACGTTTCTACCCAAATCACCGACTCCTC

CTCCAATGGTTCCGATATCGATCTCGCCGCTGTTTTCGCCCGATTTTTGA

ACAGTCCCCCATGTAGTGAACCCCAGGCGTTGACCAGCTCGCCGGAATCG

GTTGACCCATTGGAGAATTCCGAGATCTTCTTCGAAGGTCTCTCAGATTT

GCTATTCGACGAAGAAAATCAAGGCCAAGAAGAAAAACAGGGGATCCCCG

GAAATTACGAGAATCTTCCATATGGGTTAGAAACAGAGTTGGGCATTGAT

GAAGAACTATGGCCATCACATCAAATGCAAGAACAACTTGAAGAATTGGA

CTCATTTTCTTGCAATTACAATGCGAATCATTTTCGGGTCTCGGATCAAT

TCTGTCAAGTGGGGGATAATTGGAGTTCATTCGACTTTGCAGCCGTAAAT

AATGTCGAATATTTCTAA

>ClDof5

ATGGGTCTTACTTCTCTTCAAGTCTGCATGGACTCTTCTGATTGGCTACA

GGTATATCTATATACACACATGAATGAGAAACAATGTCAGTAACTCTAAG

CTCATCATAGCTTCAACTATCAAAAACAGAGCAAATGTACAATGTTTATG

TGTGTGACACGATATAATATTAAATCTATCTTCTTCCATCAGCTTAAGTT

TTTGGTTCATTGACACGGTTTTTTTTTTTTTTTTTTTTTTTTTTGTTGGT

TTTAGGGTACTATAAACGAGGAGTCAGGGATGGATTCTTCTTCATTATCA

GGAGATATGCTTTCATGTTCAAGGCCATTAACAGAGAGGAGACTAAGGCC

ACAACATGATCAAGCTCTGAAATGTCCAAGATGTGATTCAACTCACACCA

AATTTTGTTATTACAACAATTATAGTCTTTCCCAGCCTCGTTACTTTTGC

AAAACTTGTCGTAGGTATTGGACTAAAGGAGGAACTCTAAGAAACATTCC

AGTTGGTGGTGGCTGTAGAAAGAACAAGAAAGTTTCCACTACTAAAAAAT

CCAATGATCAACAACAACAACAACAACAACAACAACCACAAATTCAACAA

CCCATTTCTCAAAATCATCATCATCTTCTTCATCATCATCATCATCATCA

TGGCCCTTCTTCTTCTTCTTCTTCTTCTCTTCACATTCACAATCCAACTG

ATCTCCATCTCTCATTTCCAGACCAAGTTCAATTCCCTCATTTCAATCCT

CTCATTGGAACAACTCCTCCAAATTTCACCATTGGAATGCTTGAAACTCA

TCACCATCATCATCAAACTAGACCCATTGATTTCATGGACACTAAAATGG

AAGCCATTGTTGGGAATAATGGTCATTATTCCACAAACACTGATCATTTA

GCTATGGTTGGAGGGTTAAATGGAGATCATCATATCACAGCTGCTGCAAC

AAATTTCCATGGTCTTTGTTCTCCTTATGGACTATCACTTGATGGGAATA

TTAACCAAATGATGATTCCTTATGATCAACATCAAACAAATGAAGATCCA

AATGGAATGGATGTGAAACCAAATACAAAGCTTTTGGCTTTGGAATGGCA

AGATCAAGGATGTTCAGAGAAAGTTGAATCTTATGGATATATAAATGGAA

TTGGGTCTTCTTGGAATGGGATGATGAATGGATATGGACCATCAACAACC

AACCCATTGGTGTAA
