# Supplementary figures and images for: Genome-wide characterization and expression analysis of the Dof gene family related to abiotic stress in watermelon
Source: PeerJ. 2020 Feb 17;8:e8358. doi: 10.7717/peerj.8358 (PMC7032062; doi:10.7717/peerj.8358)

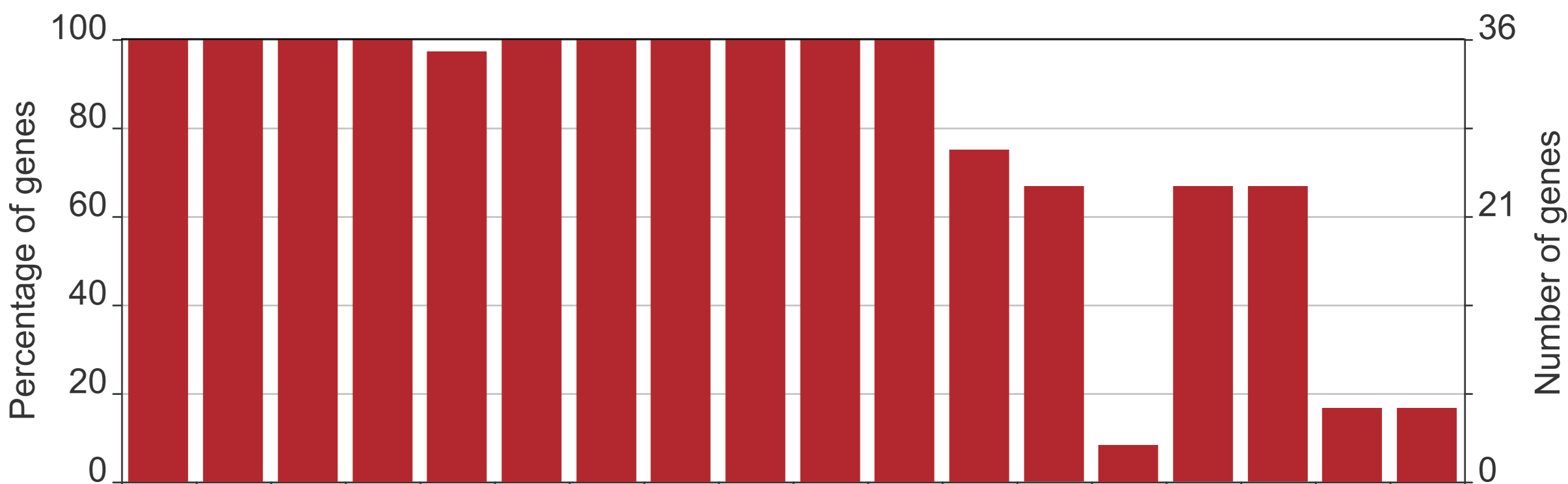

Cellular Component      Molecular Function      Biological Process

Supplement: Supplemental Information 8 — Three categories, including cellular component, molecular function, and biological process, were identified and visualized with the WEGO program. [file peerj-08-8358-s008.pdf]
